# Supplementary material for: Providing psychological support to people in intensive care: development and feasibility study of a nurse-led intervention to prevent acute stress and long-term morbidity
Source: BMJ Open. 2018 Jul 23;8(7):e021083. doi: 10.1136/bmjopen-2017-021083 (PMC6059275; doi:10.1136/bmjopen-2017-021083)
Supplement: Supplementary file 1 [file bmjopen-2017-021083supp001.pdf]

Electronic supplement one: Development phases of an intervention to reduce patient stress in critical care

MRC model – steps and stages	Preparatory phase 2006-11*	Early modelling 2011-13†	Feasibility and refinement phase 2013-2015‡
Stage one, step one<br>Identify the evidence base	Identified systematic reviews and studies of psychological outcomes, risk factors and conducted new systematic reviews of post-traumatic stress, depression, anxiety post-ICU[1]<br><br>Literature review of ICU stressors, reactions, memories, stress measurement and stress reduction interventions[1]	Updated evidence base - identified new systematic reviews and studies of ICU psychological outcomes, risk factors, stress, stress measurement, stress reduction interventions[7,20,22]<br><br>Began systematic review of psychosocial interventions (psychological, music, mind-body therapies) to reduce ICU-related stress[24,35]	Updated evidence base of studies and systematic reviews[8,12,33]<br><br>Completed systematic review of interventions to reduce critical care stress[24,35]
Stage one, step two<br>Develop a theoretical understanding of determinants and processes of change	Patient studies: acute stress in ICU and psychological outcomes:<br>1. Quantitative study (n=40)[31]<br>2. Quantitative study (n=157)[7]<br>3. Qualitative study (n=7)[31]<br>4. Qualitative study (n=17)[33]<br><br>Developed hypotheses about causes of critical stress[1,7,31,32]<br><br>Explored nurse perceptions of patients' psychological needs and nurses' ability to attend to them: Content analysis of 5 focus groups at GSTT NHS trust with 125 nurses[32]<br><br>Identified communication[37] and adult learning methods** to inform development of training to enhance critical care nurse communication and psychological skills	Patient and relatives advisory group set up at UCLH; regular meetings to brainstorm and generate research ideas about patients' psychological needs and how they could be met<br><br>Improved conceptualisation and measurement of acute ICU stress: Development and validation of the Intensive care psychological assessment tool[34]<br><br>Identified psychological approaches (to stress and coping[36], psychosis[25-26] and trauma[27-28]) that could be adapted for critical care patients<br><br>Identified appropriate relaxation and recovery methods	Identified behaviour change methods[39] relevant to changing staff behaviours to create a therapeutic environment<br><br>Identified clinical supervision methods for non-experts delivering psychological support[40]
Stage one, step three<br>Model process and outcomes	Developed, piloted and evaluated a training day at GSTT to train critical care nurses in communication/psychological skills (utilising actors)[32]	Consultation with experts and discussions between UCLH, ICNARC and patient representatives, to develop the basic structure of a psychological intervention consisting of three interconnected elements: Creating a therapeutic environment; three stress support sessions; and a relaxation and recovery programme. Each element to have associated training components (online, face-to-face and training materials)<br><br>Early development of stress support sessions based on psychological principles at	Formed Expert Psychological Advisory Group of medical educationalist, psychologists, patients and the research team, to inform the final phase of POPPI†† intervention development<br><br>Further development of stress support sessions by senior nurses and psychologists<br><br>Created materials to deliver a complex intervention - nurse manual, checklists, notes and summaries<br><br>Researched/created content for and design of a relaxation and recovery app, a DVD and a

		UCLH – iterative process with psychologist and patients, supervised by senior psychologists (CB, JW, LML and staff from PICUP)<br><br>Identification of suitable materials for the relaxation/recovery programme<br><br>Post-traumatic stress selected as primary outcome (based on evidence identified in step one – more evidence of risk factors than for other psychological outcomes)	psychological well-being booklet for patients<br><br>Created training courses and materials for staff to deliver the psychological intervention:<br>Online training course, all staff<br>3-day face to face course for POPPI nurses, feedback/assessment day , training folder<br>A programme of debriefing and support for POPPI nurses
Stage two Feasibility and Piloting		Designed two separate feasibility studies – intervention feasibility study and the trial procedures feasibility study	Conducted two feasibility studies – intervention feasibility study and trial procedure study<br><br>Refined the complex intervention based on quantitative and qualitative data from patients and staff in the intervention feasibility study

**Kings College London, Guys and St Thomas hospitals Trust, University College/University College Hospital, London*
†University College Hospital (UCH) and Intensive Care National Audit and Research Centre (ICNARC);
‡ ICNARC and UCH
*** 43. Kolb DA. Experiential learning: experience as the source of learning and development. 1 ed. Englewood Cliffs, NJ: Prentice Hall, 1984.*
†† POPPI – Provision Of Psychological support to People in Intensive care
CB Chris Brewin (author), JW John Weinman (author) LML Lih-Mei Liao, Consultant Clinical Psychologist, University College London Hospitals (UCLH) . PICUP -Psychological interventions clinic for outpatients with psychosis, Maudesley Psychology Centre, Maudesley Hospital, London
