# Supplementary material for: Providing psychological support to people in intensive care: development and feasibility study of a nurse-led intervention to prevent acute stress and long-term morbidity
Source: BMJ Open. 2018 Jul 23;8(7):e021083. doi: 10.1136/bmjopen-2017-021083 (PMC6059275; doi:10.1136/bmjopen-2017-021083)
Supplement: Supplementary file 2 [file bmjopen-2017-021083supp002.pdf]

Electronic supplement two

This supplement presents further details of the final post-feasibility version of the three-element POPPI complex intervention. This version is being evaluated in the POPPI cRCT.

Element one: Creating a therapeutic environment

Online training course
A key part of element one is the POPPI online training course: Key skills in Psychological Care. Training is undertaken by all clinical critical care staff to aid the creation of a calm, less stressful environment by reducing stressors in the environment and using good communication with patients. The course takes about 30 minutes and comprises the following five sections:

1 Understanding the stresses of intensive care patients
2 Reducing stress and fear in patients
3 Communicating with distressed patients
4 Inspiring patients with confidence and hope
5 Summary and assessment

The course has a balance of concise, readable text with graphics and other visual or audio aids, and test-yourself questions at the end of each section (with informative feedback). Video clips include former patients talking about their experiences of critical care, and nurses modelling good communication strategies with patients. Staff who pass the assessment (a score of 80% or more) receive a certificate.

Element two: Three stress support sessions

Three, one-to-one, stress support sessions lasting approximately 30 minutes each, are delivered to a patient by the same trained POPPI nurse, starting when patients are awake and alert, either in the critical care unit or following discharge to the hospital ward. The three sessions should be delivered within a week (for the pragmatic reason that many patients may be discharged from hospital around that time).

All stress support sessions contain three common components – starting the session; building rapport and finishing the session - and three individual components per session. Starting a session includes introductions, outlining aims, measuring stress and explaining confidentiality, note-taking and risk-reporting. Building rapport involves focusing throughout all the sessions on creating a relationship with the patient based on openness, honesty, warmth and listening. Finishing a session involves summarising, getting patient feedback, agreeing practice, re-measuring stress, agreeing wording for notes and arranging the next session.

The three individual components nurses deliver in session one are: Normalise reactions (discuss common psychological reactions in critical care and possible reasons); Encourage communication (help patients open up about worries and concerns), and Teach coping strategies, e.g. patients gathering more information and practising relaxation exercises (acronym NET). The second session comprises: Stress reactions (encourage further expression of worries and notice stressful thoughts); Explain stressful thinking (show how unrealistic fears can create extra stress), and Teach 'check out my fear', a technique to understand how accurate fears are (SET). The third session includes: Summarise and review (key messages from sessions and persisting issues); Action plan (personal planning to build on sessions and cope with challenges ahead) and Future Expectations (reinforce progress made and encourage realistic optimism for psychological and physical recovery (acronym SAFE).

Education package to deliver element two

The education package to train POPPI nurses to deliver element two includes a central three-day face-to-face training course with associated materials; a local debriefing and support visit, including a skills development assessment, and ongoing debriefing and support phone calls from trainers.

The course involves pre-course study (a psychological principles booklet, a manual for the three stress support sessions and access to the POPPI online training course) and a three-day programme, as follows:

Day one

- Understanding critical care patients' stress
- Learning the skills needed to deliver stress support sessions
- Overview of the stress support sessions
- Observing and practising session one

Day two

- More about unusual experiences in critical care
- Learning further stress support skills
- Observing and practising stress support session two

Day three

- Revision of key points from stress support session two
- Observing and practising stress support session three
- Using the patient booklet to create personal action plans
- Debriefing and support arrangements

Course materials include training folder with day 1-3 course handouts, word and visual summaries of each session, checklists and reflective notes for each session, stress thermometer tool, copies of patient booklet and DVD.

Debriefing and support

The first debriefing and support call should occur soon after (or during, if nurse requests) the three stress support sessions nurses deliver to their first POPPI patient. Subsequent calls are every two months, or on nurses' request. The focus of these calls is on enhancing nurses' skills and discussing patient cases. Emails can be exchanged between nurses and trainers about issues arising from stress support sessions at any time. Monthly teleconferences with POPPI nurses are facilitated by a trainer to encourage peer support.

A debriefing and support visit is made by an allocated trainer to offer the three POPPI nurses at one site further support, both individually and in a group. Later in the visit a skills development assessment is carried out for each nurse. Using a scenario, the nurse delivers session 2 to the trainer who plays the role of the patient. The trainer rates the nurse's skills using a checklist. The pass mark is 8/16. Nurses are required to pass the assessment, to continue delivering stress support sessions to further patients.

Element Three: Relaxation and recovery programme

In addition to stress support sessions, patients receive a "relaxation and recovery" programme (using a tablet computer app between sessions, and a DVD and patient booklet to take home after hospital) to help them to sleep and cope better with stress. Former intensive care patients' recovery stories are included to help normalise critical care experiences and reactions, and encourage hope and optimism for recovery. Contents also include audio or video files of a safe place visualisation, muscle relaxation and breathing exercises; a body scan and other mindfulness meditations; relaxing classical music from Bach to Vivaldi, calming modern ambient music; and restful nature sounds and scenes.

The programme was first developed as an application for tablet computers. The design of the app includes a green nature-scene background and large coloured buttons for patients to use for easy navigation between different sections. The tablet computers should be checked for approval by hospital infection control teams. DVDs include a shorter selection of relaxation exercises and music from the app, due to restricted space. However they include longer versions of the patient recovery stories, assuming longer concentration spans as patients get closer to hospital discharge or go home.

The “Getting well, staying well” booklet provides patients with information on making a good psychological recovery after a critical care stay. It builds on the support patients received during the stress support sessions, focusing on well-being and coping strategies. Contents include information about what to expect in the early days (leaving critical care, returning home; emotions after critical care, relationships), tips for psychological wellbeing; advice on coping with difficulties (worries, panic, low mood, memories); information on sources of psychological support; further information for family and friends and a personal action plan to promote well-being and recovery.
