# Supplementary material for: Providing psychological support to people in intensive care: development and feasibility study of a nurse-led intervention to prevent acute stress and long-term morbidity
Source: BMJ Open. 2018 Jul 23;8(7):e021083. doi: 10.1136/bmjopen-2017-021083 (PMC6059275; doi:10.1136/bmjopen-2017-021083)
Supplement: Author's manuscript [file bmjopen-2017-021083.draft_revisions.pdf]

BMJ Open is committed to open peer review. As part of this commitment we make the peer review history of every article we publish publicly available.

When an article is published we post the peer reviewers' comments and the authors' responses online. We also post the versions of the paper that were used during peer review. These are the versions that the peer review comments apply to.

The versions of the paper that follow are the versions that were submitted during the peer review process. They are not the versions of record or the final published versions. They should not be cited or distributed as the published version of this manuscript.

BMJ Open is an open access journal and the full, final, typeset and author-corrected version of record of the manuscript is available on our site with no access controls, subscription charges or pay-per-view fees (http://bmjopen.bmj.com).

If you have any questions on BMJ Open's open peer review process please email info.bmjopen@bmj.com

BMJ Open

Provision of psychological support to people in intensive care (POPPI): Development and testing of a nurse-led complex intervention

Journal:	*BMJ Open*
Manuscript ID	bmjopen-2017-021083
Article Type:	Research
Date Submitted by the Author:	12-Dec-2017
Complete List of Authors:	Wade, Dorothy; UCLH NHS Foundation Trust, Critical Care<br>Als, Nicole; UCLH NHS Foundation Trust, Critical Care<br>Bell, Vaughan; Maudesley Hospital, Psychological interventions clinic for outpatients with psychosis, Maudesley psychology centre<br>Brewin, Chris; University College London, Subdepartment of Clinical Health Psychology<br>D'Antoni, Donatella; King's College London, Institute of Pharmaceutical Science<br>Harrison, David; ICNARC,<br>Harvey, Mags; UCLH NHS Foundation Trust, Critical Care<br>Harvey, Sheila; ICNARC<br>Howell, David; UCLH NHS Foundation Trust, Critical Care<br>Mouncey, Paul; ICNARC, Clinical Trials Unit<br>Mythen, Monty; Institute of Sport Exercise and Health, 170 Tottenham Court Rd, University College London/University College London Hospitals, NIHR Biomedical Research Centre<br>Richards-Belle, Alvin; Intensive Care National Audit & Research Centre, Clinical Trials Unit<br>Smyth, Deborah; UCLH NHS Foundation Trust, Critical Care<br>Weinman, John; Kings College London, Institute of Pharmaceutical Sciences & Institute of Psychiatry<br>Welch, John; University College London Hospitals NHS Foundation Trust, Critical Care Department<br>Whitman, Chris; UCLH NHS Foundation Trust, Critical Care<br>Rowan, Kathryn; Intensive Care National Audit and Research Centre (ICNARC),
Keywords:	Adult intensive & critical care < ANAESTHETICS, Adult psychiatry < PSYCHIATRY, Depression & mood disorders < PSYCHIATRY, EDUCATION & TRAINING (see Medical Education & Training), Delirium & cognitive disorders < PSYCHIATRY

SCHOLARONE™
Manuscripts

Title

Provision of psychological support to people in intensive care (POPPI): Development and testing of a nurse-led complex intervention

Authors

Dorothy Wade, Health psychologist[1]

Nicole Als, Patient representative[1]

Vaughan Bell, Clinical psychologist[2]

Chris Brewin, Professor of clinical psychology[3]

Donatella D'Antoni, Psychology research assistant[4]

David Harrison, Head statistician[5]

Margaret Harvey, Patient representative[1]

Sheila Harvey, Senior research fellow[5]

David Howell, Divisional clinical director[1]

Paul Mouncey, Senior trial manager[5]

Monty Mythen, Professor of anaesthesia and critical care[6]

Alvin Richards-Belle, Trial manager[5]

Deborah Smyth, Senior research nurse[1]

John Weinman, Professor of health psychology as applied to medicine[4]

John Welch, Consultant nurse, critical care and critical care outreach[1]

Chris Whitman, Patient representative[1]

Kathy Rowan, Director of scientific and strategic development/clinical trials unit director[5]

1. Critical care department, University College London Hospitals NHS Foundation Trust, 235 Euston Rd, London NW1 2BU
2. Psychological interventions clinic for outpatients with psychosis P079, Maudesley Psychology Centre, Maudesley Hospital, Denmark Hill, London SE58AZ
3. Research department of clinical, educational & health psychology, University College London, Gower Street, London WC1E 6BT
4. Institute of Pharmaceutical Science, King's College London, Franklin-Wilkins Building, 150 Stamford Street London SE1 9NH
5. Intensive Care National Audit & Research Centre, 24 High Holborn, London WC1V 6AZ
6. University College London/ University College London Hospitals NIHR Biomedical Research Centre c/o Institute of Sport Exercise and Health (ISEH) 170 Tottenham Court Road London, W1T 7HA

Correspondence to: Dorothy Wade, Critical Care department, T03, University College Hospital, 235 Euston Rd, London NW1 2BU. Email: Dorothy.Wade@nhs.net.

For peer review only - http://bmjopen.bmj.com/site/about/guidelines.xhtml

ABSTRACT

Objectives Adverse psychological outcomes, following stressful experiences in critical care, affect more than 50% of patients. We aimed to develop and test the feasibility of a psychological intervention to reduce acute stress in critical care and prevent future morbidity.

Design We used the first two stages of the Medical Research Council framework for developing and evaluating complex interventions. Stage one (development) involved identifying existing evidence, developing and identifying theory, and modelling process (creating and testing elements of a psychological intervention in one unit). Stage two (feasibility) comprised a mixed-methods intervention feasibility study and trial procedures cohort study.

Setting Four general adult critical care units in the UK.

Participants Stage one: Former and current critical care patients; psychology, nursing, education experts. Stage two: Current patients and staff.

Outcomes Feasibility and acceptability to staff and patients of content and delivery of intervention, measured with quantitative and qualitative data. Recruitment and retention rates.

Results Building on previous work, we standardised an intervention for nurses to provide psychological support to people in intensive care (POPPI). We devised courses and materials to train staff to create a therapeutic environment in their units, to identify patients with acute stress, and to deliver three stress support sessions and a relaxation and recovery programme. 127 awake, orientated patients took part in the intervention feasibility study in two units. Patient and staff data indicated the psychological intervention was acceptable and feasible. Feedback was used to refine the intervention. 86 (54%) eligible patients entered the trial procedures study in two other units. 66 (80%) surviving patients completed questionnaires on post-traumatic stress, depression and health five months after recruitment.

Conclusion The 'POPPI' psychological intervention to manage acute stress in critical care and prevent future morbidity was feasible and acceptable. It was refined for evaluation in a cluster-randomised clinical trial.

Strengths and limitations of this study

The POPPI intervention built on a doctoral research programme investigating psychological risk factors, outcomes and interventions in critical care.

The POPPI intervention was developed using an up-to-date MRC framework by a clinical research team including patient survivors, overseen by an advisory group of psychological, nursing and education experts

More than 200 patients and 280 staff were involved in two feasibility studies.

For peer review only - http://bmjopen.bmj.com/site/about/guidelines.xhtml

The intervention was piloted in only two sites and there was limited patient feedback on the intervention.

No efficacy data were collected, given these were feasibility not evaluation studies.

For peer review only

For peer review only - http://bmjopen.bmj.com/site/about/guidelines.xhtml

INTRODUCTION

More than 170,000 patients are admitted to adult, general critical care units in the National Health Service (https://www.icnarc.org/DataServices/Attachments/Download/a30185e2-0e19-e711-80e6-1402ec3fcd79) in England, Wales and Northern Ireland each year. Although medical advances mean that increasing numbers of people survive, there is evidence that many critical care patients develop both acute and long-term distress.[1,2] Critical care units are stressful places, where patients suffer symptoms such as pain, thirst, nausea, fatigue, and disorientation associated with critical illness, invasive medical procedures and side-effects of potent drugs.[3, 4] Being attached to machines, tubes, masks and other equipment in a busy, noisy ward, often without access to daylight, windows or clocks, leads to sensory overload, sensory deprivation, and sleep disruption.[5] Patients are often isolated, unable to communicate, but aware of other people's suffering or death. Unsurprisingly 45%-80% of critical care patients experience acute stress in the form of panic, fear, depressed mood, anger or frightening hallucinations or delusions.[1, 6, 7] Furthermore, studies indicate that more than 50% of patients develop symptoms of severe anxiety, depression or post-traumatic stress, including hallucinatory, delusional flashbacks in the months after leaving a critical care unit.[8-12]

A National Institute for Health and Care Excellence (NICE) guideline[13] recommended that critical care patients suffering acute stress should be identified and offered psychological support in their recovery plan. 2017 NICE quality standards have been implemented to strengthen these requirements.[14] However due to a lack of evidence about what helps, little is currently done to alleviate critical care patients' stressful experiences. Sedatives or antipsychotics are often prescribed, but they have potentially harmful side-effects, both physical and psychological. There is an urgent need for psychological interventions to be developed and tested in the critical care setting.[15]

Outpatient follow-up services for critical care survivors have been evaluated[16-18] but clear evidence that they reduce psychological morbidity is lacking. Patients who suffer acute stress in the critical care unit are known to be at higher risk of future psychological morbidity.[10 19-23] Therefore interventions to address stress as early as possible could be more effective than follow-up services. Evidence exists that psychological and mind-body techniques can reduce critical care patients' stress,[24 25] but more robust research is needed to promote changes in clinical practice. Post-doctoral work by one author (DW) suggested that psychological techniques used for people with symptoms of anxiety, psychosis or post-traumatic stress could prove effective for critical care patients. These cognitive-behavioural techniques were adapted, streamlined and used in psychologist-led sessions in the University College Hospital, London (UCH) critical care unit (see table one and Electronic supplement one). However, as psychologists are a scarce resource in critical care units, it is more pragmatic to train experienced nurses to provide psychological interventions to patients. Therefore we aimed to standardise a nurse-led psychological intervention (Provision of psychological support for people in Intensive care – POPPI), to create an intervention education package for staff, conduct feasibility studies, and refine the intervention for evaluation in a randomised clinical trial (https://www.journalslibrary.nihr.ac.uk/programmes/hsdr/1264124/#/).

Table one: Development of an intervention to reduce critical care patients' stress: from inception to feasibility testing

MRC model - steps and stages	***Preparatory phase 2006-11****	***Early modelling phase 2011-13†***	***Feasibility and refinement phase 2013-2015‡***
Stage one step one, Identify evidence base	• Retrieved studies and systematic reviews of psychological impact<br>• Conducted systematic review of risk factors, outcomes	• Conducted systematic review of stress-reducing interventions	• Updated evidence: risk factors, outcomes, interventions
Stage one step two, Develop and identify theory	• Conducted empirical studies of patient experience, stress, risk factors, outcomes<br>• Content analysis: Focus groups on nurse perception of patient stress and their own ability to help<br>• General/post-traumatic stress theories<br>• Communication/adult learning theories	• Meetings with patient public involvement group to inform psychological intervention<br>• Development and validation of psychological assessment tool (IPAT)<br>• Cognitive-behavioural theories of psychosis and post-traumatic stress<br>• Coping theory: music, relaxation, mindfulness	• Behaviour change theory (COM-B)**<br>• Theory informing debriefing/support of non-expert staff delivering psychological care
Stage one step three, Model process and outcomes	• Psychologist-led team created communication and psychological skills nurse training day<br>• Selected feasibility outcomes (nurse satisfaction, learning, confidence in skills)	• Clinicians, researchers and patient group shaped an intervention with three elements<br>• Elements: therapeutic environment; patient stress support; relaxation and recovery<br>• Health psychologist developed stress support sessions with patients<br>• Selected feasibility and trial outcomes (PTSD, depression, quality of life)	• Senior nurses piloted stress support sessions with patients and trained as trainers<br>• Created patient recovery app, dvd, booklet<br>• Devised online and face-to-face staff training courses, training folder and manual<br>• Planned debriefing/support programme<br>• Standardised all elements of intervention with expert psychology advisory group (EPAG)
Stage two, Feasibility and piloting	• Evaluated feasibility of nurse communication and psychological skills training day		• Conducted intervention feasibility study in two sites and trial procedures feasibility study in two different sites<br>• Refined intervention based on feasibility data.<br>• Anxiety added as a trial outcome

*Professional training at Kings College London and St Thomas's hospital, London and PhD at University College London/University College Hospital (UCH), London †Post-doctoral work at UCH/UCL leading to grant application with Intensive Care National Audit and Research Centre (ICNARC); ‡Grant-funded work at ICNARC and UCH **COM-B: Capability, opportunity, motivation-behaviour model.

For full, referenced version of this table see Electronic supplement two

For peer review only - http://bmjopen.bmj.com/site/about/guidelines.xhtml

METHODS

We used the Medical Research Council (MRC) framework (see figure one) to develop and evaluate complex interventions.[26] This paper covers the work to complete stage one (development) and stage two (feasibility/piloting) of the MRC model. Stage three (evaluation of clinical and cost-effectiveness in a full trial) is continuing, and will be reported separately.

Development (stage one)

Stage one of the MRC frame-work includes three steps: Identifying the evidence base; identifying theory; and modelling process and outcomes. To complete this stage, we updated the evidence base on critical care-related psychological risk factors, outcomes and interventions that DW identified in previous doctoral work.[1,27] Two authors (DW and JWei) conducted a systematic review of psychological interventions to reduce acute or long-term stress in critical care patients.[25] To supplement previous theoretical work in identifying relevant psychological techniques and staff training methods (see introduction and table one) we conducted literature searches to inform other aspects, such as behaviour change theory and models of psychological supervision of non-expert staff. Modelling the intervention continued at UCH.

We pulled together the work strands on evidence, theory and modelling to standardise a nurse-led psychological intervention; create an education package for staff to deliver the intervention; and select study outcomes using the evidence base. Standardisation was an iterative process to test and revise the complex intervention, by psychologists, nurses, and patients, supervised by an expert psychological advisory group. The education package was also created in consultation with the advisory group.

Feasibility and piloting (stage two)

Once development of the complex intervention was completed, two feasibility studies were conducted simultaneously, following a favourable ethical opinion from the National Research Ethics Service Committee South Central - Oxford B on 23 April 2014 (14/SC/0149). An intervention feasibility study was held in two English adult general critical care units and a trial procedures study was held in two different units.

The objectives of the intervention feasibility study were to test the feasibility and acceptability of content and delivery of the complex intervention, and to get feedback from patients and staff, to help refine the intervention prior to full evaluation. Each site was asked to send three “POPPI” nurses to attend a three-day face-to-face course to learn about content and delivery of the intervention. During a “transition” month following the course, all clinical staff at the two units were asked to complete an online training course on creating a therapeutic environment, and patient recruitment began.

Inclusion criteria for patients were: Age 18 or greater; receipt of Level 2 or 3 care for 48 hours or more; Richmond Agitation Sedation Scale (RASS)[28] score between +1 and -1; English-speaking and ability to communicate orally; no pre-existing cognitive impairment, psychotic illnesses or chronic PTSD; Glasgow Coma

For peer review only - http://bmjopen.bmj.com/site/about/guidelines.xhtml

Scale (GCS) score of 15; not terminally ill or receiving end-of-life care. Patients were provided with written and verbal information as part of the informed consent process. Consenting patients were screened for acute stress with the Intensive Care Psychological Assessment Tool (IPAT), previously validated by our group.[29] Stress support sessions and a relaxation and recovery programme were delivered to acutely stressed patients by POPPI nurses.

Feasibility and acceptability of content and delivery of the intervention were assessed with quantitative and qualitative data from nurses. Once the intervention was embedded in the feasibility sites, we also sought views from patients who received the intervention. Quantitative data included delivery rates of each element of the intervention, as well as nurse ratings of satisfaction, self-efficacy (confidence in detecting and managing patients' distress) and learning[30] and patient ratings for satisfaction with the stress support sessions and relaxation recovery programme. Qualitative nurse data were gathered during focus groups, debriefing sessions and from case report forms (CRFs). Qualitative patient data came from patient satisfaction questionnaires and nurse-reported patient comments in the CRF. All data were used to refine the intervention.

The aim of the trial procedures feasibility study was to determine the estimated recruitment and retention rates for a full trial. Patient eligibility criteria were the same, and consent was obtained. At five months post-recruitment, patients were sent follow-up psychological and health questionnaires proposed for the full trial. These included the PTSD Symptom Severity Scale – Self Report (PSS-SR)[31] and the Centre for Epidemiologic Studies Depression Scale (short form) (CESD-10).[32]

Patient and public involvement

A group of former critical care patients and relatives, set up at the start of the research project, continued to inform development of the complex intervention. The group was diverse in age, gender, culture and critical care experiences, and members played a variety of roles. Three patients were members of an expert psychological advisory group, six were in the training team and seven were filmed narrating their experiences for the training courses.

RESULTS

Development (stage one)

Building on doctoral/post-doctoral preparatory and early modelling work by DW, development of the intervention was completed during the POPPI feasibility and refinement phase. See table one for a summary of the steps, stages and phases of the development work, which are explained more fully below.

Step one: The evidence base on psychological risk factors, prevalence and interventions was updated. A new systematic review[27] and a meta-analysis[33] confirmed median PTSD prevalence to be 20% of survivors, and strengthened the evidence that acute stress in critical care was an important risk factor for psychological morbidity,[10 19-21] as well as early intrusive or delusional memories.[22, 23] Several new studies found that

For peer review only - http://bmjopen.bmj.com/site/about/guidelines.xhtml

prevalence of all psychological morbidity was more than 50% of patients.[10-12] Early results from our systematic review of non-pharmacological interventions to reduce critical-care related stress were published.[34] 23 studies were identified, with 12 showing some beneficial effect. The quality and quantity of evidence was limited. However music, relaxation and psychotherapeutic approaches showed promise as elements of a complex psychological intervention.

Step two: Earlier work to develop a theoretical understanding of the proposed process of change (training staff to detect and reduce acute stress and early traumatic or delusional memories) included qualitative and quantitative work with patients and nurses [10 35-37]. This led to identification of theories relevant to mitigating acute stress,[38] processing traumatic or delusional memories,[39] and preventing PTSD.[40] In particular, CBT models of psychosis [41, 42] were identified as an appropriate framework. Adult learning theory[43] and training evaluation models[30] were identified to inform the education package.

To complete the theoretical work, we selected the COM-B (capability, opportunity and motivation) framework[44] to support behaviour change in critical care. The COM-B framework recognises that behaviour change results from shifts in factors such as people's physical and psychological capacity to carry out a behaviour, and barriers to changing behaviour. A significant behaviour change required to implement POPPI involves more effective communication between staff and patients, so we drew on current clinical communication models,[45] including therapeutic communication with patients who have unusual experiences such as hallucinations and delusions.[46] We chose the "developmental" model of clinical supervision for staff delivering POPPI,[47] as it is relevant to non-experts delivering psychological support.

Step three: To model process and outcomes, we standardised the complex intervention with three elements (see table two) and created an education package with associated materials to deliver it. The resulting POPPI intervention is described below. For more details of each element, see electronic supplement one (ES1).
In each unit three POPPI nurses were charged with ensuring that the IPAT was used routinely, encouraging staff behaviour change and overseeing delivery of the whole intervention. The first element of the intervention was for critical care staff to create a more therapeutic environment in their unit by improving communication with distressed patients and reducing stressors such as noise, unnatural light, insomnia, pain, and psychoactive drug effects. This was primarily delivered via online training (see below for course details). The POPPI nurse role included encouraging all staff to complete online training and reinforcing key learning objectives through ongoing education and bedside teaching.

For peer review only - http://bmjopen.bmj.com/site/about/guidelines.xhtml

Table two: The three elements of the POPPI psychological intervention

**Patient interventions**	**Who receives?**	**Where delivered?**	**Objectives**	**Theoretical approach**	**Training to deliver intervention**	**Who is trained?**
Element one: Creating a therapeutic environment in critical care	All critical care patients	In the critical care unit	Raise awareness of critical care stress<br>Reduce environmental stressors<br>Improve staff-patient communication<br>Encourage positive recovery messages	COM-B behaviour change framework<br>Psychological stress theories<br>Communication theories<br>Psychological first aid theory	An online training course<br>Promotional materials and local education sessions to reinforce key messages	All clinical critical care staff
Element two: Three stress support sessions for patients screened as acutely stressed	Patients screened by Intensive care psychological assessment tool (IPAT)[22] as acutely stressed (scores >7, range 0-20)	In the critical care unit, on wards following discharge from critical care	Expression of fears or concerns<br>Normalise stressful thoughts/feelin gs<br>Processing of traumatic memories<br>Testing fears or concerns<br>Encourage hope	Stress and coping theories<br>Adaptations of CBT techniques used in psychosis and trauma	A three-day face-to-face training course, to deliver stress support sessions, and the relaxation and recovery programme	Three “POPPI” nurses per critical care unit, selected by unit management with reference to suitability criteria
Element three: Relaxation and recovery programme on app, DVD and booklet (music, relaxation, meditation, patient recovery videos and self-help information)	Patients screened as acutely stressed and receiving stress support sessions	In the critical care unit, on wards (via tablet computer) and at home (via DVD and self-help booklet)	Reduce stress by promoting coping strategies<br>Watch patients’ recovery stories (normalise, promote optimism)<br>Create a personal plan for future well-being	Stress and coping theories<br><br>Social comparison theory<br><br>Psychological self-help theories	Provision of educational materials (pre-course theory booklet, manual and training folder) and ongoing debriefing and support from trainers	

For peer review only

The second element consisted of three 30-minute stress support sessions delivered by POPPI nurses to acutely stressed patients, ideally started in the critical care unit and continued throughout the hospital within a week. Acutely stressed patients were those who score more than 7 (range 0-20) using the IPAT.[29] The three stress support sessions were based on psychotherapeutic techniques evaluated in other populations and adapted for

For peer review only - http://bmjopen.bmj.com/site/about/guidelines.xhtml

critical care patients. Techniques from psychological interventions for psychosis included establishing a supportive, accepting, therapeutic relationship with patients; psychological education to reduce stigma and normalise unusual experiences (e.g. hallucinations and delusions); reducing distress associated with unusual experiences; cognitive and behavioural interventions to reduce distressing symptoms, and reducing relapse. Adaptations included simplifying cognitive and behavioural interventions to take account of critical care patients' likely physical, emotional or cognitive fragility.

Stress support sessions also included techniques from trauma-related CBT to help people to identify unhelpful thoughts, interpretations and coping styles, and to think about their experiences in a less upsetting way. However some components of CBT for trauma are not suitable for critical care patients. For example during stress support sessions, patients were encouraged to express their thoughts and feelings about their critical care experiences if they wish, but not to undertake 'reliving' of their critical care stay, particularly as their trauma may be continuing.

The main objectives of the stress support sessions were for nurses to develop a trusting relationship with patients, so patients could discuss concerns which they might feel embarrassed or worried about communicating, and to reduce emotional distress. Stress support session one involved discussing and normalising common psychological reactions in critical care, helping patients to open up about worries, and exploring coping strategies. In the second stress support session, patients were encouraged to open up more fully about their fears; to identify highly stressful thoughts, and to learn how to find out if their worst fears are realistic. The third session began with summarising key messages and reviewing remaining problems. Nurses then helped patients to make a personal plan to cope with challenges ahead, to build realistic optimism about recovery and plan where to seek help if problems arise in future. Patient stress levels were measured with a stress thermometer at the beginning and end of each stress support session.

The third element of the complex intervention was a relaxation and recovery programme designed to help people practise new coping strategies and learn from other patients' experiences, between stress support sessions. The POPPI app with music, relaxation, meditation, nature sounds/videos and patient recovery stories was loaded onto tablet computers loaned to patients receiving stress support sessions. DVDs with similar materials as the app, and a self-help booklet "Getting well, staying well" were given to patients to keep and take home.

An education package was developed to train staff in each element of the intervention. We adopted a blended learning approach, combining online training and face-to-face teaching.[48] Online training can convey knowledge to a large number of staff, particularly if training is interactive and engaging, while face-to-face training is more suitable when adults need to acquire new skills (by practising and receiving feedback). We adopted the Kirkpatrick model of evaluating training by measuring nurses' reaction, learning, behaviour and results.[30] The online training course to deliver element one, had four aims: to increase knowledge and

For peer review only - http://bmjopen.bmj.com/site/about/guidelines.xhtml

understanding of the psychological impact of critical care; to learn to reduce stressors in the environment; to improve staff communication skills; and promote realistic hope for patients' recovery. It was co-designed with education experts as an interactive course using videos, colourful graphics, and quizzes. At the end staff sit an online knowledge test and receive certificates if they pass.

Additionally a face-to-face course was devised to train POPPI nurses to deliver all elements of the complex intervention. During the three-day course, a significant amount of time was devoted to skills practice in delivering stress support sessions, with trainers, including patient representatives, observing and offering feedback. An additional day of feedback and assessment was held after POPPI nurses had returned to their unit and delivered sessions to a least one patient each. The day included focus groups on nurses' experience of delivering stress support sessions, and a skills development assessment, using a scenario in which trainees practised delivery of stress support session two to a simulated patient (actor). Trainers assessed trainees' competence using a checklist. Thereafter nurses received debriefing and support telephone calls from trainers at regular intervals.

Associated training materials were developed, including an intervention manual, a set of slides for the three-day course; and a training folder consisting of course handouts, patient scenarios, patient stress thermometers, summaries and checklists of each stress support session, and reflective note templates.

Key psychological outcomes were decided during the early modelling phase (see table 1) when we identified that PTSD and depression were the most serious common psychological outcomes of critical care. Work to identify modifiable critical care risk factors was more advanced for PTSD than for depression and anxiety, therefore PTSD was the primary outcome.

Feasibility and piloting (stage two)

Intervention feasibility pilot study

Patient recruitment was open for 5.5 months. 127 patients were recruited to the study, all were screened with the IPAT, and 51 (40%) were identified as acutely stressed (see figure two). 44 stressed patients (86%) received stress support sessions, with 39 (77%) receiving two or three sessions and five having session one only. Seven patients (14%) had no sessions because they declined, were discharged home early or deteriorated clinically. Median duration of sessions one to three was 35, 30 and 30 minutes respectively. 40 patients (90%) received a tablet computer, and 27 (61%) were given a DVD and patient booklet to keep.

283 (84%) staff in the two critical care units took the online training course, surpassing the target rate of 80%, with 277 (98%) passing the end of course assessment. Ten POPPI nurses (100%) completed the three-day training course and the additional day for feedback and assessment. Nine (90%) passed the assessment the first time and one on the second attempt.

For peer review only - http://bmjopen.bmj.com/site/about/guidelines.xhtml

Once the intervention was embedded, fifteen patients complete a satisfaction questionnaire. Satisfaction with stress support sessions was rated 4 or 5 (on a scale of 0-5) by 14 patients (93%). Fifteen (100%) patients gave ratings of 4 or 5 for nurse understanding and normalising fears. Twelve (80%) rated 4 or 5 for the number and duration of sessions. Ten out of 14 (71%) rated satisfaction with contents of the tablet app at 4 or 5, and 10 out of 15 (67%) said they gained useful ideas for coping strategies. Scores on patient stress thermometers (0-10) fell by median 3 points (interquartile range 1 to 5 points) from the start of session one to the end of session three among the 25 patients who received all three sessions.

Staff rated the online training course at 4 or 5 for being stimulating (189/260, 73%), useful (223/260, 86%) and well-designed (216/257, 84%). Ten (100%) POPPI nurses gave the face-to-face course ratings of 4/5 (0-5) for being stimulating, useful, well-conducted and motivating, with 9 (90%) giving those ratings for its relevance to their new role. There was a large increase in nurse self-efficacy, from 30% (21/7) gaining scores of 4/5 pre-course, to 73% (51/70) gaining 4/5s post-course. The same number/proportion of 4/5 scores were maintained at follow-up. For nurse learning, 87% (69/79) of post-course scores were 4/5s.

Quantitative feasibility and acceptability data are presented more fully in Table 3. Qualitative data on the three elements are summarised in table three, but described in more detail below.

Table 3: Feasibility, acceptability and refinement of the three elements of the POPPI psychological intervention

For peer review only - http://bmjopen.bmj.com/site/about/guidelines.xhtml

Elements of intervention	Content/ delivery of element	Feasibility and acceptability indicators – quantitative and qualitative	Feasibility and acceptability results	Refinement of the intervention post-feasibility study
Element one: Creating a therapeutic environment in critical care	Content of online training course	Training course ratings by all staff (% giving 4 or 5 out of 5)<br><br>Favourite parts of course – all staff<br><br>Nurse qualitative feedback (see p14)	Stimulating 73%; Useful 86%; Well-designed 84%; Right length 80% (n=260, but missing data for some items)<br><br>Factual information 42%; Patient stories 35%; Communication videos 13%; Tests 10% (n=260)<br><br>Staff positive, suggested minor improvements	Online training course shortened and made more visually appealing, with more practical advice on reducing stressors in critical care units and clearer presentation of key messages
	Delivery of online training and creating a therapeutic environment	Staff taking course (target: 80%)<br><br>Staff passing final test (score >80%)<br><br>Staff learning scores (% with 4 or 5 out of 5)<br><br>Nurse qualitative feedback (p15)	283 (84%)<br><br>277 (98%)<br><br>74% (n=259)<br><br>POPPI nurses lacked time, due to workload, to support staff in creating therapeutic environment	Provision of training, display materials and slide-sets for seminars/workshops, for local education teams to support and motivate staff in creating a therapeutic environment
Element two: Three stress support sessions for patients screened as acutely stressed	Content of screening	Previously validated[22]		
	Delivery of screening	Consenting patients screened<br>Screened as acutely stressed	127 (100%)<br><br>51 (40%)	
	Content of stress support sessions	Median (IQR) difference in patient stress thermometer scores (0 - 10)<br><br>Patient satisfaction with stress support sessions (% giving ratings of 4 or 5 out of 5)<br><br>Patient qualitative feedback (p15)<br><br>Nurse qualitative feedback (p15)	Median difference from start session 1 to end session 3 was -3.0 (-5.0, -1.0) (n=25 patients who had 3 sessions)<br><br>Overall 93%; Helped express fears 93%; Nurse understanding 100%; Nurse normalised fears 100%; Fewer stressful thoughts 87%; fewer stressful feelings 80%; number/duration of sessions 80% (n=15, missing data some items)<br><br>Stress support from nurses was very helpful<br><br>Rewarding, but challenging to explain to patients	Content of stress support sessions clarified for POPPI nurses by reorganising sessions from five components each, into three common components in all sessions and three individual components per session. Manual became more tightly focused on stress support sessions (rather than the whole intervention) with clearer signposting to and between sections
	Delivery of stress support sessions	How stress support sessions patients had<br><br>Median duration of sessions (minutes = m)<br><br>Nurse qualitative feedback (p15)	25 (49%) had three sessions; 14 (28%) had two sessions; 5 (10%) one session; 7 (14%) had none<br><br>Session one: 35m, Session 2: 30m, Session 3: 30m<br><br>Hard to fit sessions in with ordinary duties, especially if patients postponed. Patients missed session three if they were discharged home early	Ensure buy-in and support for POPPI nurses from clinical, education and research staff from the start, making it a team effort.<br>If hospital discharge is near, sessions two and three can and should be delivered together

For peer review only - http://bmjopen.bmj.com/site/about/guidelines.xhtml

	Content of POPPI nurse face-to-face training course - three days and a follow-up/assessment day	Nurse feedback – post-course questionnaire (% rating 4/5 out of 5)<br><br>Nurse self-efficacy (in delivering psychological support) – pre-/post-course and follow-up questionnaires. Average % scoring 4/5 out of 5 on 7 items<br><br>Nurse course learning – an 8-item post-course knowledge questionnaire - % nurses scoring 4/5 out of 5<br><br>Trainer assessment of nurse competence using six-item checklist (scores 0-12; pass =6)<br><br>Nurse feedback from focus groups (p15)	Stimulating 100%; Useful 100%; Relevant 90%<br>Well conducted 100%; Motivating 100% (n=10)<br><br>Big increase in self-efficacy from pre- to post-course; maintained at follow-up. Average 30% scoring 4/5s across all items pre-course; average 73% scoring 4/5s post-course; average 73% with 4/5s at follow-up<br><br>Average 87% scoring 4/5s on knowledge of: Acute stress; screening; aims of stress support sessions, normalising, psycho-education, communication style, stressful thinking, checking out fears, coping<br><br>100% passed (9 on first attempt, 1 on second)<br>Median (IQR) score of passes 9 (9, 10)<br><br>Course highly valued but tiring. Skills practice stressful. Assessment on follow-up day stressful	To reduce burden and increase self-efficacy, the three-day course became more focused on stress support sessions, particularly session two, seen as the most difficult session to deliver. More emphasis on skills practice, with actors (not fellow trainees) playing patients. Wider spectrum of patient scenarios used.
	Delivery of three-day training course	Number of required trainees attending	10 (100%)*	Some modules dropped from course; pre-course theory booklet provided
	Content: Debriefing support by trainers	Nurse feedback (qualitative, see p 15)	Debriefing calls useful for reflection, confidence<br>Assessment should be part of ongoing support	Assessment to be one-to-one confirmation of skills, as part of ongoing debriefing and support
	Delivery: Debriefing and support	Nurse feedback (qualitative, see p15)	Nurse debriefing and support should start earlier	First debriefing call after first POPPI patient
Element three: Relaxation and recovery programme on App, DVD and booklet (music, relaxation, meditation, patient recovery videos, self-help information)	Content of relaxation and recovery programme	Patient satisfaction with content of programme - % rating 4 or 5 out of 5<br><br>Nurse-reported patient feedback (qualitative, see p15)	Content on tablet computer App 71%; Useful post-ICU coping ideas 67% (15 patients, but some missing data)<br><br>Varied preferences; relaxation, meditation, nature sounds, patient stories or calming music. Some disappointed not to find classical music on DVD.	Content and design of the relaxation App were improved. Balance of contents of DVD improved, and classical music tracks were added. Layout and readability of the patient booklet were improved.
	Delivery of relaxation and recovery programme	Patients receiving tablet in session one<br><br>Patients receiving DVD or booklet<br><br>Nurse-reported patient feedback (qualitative, p15)	40 (90%)<br><br>27 (61%)<br><br>Some liked tablet, others found it hard to use , some preferred DVD or patient booklet	Usability of the relaxation App was improved, to make it easier for the less dextrous. Higher spec tablets, including better touch sensitivity, identified for future<br><br>DVD and booklet to be provided in session two, so more patients would receive them

**Initially three nurses each at two sites were trained as planned, but four additional nurses were trained at one site due to personnel issues*

Nurse gave positive feedback about the content of element one, “creating a therapeutic environment in the critical care unit” with comments that the online training course had raised awareness and changed staff

For peer review only - http://bmjopen.bmj.com/site/about/guidelines.xhtml

thinking. However design improvements, more practical advice and summaries of key messages in the course were suggested. Delivery of this element was challenging, as POPPI nurses had insufficient time to support and educate other staff in creating a therapeutic environment. It was suggested that this responsibility should be shared more widely by the critical care team, and that further educational and promotional materials were needed.

Nurse feedback on content of the second element was that stress support sessions were welcomed by patients and rewarding for nurses. Challenges included ensuring patients understood the sessions and knowing how to handle very serious issues that might be raised. Delivery of stress support sessions could be difficult to fit into heavy clinical workloads, especially if patients postponed sessions due to fatigue or sickness. Comments on the three-day training course included “best course ever” and “feel privileged” to be part of it. However, nurses found the course hard work and tiring. They suggested that psychological theory should be delivered to be read in advance, rather than presented during the course, and that skills practice should involve actors (rather than fellow trainees) to play patients. They found the POPPI nurse training manual invaluable, but suggested some re-structuring in the presentation of stress support sessions, with better linkage to appendices.
All nurses found the skills development assessment on the additional training day stressful, and suggested that assessment could be carried out in one-to-one debriefing and support sessions from trainers. Most said they received the right amount of support, and that debriefing was useful for reflection and building greater confidence.

Patient feedback on the stress support sessions was overwhelmingly positive, and included “every hospital should have it”, “it seemed a life saver”, “proves that I am normal but under the influence of morphine” and “I hope that POPPI will eventually be used in all ICUs. Thank you for the support.” Criticisms included one patient’s wish that the nurse had made improvements to the unit in response to his concerns, and another’s opinion that more than three stress support sessions were needed.

Patients also responded positively to the relaxation and recovery programme. Some patients preferred patient stories, some relaxation exercises, some meditation and some calming music. Some found the tablet computers confusing and difficult to use, due to unfamiliarity with the technology, or reduced dexterity. Unfortunately the tablets used in the feasibility pilot study did not have good touch-sensitivity. Some patients were disappointed that the DVDs did not include the calming music (due to copyright issues).

Refinement of the complex intervention

We refined the complex intervention based on feasibility data. We made POPPI online training shorter and clearer, and included more practical advice and emphasis on key messages. We created slide-sets and display materials for units to reinforce the key messages. The wider critical care team should be involved to promote education and behaviour change in creating a therapeutic environment, so that POPPI nurses can focus on delivering stress support sessions to patients. Accordingly, the three-day POPPI nurse course is more tightly

For peer review only - http://bmjopen.bmj.com/site/about/guidelines.xhtml

focused on the stress support sessions, with more emphasis on skills practice with actors playing patients. Some modules are provided to POPPI nurses in advance, to reduce the intensity of the course. POPPI nurse assessment is carried out less formally as part of one-to-one debriefing and support sessions with trainers. The content, design and usability of the relaxation App has been improved, and tablets with better touch sensitivity identified. The DVD and patient booklet are given to patients in session two rather than session three so that more patients receive that element of the intervention. Their appearance and contents have also been improved. See last column, table one, for the detail of refinements made to each element.

Trial procedures feasibility study

There was a two-month recruitment period. Of 160 eligible patients in the two sites, 86 (53.8%) provided consent to take part (see figure three). Of these, nine (10.5%) had died by 5 months, but 62 (80.5%) completed the 5-month follow-up questionnaire. Overall completeness of the primary outcome measure (the PSS-SR[31]) was very good. For a total of 1054 fields, only 24 (2.3%) had missing data.

DISCUSSION

We undertook the first and second stages of the MRC's updated framework[26] to develop and test the feasibility of a complex intervention to reduce patients' acute stress in critical care, and prevent long-term psychological morbidity. The intervention has three elements: Creating a therapeutic environment; three stress support sessions delivered by selected, trained nurses to acutely stressed patients, and a relaxation and recovery programme. One feasibility study showed that content and delivery of the complex intervention were feasible and acceptable for patients and staff, and led to a refined version of the intervention ready for evaluation in a cluster-randomised clinical trial (cRCT). The other feasibility study tested procedures for a cRCT. By the end of the development and feasibility period, the team devised and created an online training course for critical care staff; a relaxation and recovery App, DVD and booklet for patients; and a POPPI nurse training package, including manual of the stress support sessions, three-day face-to-face course with training folder, and a debriefing and support programme.

Strengths of this work were that it built on a doctoral research programme on the psychological impact of critical care,[1] and post-doctoral work to develop a psychological assessment tool and to review and model potential psychological interventions. The POPPI intervention was developed using an up-to-date MRC framework[26] by a clinical research team including patient survivors, overseen by an advisory group of psychological, nursing and education experts .

The feasibility and acceptability of content and delivery of the complex intervention were tested with both staff and patients using mixed methods research. More than 200 patients were involved in the two feasibility studies. As well as specific nurses being trained, more than 280 staff were involved in an online training course. Limitations were that the intervention was piloted in only two sites and there was limited patient feedback on the intervention. No efficacy data were collected, given these were feasibility, not evaluation studies.

For peer review only - http://bmjopen.bmj.com/site/about/guidelines.xhtml

The POPPI intervention addresses evidence that critical care patients suffer acute and long-term stress.[8-10] Despite official and clinical recognition[13, 15] that effective interventions to reduce critical care stress are needed, little has been done to date. Studies of early interventions such as music, relaxation and psychological support in critical care showed promise.[25, 49] POPPI is the first complex psychological intervention designed to be delivered at an early stage of the critical care pathway, to prevent PTSD and other long-term distress.

Conclusion

We developed, feasibility-tested and refined a nurse-led, preventative psychological intervention ready for evaluation in a cluster-randomised clinical trial.

Supplementary material: Click here to view

Contributors

Early conceptual and development work for the POPPI intervention was carried out by DW, JWei, DHo, MM, DS, JW, NA, MH, CW and CB at University College London Hospitals. Further development of the intervention and design of the feasibility phase of the POPPI study were carried out with KR, SH, DHa, PM, ARB, DD'A and VB. DW drafted the initial manuscript with input from KR. All authors revised the manuscript critically and gave their approval of the final version. DW is the guarantor.

Acknowledgements

Daniel Freeman (chair of the education and psychology advisory group), Dane Goodsman (clinical education), Rob Beer (online training design), Daniel Zanitchkhah (app design), Derek Tutssel (video directing), Lih-Mei Liao, Wendy Harris, Steve Saunders, Ruth Canter, Rahi Jahan, Blair McLennan, Emma Wood. David Aaronovitch, Sheila Richards, Peter Cross, Rajadurai Sunderalingham, Kate Baden-Fuller, Caroline Knight, and other members of the UCLH patients and relatives advisory group.

"POPPI" nurses and site PIs:

University College Hospital, London: Emma Davies, Emma Thompsett, Rosalind Edwards, David Brealey, Magda Rocha, Jung Ryu, Georgia Bercades. Watford General Hospital: XioBei Zhao; Jennie Haydock, Kalpana Giri Ghimire, Lillian Norris, James Cunningham, Sarah- Jane Turner, Tracey Temple

Medway Maritime Hospital: Catherine Plowright, Claire Pegg, James Cullinane

Bristol Royal Infirmary: Katie Sweet, Sanjoy Shah, Lisa Grimmer.

Funding

This project was funded by the National Institute for Health Research (NIHR) Health Services and Delivery Research (HS&DR) Programme (Project: 12/64/124). DW also received funding from the University College London/ University College London Hospitals NIHR Biomedical Research Centre. The views and opinions expressed in this paper are those of the authors and do not necessarily reflect those of the HS&DR, NIHR, NHS

For peer review only - http://bmjopen.bmj.com/site/about/guidelines.xhtml

or the Department of Health. The funding body had no role in the design of the study and collection, analysis, and interpretation of data and in writing the manuscript.

Ethics

The two pilot studies were granted favourable ethical opinion by the NRES Committee South Central - Oxford B research ethics committee on 23 April 2014 (reference: 14/SC/0149), were managed by the ICNARC CTU and prospectively registered on the ISRCTN Registry (ISRCTN61088114).

Transparency Declaration

The lead author affirms that the manuscript is an honest, accurate transparent account of the study being reported, that no important aspects of the study have been omitted, and that any discrepancies from the study as planned, have been explained.

Copyright

The Corresponding Author has the right to grant on behalf of all authors and does grant on behalf of all authors, a worldwide licence to the Publishers and its licensees in perpetuity, in all forms, formats and media (whether known now or created in the future), to i) publish, reproduce, distribute, display and store the Contribution, ii) translate the Contribution into other languages, create adaptations, reprints, include within collections and create summaries, extracts and/or, abstracts of the Contribution, iii) create any other derivative work(s) based on the Contribution, iv) to exploit all subsidiary rights in the Contribution, v) the inclusion of electronic links from the Contribution to third party material where-ever it may be located; and, vi) licence any third party to do any or all of the above. We attest that we have obtained appropriate permissions and paid any required fees for use of copyright protected materials.

Competing interests

All authors have completed the ICMJE uniform disclosure form at www.icmje.org/coi_disclosure.pdf and declare: no support from any organisation for the submitted work; no financial relationships with any organisations that might have an interest in the submitted work in the previous three years; no other relationships or activities that could appear to have influenced the submitted work."

No additional data available

For peer review only - http://bmjopen.bmj.com/site/about/guidelines.xhtml

For peer review only

References

1. Wade DM. Doctoral thesis: Prevalence and predictors of psychological morbidity and quality of life after discharge from Intensive Care. University College London, 2010.
2. Needham DM, Davidson J, Cohen H, et al. Improving long-term outcomes after discharge from intensive care unit: report from a stakeholders' conference. Critical care medicine 2012;**40**(2):502-9.
3. Puntillo KA, Arai S, Cohen NH, et al. Symptoms experienced by intensive care unit patients at high risk of dying. Critical care medicine 2010;**38**(11):2155-60.
4. Novaes MA, Knobel E, Bork AM, et al. Stressors in ICU: perception of the patient, relatives and health care team. Intensive care medicine 1999;**25**(12):1421-6.
5. Alonso-Ovies A, Heras La Calle G. ICU: a branch of hell? Intensive care medicine 2016;**42**(4):591-2.
6. Nelson JE. The symptom burden of chronic critical illness. Critical care medicine 2004;**32**.
7. Jones C, Griffiths RD, Humphris G, et al. Memory, delusions, and the development of acute posttraumatic stress disorder-related symptoms after intensive care. Critical care medicine 2001;**29**(3):573-80.
8. Davydow DS, Gifford JM, Desai SV, et al. Depression in general intensive care unit survivors: a systematic review. Intensive care medicine 2009;**35**(5):796-809.
9. Davydow DS, Gifford JM, Desai SV, et al. Posttraumatic stress disorder in general intensive care unit survivors: a systematic review. General hospital psychiatry 2008;**30**(5):421-34.
10. Wade DM, Howell DC, Weinman JA, et al. Investigating risk factors for psychological morbidity three months after intensive care: a prospective cohort study. Critical care (London, England) 2012;**16**(5):R192.
11. Huang M, Parker AM, Bienvenu OJ, et al. Psychiatric Symptoms in Acute Respiratory Distress Syndrome Survivors: A 1-Year National Multicenter Study. Critical care medicine 2016.
12. Bienvenu OJ, Colantuoni E, Mendez-Tellez PA, et al. Cooccurrence of and remission from general anxiety, depression, and posttraumatic stress disorder symptoms after acute lung injury: a 2-year longitudinal study. Critical care medicine 2015;**43**(3):642-53.
13. National Institute of Health and Care Excellence (NICE) clinical guideline 83 – Rehabilitation after critical illness in adults. London: NICE, 2009.
14 National Institute of Health and Care Excellence (NICE) quality standard 158 - Rehabilitation after critical illness in adults: NICE, 2017
15. Hatch R, McKechnie S, Griffiths J. Psychological intervention to prevent ICU-related PTSD: who, when and for how long? Critical care (London, England) 2011;**15**(2):141.
16. Cuthbertson BH, Rattray J, Campbell MK, et al. The PRaCTICaL study of nurse led, intensive care follow-up programmes for improving long term outcomes from critical illness: a pragmatic randomised controlled trial. BMJ (Clinical research ed) 2009;**339**:b3723.
17. Mehlhorn J, Freytag A, Schmidt K, et al. Rehabilitation interventions for postintensive care syndrome: a systematic review. Critical care medicine 2014;**42**(5):1263-71.
18. Jensen JF, Thomsen T, Overgaard D, et al. Impact of follow-up consultations for ICU survivors on post-ICU syndrome: a systematic review and meta-analysis. Intensive care medicine 2015;**41**(5):763-75.
19. Davydow DS, Zatzick D, Hough CL, et al. In-hospital acute stress symptoms are associated with impairment in cognition 1 year after intensive care unit admission. Annals of the American Thoracic Society 2013;**10**(5):450-7.
20. Nikayin S, Rabiee A, Hashem MD, et al. Anxiety symptoms in survivors of critical illness: a systematic review and meta-analysis. General hospital psychiatry 2016;**43**:23-29.
21. Rabiee A, Nikayin S, Hashem MD, et al. Depressive Symptoms After Critical Illness: A Systematic Review and Meta-Analysis. Critical care medicine 2016;**44**(9):1744-53.
22. Jones C, Backman C, Capuzzo M, et al. Precipitants of post-traumatic stress disorder following intensive care: a hypothesis generating study of diversity in care. Intensive care medicine 2007;**33**(6):978-85.
23. Schelling G. Effects of stress hormones on traumatic memory formation and the development of posttraumatic stress disorder in critically ill patients. Neurobiology of learning and memory 2002;**78**(3):596-609.
24. Peris A, Bonizzoli M, Iozzelli D, et al. Early intra-intensive care unit psychological intervention promotes recovery from post traumatic stress disorders, anxiety and depression symptoms in critically ill patients. Critical care (London, England) 2011;**15**(1):R41.
25. Wade DF, Moon Z, Windgassen SS, et al. Non-pharmacological interventions to reduce ICU-related psychological distress: a systematic review. Minerva anestesiologica 2016;**82**(4):465-78.
26. Craig P, Dieppe P, Macintyre S, et al. Developing and evaluating complex interventions: the new Medical Research Council guidance. BMJ (Clinical research ed) 2008;**337**:a1655.
27. Wade D, Hardy R, Howell D, et al. Identifying clinical and acute psychological risk factors for PTSD after critical care: a systematic review. Minerva anestesiologica 2013;**79**(8):944-63.
28. Sessler CN, Gosnell MS, Grap MJ, et al. The Richmond Agitation-Sedation Scale: validity and reliability in adult intensive care unit patients. American journal of respiratory and critical care medicine 2002;**166**(10):1338-44.

For peer review only - http://bmjopen.bmj.com/site/about/guidelines.xhtml

29. Wade DM, Hankins M, Smyth DA, et al. Detecting acute distress and risk of future psychological morbidity in critically ill patients: validation of the intensive care psychological assessment tool. Critical care (London, England) 2014;**18**(5):519.
30. Kirkpatrick DL, Kirkpatrick JD. *Implementing the Four Levels*. San Francisco, CA: Berrett-Koehler Publishers, 2007.
31. Foa EB, Cashman L, Jaycox L, et al. The validation of a self-report measure of posttraumatic stress disorder: the Posttraumatic Diagnostic Scale. . Psychological Assessment 1997;**9**:445-51.
32. Irwin M, Artin KH, Oxman MN. Screening for depression in the older adult: criterion validity of the 10-item Center for Epidemiological Studies Depression Scale (CES-D). Archives of internal medicine 1999;**159**(15):1701-4.
33. Parker AM, Sricharoenchai T, Raparla S, et al. Posttraumatic stress disorder in critical illness survivors: a metaanalysis. Critical care medicine 2015;**43**(5):1121-9.
34. Wade D, Moon Z, Windgassen S, et al. Nonpharmacological interventions to reduce short-term or long-term psychological stress in ICU patients: a systematic review. Critical Care 2015;**19**(Suppl 1):P556-P56.
35. Wade DM. MSc thesis: The psychology of weaning from mechanical ventilation: a mixed methods study of mood, control, support and communication in the intensive care unit. University of London, 2006.
36. Wade DM. Portfolio of competence, stage II health psychology: The British Psychological Society, 2011.
37. Wade DM, Brewin CR, Howell DC, et al. Intrusive memories of hallucinations and delusions in traumatized intensive care patients: An interview study. British journal of health psychology 2014.
38. Lazarus RS, Folkman S. *Stress, appraisal, and coping.* New York, NY: Springer Publishing Company, 1984.
39. Brewin CR. Re-experiencing traumatic events in PTSD: new avenues in research on intrusive memories and flashbacks. Eur J Psychotraumatol 2015;**6**:27180.
40. Hobfoll S, Watson P, Bell C, et al. Five Essential Elements of Immediate and Mid-Term Mass Trauma Intervention: Empirical Evidence. FOCUS 2009;**7**(2):221-42.
41. van der Gaag M, Valmaggia LR, Smit F. The effects of individually tailored formulation-based cognitive behavioural therapy in auditory hallucinations and delusions: A meta-analysis. Schizophrenia Research 2014; **156** (1): 30-37
42. Fowler D, Garety P, Kuipers E. *Cognitive behaviour therapy for psychosis: theory and practice.* Chichester: John Wiley and Sons, 1995.
43. Kolb DA. *Experiential learning: experience as the source of learning and development*. 1 ed. Englewood Cliffs, NJ: Prentice Hall, 1984.
44. Michie S, van Stralen MM, West R. The behaviour change wheel: a new method for characterising and designing behaviour change interventions. Implementation science 2011;**6**:42.
45. Silverman J, Kurtz S, Draper J. *Skills for Communicating with Patients*. Oxford: Radcliffe Publishing, 2013.
46. Bowers L, Brennan G, Winship G, et al. How expert nurses communicate with acutely psychotic patients. Mental Health Practice 2010;**13**(7):24-26.
47. Stoltenberg CD, McNeill B, Delworth U. *IDM supervision: An integrated developmental model for supervising counselors and therapists.* San Francisco: Jossey-Bass, 1998.
48. Bullock I, Davis M, Lockey A, et al. *Pocket Guide to Teaching for Clinical Instructors*. 3 ed. West Sussex: John Wiley & Sons Ltd., 2016.
49. Papathanassoglou ED. Psychological support and outcomes for ICU patients. Nursing in critical care 2010;**15**(3):118-28.

Figure legends

Figure one: Use of stages one and two of the Medical Research Council model for developing and evaluating complex interventions

Figure two: Patient flow in the mixed-methods intervention feasibility study

Figure three: Consort diagram for the trial procedures feasibility study

For peer review only - http://bmjopen.bmj.com/site/about/guidelines.xhtml

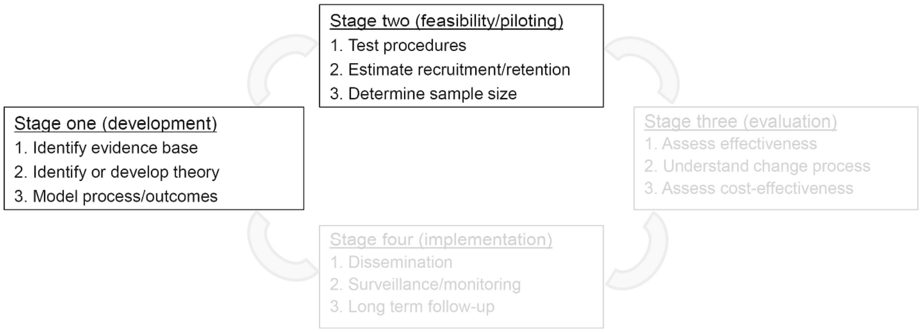

254x190mm (300 x 300 DPI)

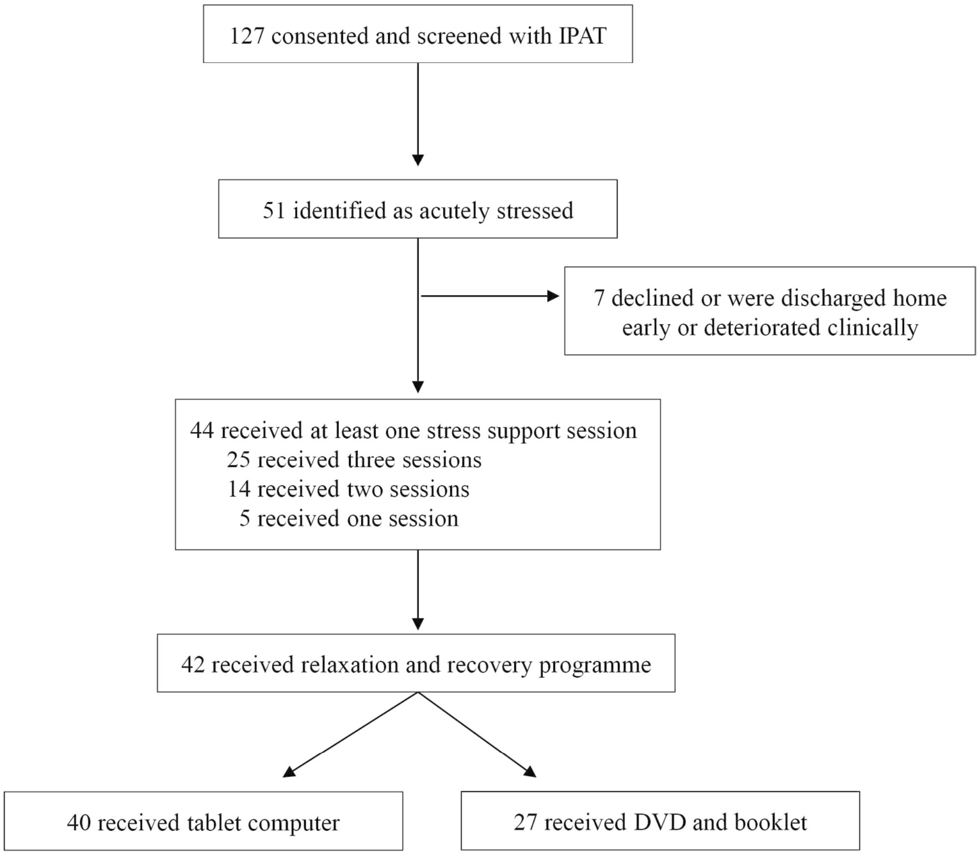

172x153mm (300 x 300 DPI)

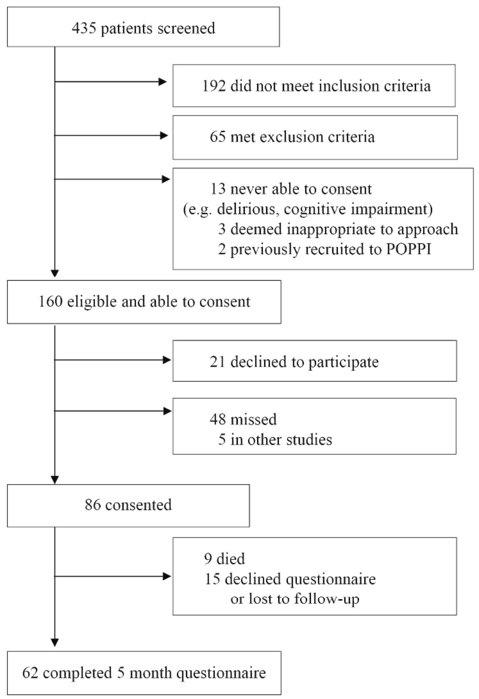

254x190mm (300 x 300 DPI)

Electronic supplement one

Title: Provision of psychological support to people in intensive care (POPPI): Developing and testing a nurse-led complex intervention

This supplement presents further details of the final post-feasibility version of the three-element POPPI complex intervention. This version is being evaluated in the POPPI RCT.

Element one: Creating a therapeutic environment

Online training course
A key part of element one is the POPPI online training course: Key skills in Psychological Care. Training is undertaken by all clinical critical care staff to aid the creation of a calm, less stressful environment by reducing stressors in the environment and using good communication with patients. The course takes 30 minutes and comprises the following five sections:

1 Understanding the stresses of intensive care patients
2 Reducing stress and fear in patients
3 Communicating with distressed patients
4 Inspiring patients with confidence and hope
5 Summary and assessment

The course has a balance of concise, readable text with graphics and other visual or audio aids, and test-yourself questions at the end of each section (with informative feedback). Video clips include former patients talking about their experiences of critical care and nurses modelling good communication strategies with patients. Staff who pass the assessment (a score of 80% or more) receive a certificate.

Element two: Three stress support sessions

Three, one-to-one, stress support sessions lasting approximately 30 minutes each, are delivered to a patient by the same trained POPPI nurse, starting when patients are awake and alert, either in the critical care unit or following discharge to the hospital ward. The three sessions should be delivered within a week (for the pragmatic reason that many patients may be discharged from hospital around that time).

All stress support sessions contain three common components – starting the session; building rapport and finishing the session - and three individual components per session. Starting a session includes introductions, outlining aims, measuring stress and explaining confidentiality, note-taking and risk-reporting. Building rapport involves focusing throughout all the sessions on creating a relationship with the patient based on openness, honesty, warmth and listening. Finishing a session involves summarising, getting patient feedback, agreeing practice, re-measuring stress, agreeing wording for notes and arranging the next session.

The three individual components nurses deliver in session one are: Normalise reactions (discuss common psychological reactions in critical care and possible reasons); Encourage communication (help patients open up about worries and concerns), and Teach coping strategies, e.g. patients gathering more information and practising relaxation (acronym NET). The second session comprises: Stress reactions (encourage further expression of worries and notice stressful thoughts); Explain stressful thinking (show how unrealistic fears can create extra stress), and Teach 'check out my fear', a technique to understand how accurate fears are (SET). The third session includes: Summarise and review (key messages from sessions and persisting issues); Action plan (personal planning to build on sessions and cope with challenges ahead) and Future Expectations (reinforce progress made and encourage realistic optimism for psychological and physical recovery (SAFE).

For peer review only - http://bmjopen.bmj.com/site/about/guidelines.xhtml

Education package to deliver element two

The education package to train POPPI nurses to deliver element two includes a central three-day face-to-face training course with associated materials; a local debriefing and support visit, including a skills development assessment, and ongoing debriefing and support phone calls from trainers.

The course involves pre-course study (a psychological theory booklet, a manual for the three stress support sessions and access to the POPPI online training course) and a three-day programme, as follows:

Day one

- Understanding critical care patients' stress
- Learning the skills needed to deliver stress support sessions
- Overview of the stress support sessions
- Observing and practising session one

Day two

- More about unusual experiences in critical care
- Learning further stress support skills
- Observing and practising stress support session two

Day three

- Revision of key points from stress support session two
- Observing and practising stress support session three
- Using the patient booklet to create personal action plans
- Debriefing and support arrangements

Course materials include training folder with day 1-3 course handouts, word and visual summaries of each session, checklists and reflective notes for each session, stress thermometers, copies of patient booklet and DVD.

Debriefing and support

The first debriefing and support call should occur soon after (or during, if nurse requests) the three stress support sessions nurses deliver to their first POPPI patient. Subsequent calls are every two months, or on nurse's request. The focus of these calls is on enhancing nurses' skills and discussing patient cases. Emails can be exchanged between nurses and trainers about issues arising from stress support sessions at any time. Monthly teleconferences with POPPI nurses are facilitated by a trainer to encourage peer support.

A debriefing and support visit is made by an allocated trainer to offer the three POPPI nurses at one site further support, both individually and in a group. Later in the visit a skills development assessment is carried out for each nurse. Using a scenario, the nurse delivers session 2 to the trainer who plays the role of the patient. The trainer rates the nurse's competence using a checklist. The pass mark is 8/16. Nurses are required to achieve competence to continue delivering stress support sessions to further patients.

Element Three: Relaxation and recovery programme

In addition to stress support sessions, patients receive a "relaxation and recovery" programme (using a tablet computer App between sessions, and a DVD and patient booklet to take home after hospital) to help them to sleep and cope better with stress. Former intensive care patients' recovery stories are included to help normalise ICU experiences and reactions, and encourage hope and optimism for recovery. Contents also include audio or video files of safe place visualisation, muscle relaxation and breathing exercises; a body scan and other mindfulness meditations; relaxing classical music from Bach to Vivaldi, calming modern ambient music; and restful nature sounds and scenes.

For peer review only

The programme was first developed as an application for tablet computers. The design of the App includes a green nature-scene background and large coloured buttons for patients to use for easy navigation between different sections. The tablet computers should be checked for approval by hospital infection control teams. DVDs include a shorter selection of relaxation exercises and music from the App, due to restricted space. However they include longer versions of the patient recovery stories, assuming longer concentration spans as patients get closer to hospital discharge.

The "Getting well, staying well" booklet provides patients with information on making a good psychological recovery after a critical care stay. It builds on the support patients received during the stress support sessions, focusing on well-being and coping strategies. Contents include information about what to expect in the early days (leaving critical care, returning home; emotions after critical care, relationships), tips for psychological wellbeing; advice on coping with difficulties (worries, panic, low mood, memories); information on sources of psychological support; further information for family and friends and a personal action plan to promote well-being.

For peer review only

Electronic supplement two (expanded version of table one): Development of an intervention to reduce critical care patients' stress: from inception to feasibility testing

**MRC model – steps and stages**	**Preparatory phase 2006-11** MSc/PhD/ health psychology professional portfolio*	**Early modelling phase 2011-13** Post-doc; POPPI grant application †	**Feasibility and refinement phase 2013-2015** Grant-funded work‡
Stage one, step one Identify the evidence base	Identified systematic reviews and studies of psychological outcomes, risk factors and conducted new systematic reviews of PTSD, depression, anxiety post-ICU[1]<br><br>Literature review of ICU stressors, reactions, memories, stress measurement and stress reduction interventions[1]	Updated evidence base to 2013<br>Identified new systematic reviews and studies of ICU psychological outcomes, risk factors, stress, stress measurement, stress reduction interventions[10,19,27,24]<br><br>Began systematic review of psychosocial interventions (psychological, music, mind-body therapies) to reduce ICU-related stress[25,34]	Updated evidence base of studies and systematic reviews[12]<br><br>Completed systematic review of interventions to reduce critical care stress[34]
Stage one, step two Develop/identify theory	Investigated acute stress in ICU and psychological outcomes:<br>1. Quant study (n=40) MSc[35]<br>2. Quant study incl. early ICU stress scale validation data (n=157)[10]<br>3. Qual study (n=7) MSc[35]<br>4. Qual study (n=17)[37]<br>5. Qualitative study: Health-talk online (n=40)<br><br>Developed a model of ICU stress, and identified relevant theories of stress, health and coping[1,38,39]<br><br>Explored nurse perceptions of patients' psychological needs and nurses' ability to attend to them: Content analysis of 5 focus groups at GSTT NHS trust with 125 nurses[36]<br><br>Identified communication[45] and adult learning theories[30, 43] to inform development of training to enhance ICU nurse psychological skills	Formal ICU PPI group set up at UCH; regular meetings to brainstorm and generate research ideas about patients' psychological needs and how they could be met<br><br>Improved conceptualisation and measurement of acute ICU stress: Development and validation of the Intensive care psychological assessment tool[29 in] response to NICE CG83 guideline[83]<br><br>Identified CBT models (general[38], psychosis[42] and PTSD[39, 40]) relevant to acute stress in ICU patients (through intervention mapping methodology and consultation with experts ET, CB, DF)<br><br>Identified specific stress/coping theories to inform use of relaxation and music; and social comparison theory to inform use of patient stories in a relaxation and recovery programme	Identified COM-B behaviour change model[44] as relevant to changing ICU nurse behaviour in creating a therapeutic environment<br><br>Identified clinical supervision model for non-experts delivering psychological support[47]
Stage one, step three Model process and outcomes	Developed , piloted and evaluated a training day at GSTT to train ICU nurses in communication/psychological skills (utilising actors)[36]	Consultation with experts and discussions between UCLH, ICNARC and patient representatives, to develop the basic structure of a psychological intervention consisting of three elements: Creating a therapeutic environment; three stress support sessions; and a relaxation and recovery	Formation of Expert Psychological Advisory Group of medical educators, psychologists patients and the research team, to inform the final part of POPPI intervention development<br><br>Further development of stress support sessions by senior nurses and psychologists

For peer review only

		programme. Each element to have associated training components (online, face-to-face or training materials)<br><br>Early development of stress support sessions at UCH – iterative process with patients by DW, based on CBT theory and models, supervised by senior psychologists (CB, JW, LML)<br><br>Identification of potentially suitable materials for a relaxation/recovery program<br><br>PTSD selected as primary outcome (based on systematic reviews identified in step one – more evidence of risk factors than for other psychological outcomes)	Creating materials to deliver the complex intervention (POPPI nurse manual, checklists, notes and summaries; researching/creating content for and design of a relaxation and recovery App, a DVD and a psychological well-being booklet for patients)<br><br>Creating training courses and materials for ICU staff to deliver the POPPI intervention:<br>Online training course for all staff<br>3-day face to face course for POPPI nurses<br>Follow-up assessment day (w actors)<br>Training folder<br><br>POPPI nurse supervision briefings
Stage two Feasibility and Piloting		Designing two feasibility studies – the intervention feasibility study and the trial procedures feasibility study	Conducting the two feasibility studies – intervention feasibility study and trial procedure study<br><br>Refinement of the complex intervention based on quantitative and qualitative data from patients and staff in the intervention feasibility study

**Professional training at Kings College London and St Thomas's hospital, London and PhD at University College London/University College Hospital (UCH), London †Post-doctoral work at UCH/UCL leading to grant application with Intensive Care National Audit and Research Centre (ICNARC); ‡Grant-funded work at ICNARC and UCH*

CB Chris Brewin (author), JW John Weinman (author) LML Lih-Mei Liao, Consultant Clinical Psychologist, University College London Hospitals (UCLH)

For peer review only

TIDieR Template for Intervention Description and Replication

The TIDieR (Template for Intervention Description and Replication) Checklist*:

Information to include when describing an intervention and the location of the information

Item number	Item	Where located **	
		Primary paper (page or appendix number)	Other † (details)
	**BRIEF NAME**		
**1.**	Provide the name or a phrase that describes the intervention.	p1 (title)	______________
	**WHY**		
**2.**	Describe any rationale, theory, or goal of the elements essential to the intervention.	pp4-5 (table 1) electronic supplement (ES) 2	______________
	**WHAT**		
**3.**	Materials: Describe any physical or informational materials used in the intervention, including those provided to participants or used in intervention delivery or in training of intervention providers. Provide information on where the materials can be accessed (e.g. online appendix, URL).	pp9(table 2) -11 ES1	Not currently accessible – RCT ongoing
**4.**	Procedures: Describe each of the procedures, activities, and/or processes used in the intervention, including any enabling or support activities.	pp8-11, ES 1	______________
	**WHO PROVIDED**		
**5.**	For each category of intervention provider (e.g. psychologist, nursing assistant), describe their expertise, background and any specific training given.	pp9(table 2) -11 ES 2	
	**HOW**		
**6.**	Describe the modes of delivery (e.g. face-to-face or by some other mechanism, such as internet or telephone) of the intervention and whether it was provided individually or in a group.	pp9-11	______________
	**WHERE**		

TIDieR checklist

**7.**	Describe the type(s) of location(s) where the intervention occurred, including any necessary infrastructure or relevant features.	p2, pp6-7	______________
	**WHEN and HOW MUCH**		
**8.**	Describe the number of times the intervention was delivered and over what period of time including the number of sessions, their schedule, and their duration, intensity or dose.	pp9-10, ES1	______________
	**TAILORING**		
**9.**	If the intervention was planned to be personalised, titrated or adapted, then describe what, why, when, and how.	_____n/a_____	______________
	**MODIFICATIONS**		
**10.**‡	If the intervention was modified during the course of the study, describe the changes (what, why, when, and how).	pp 13-14 (table 3), p16	______________
	**HOW WELL**		
**11.**	Planned: If intervention adherence or fidelity was assessed, describe how and by whom, and if any strategies were used to maintain or improve fidelity, describe them.	______________	To be covered in a separate paper on a process evaluation ______________
**12.**‡	Actual: If intervention adherence or fidelity was assessed, describe the extent to which the intervention was delivered as planned.	______________	

** **Authors** - use N/A if an item is not applicable for the intervention being described. **Reviewers** – use '?' if information about the element is not reported/not sufficiently reported.

† If the information is not provided in the primary paper, give details of where this information is available. This may include locations such as a published protocol or other published papers (provide citation details) or a website (provide the URL).

‡ If completing the TIDieR checklist for a protocol, these items are not relevant to the protocol and cannot be described until the study is complete.

* We strongly recommend using this checklist in conjunction with the TIDieR guide (see *BMJ* 2014;348:g1687) which contains an explanation and elaboration for each item.

TIDieR checklist

For peer review only

* The focus of TIDieR is on reporting details of the intervention elements (and where relevant, comparison elements) of a study. Other elements and methodological features of studies are covered by other reporting statements and checklists and have not been duplicated as part of the TIDieR checklist. When a **randomised trial** is being reported, the TIDieR checklist should be used in conjunction with the CONSORT statement (see www.consort-statement.org) as an extension of **Item 5 of the CONSORT 2010 Statement.** When a **clinical trial protocol** is being reported, the TIDieR checklist should be used in conjunction with the SPIRIT statement as an extension of **Item 11 of the SPIRIT 2013 Statement** (see www.spirit-statement.org). For alternate study designs, TIDieR can be used in conjunction with the appropriate checklist for that study design (see www.equator-network.org).

For peer review only

TIDieR checklist

BMJ Open

Providing psychological support to people in critical care: development and feasibility study of a nurse-led intervention to prevent acute stress and long-term morbidity

Journal:	*BMJ Open*
Manuscript ID	bmjopen-2017-021083.R1
Article Type:	Research
Date Submitted by the Author:	09-Feb-2018

Complete List of Authors:	Wade, Dorothy; UCLH NHS Foundation Trust, Critical Care<br>Als, Nicole; UCLH NHS Foundation Trust, Critical Care<br>Bell, Vaughan; Maudesley Hospital, Psychological interventions clinic for outpatients with psychosis, Maudesley psychology centre<br>Brewin, Chris; University College London, Subdepartment of Clinical Health Psychology<br>D'Antoni, Donatella; King's College London, Institute of Pharmaceutical Science<br>Harrison, David; ICNARC,<br>Harvey, Mags; UCLH NHS Foundation Trust, Critical Care<br>Harvey, Sheila; ICNARC<br>Howell, David; UCLH NHS Foundation Trust, Critical Care<br>Mouncey, Paul; ICNARC, Clinical Trials Unit<br>Mythen, Monty; Institute of Sport Exercise and Health, 170 Tottenham Court Rd, University College London/University College London Hospitals, NIHR Biomedical Research Centre<br>Richards-Belle, Alvin; Intensive Care National Audit & Research Centre, Clinical Trials Unit<br>Smyth, Deborah; UCLH NHS Foundation Trust, Critical Care<br>Weinman, John; Kings College London, Institute of Pharmaceutical Sciences & Institute of Psychiatry<br>Welch, John; University College London Hospitals NHS Foundation Trust, Critical Care Department<br>Whitman, Chris; UCLH NHS Foundation Trust, Critical Care<br>Rowan, Kathryn; Intensive Care National Audit and Research Centre (ICNARC),

**Primary Subject Heading**:	Intensive care
Secondary Subject Heading:	Mental health
Keywords:	Adult intensive & critical care < ANAESTHETICS, Adult psychiatry < PSYCHIATRY, Depression & mood disorders < PSYCHIATRY, EDUCATION & TRAINING (see Medical Education & Training), Delirium & cognitive disorders < PSYCHIATRY

SCHOLARONE™
Manuscripts

For peer review only

Title

Providing psychological support to people in critical care: development and feasibility study of a nurse-led intervention to prevent acute stress and long-term morbidity

Authors

Dorothy Wade, Health psychologist[1]

Nicole Als, Patient representative[1]

Vaughan Bell, Clinical psychologist[2]

Chris Brewin, Professor of clinical psychology[3]

Donatella D'Antoni, Psychology research assistant[4]

David Harrison, Head statistician[5]

Margaret Harvey, Patient representative[1]

Sheila Harvey, Senior research fellow[5]

David Howell, Divisional clinical director[1]

Paul Mouncey, Senior trial manager[5]

Monty Mythen, Professor of anaesthesia and critical care[6]

Alvin Richards-Belle, Trial manager[5]

Deborah Smyth, Senior research nurse[1]

John Weinman, Professor of health psychology as applied to medicine[4]

John Welch, Consultant nurse, critical care and critical care outreach[1]

Chris Whitman, Patient representative[1]

Kathy Rowan, Director of scientific and strategic development/clinical trials unit director[5]

1. Critical care department, University College London Hospitals NHS Foundation Trust, 235 Euston Rd, London NW1 2BU
2. Psychological interventions clinic for outpatients with psychosis P079, Maudesley Psychology Centre, Maudesley Hospital, Denmark Hill, London SE58AZ
3. Research department of clinical, educational & health psychology, University College London, Gower Street, London WC1E 6BT
4. Institute of Pharmaceutical Science, King's College London, Franklin-Wilkins Building, 150 Stamford Street London SE1 9NH
5. Intensive Care National Audit & Research Centre, 24 High Holborn, London WC1V 6AZ
6. University College London/ University College London Hospitals NIHR Biomedical Research Centre c/o Institute of Sport Exercise and Health (ISEH) 170 Tottenham Court Road London, W1T 7HA

Correspondence to: Dorothy Wade, Critical Care department, T03, University College Hospital, 235 Euston Rd, London NW1 2BU. Email: Dorothy.Wade@nhs.net.

For peer review only - http://bmjopen.bmj.com/site/about/guidelines.xhtml

ABSTRACT

Objectives Adverse psychological outcomes, following stressful experiences in critical care, affect more than 50% of patients. We aimed to develop and test the feasibility of a psychological intervention to reduce acute stress in critical care and prevent future morbidity.

Design A mixed-methods intervention development study, using two stages of the Medical Research Council framework for developing and testing complex interventions. Stage one (development) involved identifying the evidence base, developing a theoretical understanding of likely processes of change, and modelling change processes and outcomes. Stage two comprised two feasibility studies.

Setting Four general adult critical care units in the UK.

Participants Stage one: Former and current critical care patients; psychology, nursing, education experts. Stage two: Current patients and staff.

Outcomes Feasibility and acceptability to staff and patients of content and delivery of a psychological intervention, measured with quantitative and qualitative data. Recruitment and retention rates for a trial.

Results Building on prior work, we standardised an intervention for nurses to provide psychological support to people in intensive care (POPPI). We devised courses and materials to train staff to create a therapeutic environment in their units, identify patients with acute stress, and deliver three stress support sessions and a relaxation and recovery programme to those patients. 127 awake, orientated patients took part in an intervention feasibility study in two hospitals. Patient and staff data indicated the psychological intervention was feasible and acceptable. Feedback was used to refine the intervention. 86 (54%) different patients entered a separate trial procedures study in two other hospitals, of which 66 (80% of surviving patients) completed psychological and health questionnaires five months after recruitment.

Conclusion The 'POPPI' psychological intervention to reduce acute patient stress in critical care and prevent future psychological morbidity was feasible and acceptable. It was refined for evaluation in a clinical trial.

Strengths and limitations of this study

Extensive prior work investigating psychological risk factors, outcomes and interventions in critical care

A rigorously-developed intervention using a well-tested framework, under expert supervision

More than 200 patients and 280 staff were involved in two separate feasibility studies

It was feasible to deliver the intervention

There was limited patient feedback on the intervention

No efficacy data were collected in the feasibility studies

For peer review only - http://bmjopen.bmj.com/site/about/guidelines.xhtml

INTRODUCTION

More than 170,000 patients are admitted to adult, general critical care units in the National Health Service (https://www.icnarc.org/DataServices/Attachments/Download/a30185e2-0e19-e711-80e6-1402ec3fcd79) in England, Wales and Northern Ireland each year. Although medical advances mean that increasing numbers of people survive, there is evidence that many critical care patients develop both acute stress and long-term psychological morbidity.[1] Critical care units are stressful places, where patients suffer symptoms such as pain, thirst, nausea, fatigue, and disorientation associated with critical illness, invasive medical procedures and side-effects of potent drugs.[2, 3] Being attached to machines, tubes, masks and other equipment in a busy, noisy ward, often without access to daylight, windows or clocks, leads to sensory disruption and sleep deprivation. Patients are often isolated, unable to communicate, but aware of other people's suffering or death.[4] Unsurprisingly, 45%-80% of critical care patients experience acute stress in the form of panic, fear, depressed mood, anger, as well as hallucinations or delusions. They may have flashbacks of frightening ICU experiences including hallucinations or delusions after leaving hospital.[5-7] Studies suggest that up to 50% of patients or more experience symptoms consistent with post-traumatic stress disorder (PTSD), anxiety or depression following critical care.[8-12]

The UK National Institute for Health and Care Excellence (NICE) guideline on rehabilitation from critical illness recommends that highly stressed patients should be identified and offered psychological support as part of a recovery plan. Quality standards have recently been implemented to strengthen these requirements.[13] Most interventions to reduce psychological morbidity have been implemented in the months following discharge from critical care and hospital. They include follow-up clinics, rehabilitation services, patient diaries and nurse-led psychological recovery sessions.[14-17] Diaries of patients' admissions, written mainly by staff and given to patients one to three months post-critical care discharge, reduced acute-onset PTSD at three months in one randomised clinical trial (RCT).[18] Training critical care survivors in mindfulness and coping skills training was found to be feasible in small studies, but in an RCT coping skills training after hospital did not reduce psychological distress at six months.[19] Overall there is a lack of clear evidence that critical care follow-up services reduce long-term distress.[15-16]

Given that patients who suffer acute stress, including hallucinations and delusions, in the critical care unit have a higher risk of future psychological morbidity,[20,21] psychological interventions commenced during critical care admissions could be effective. But due to a lack of evidence about what helps, little is currently done to alleviate stress in critical care. Sedatives or antipsychotics may be prescribed to reduce stress and agitation, but they have potentially harmful side-effects, both physical and psychological. Evidence exists that psychosocial interventions such as music therapy, massage, reflexology, relaxation and clinical psychology sessions delivered in critical care, can reduce patients' stress.[22-24] But more robust research is urgently needed.

We hypothesised that techniques from psychological interventions used in other populations, such as people with psychosis or trauma,[25-28] could be effective for patients still in the critical care unit. At an early development stage, a psychological approach used to reduce anxiety, distress from hallucinations and

For peer review only - http://bmjopen.bmj.com/site/about/guidelines.xhtml

delusions, and traumatic stress, was adapted for critical care, and used by a psychologist in one unit (electronic supplement one). As psychologists are a scarce resource in hospitals, we proposed that, given extra training, critical care nurses have the necessary experience and motivation to provide psychological support to patients. We developed the intervention using the UK's Medical Research Council framework (see figure one) to guide researchers in creating evidence-based, theoretically sound and robustly evaluated complex interventions.[29] This paper covers stage one (development) and stage two (feasibility/piloting) of the framework. Stage three (evaluation of clinical and cost-effectiveness in a cluster-randomised clinical trial) will be reported separately.

The objectives of this mixed-methods intervention development study were to: standardise a nurse-led psychological intervention to support patients in critical care; to create an associated staff education package; to test the feasibility and acceptability of the intervention; to test trial procedures, and to refine the intervention for evaluation in a trial (https://www.journalslibrary.nihr.ac.uk/programmes/hsdr/1264124/#/).

DEVELOPMENT (STAGE ONE)

Methods

Stage one of the MRC framework includes three key tasks: Identifying the evidence base for an intervention; developing a theoretical understanding of the likely processes of change; and modelling process to progressively refine the intervention (figure one). We reviewed the evidence base on critical care-related psychological risk factors, outcomes and interventions.[1,30] Two authors conducted a new systematic review of psychological interventions in critical care.[25] We also conducted primary research studies[7,31-33] to develop an understanding of the determinants of psychological distress in critical care and the likely mechanisms of change involved in reducing stress (see electronic supplement one).

The modelling process involved testing and revising of the complex intervention, by psychologists, nurses, and patients, supervised by an expert psychological advisory group (EPAG) including clinicians, an educationalist and specialists in psychological interventions for psychosis and trauma. This resulted in the nurse-led POPPI (provision of psychological support to people in intensive care) intervention, comprising three inter-connected elements: Creating a therapeutic environment for all patients; delivering stress support sessions for patients with acute stress, and provision of a relaxation and recovery programme. Acute stress was defined by scores >7 (0-20) on the Intensive care Psychological Assessment Tool (IPAT) previously validated by our group.[34] An education package was created to train staff to deliver the intervention, with help from the EPAG, educational and web designers and a medical film-maker. A patients and family advisory group was set up at the start of the development phase at University College Hospital, London. Twenty or so members contributed to every stage of intervention development and subsequently to the cRCT. The group was recruited by letters, leaflet, posters and personal contacts. It is diverse in age, gender, culture and critical care experiences, and members have played varying roles in the trial steering committee, EPAG and POPPI training team. Several members were filmed narrating their critical care experiences, for use in the nurse training courses and to be viewed by current patients as part of the relaxation and recovery programme.

For peer review only - http://bmjopen.bmj.com/site/about/guidelines.xhtml

Results

Our review of the evidence base[1,30] pointed to likely prevalence rates of 40% clinical anxiety symptoms, 30% depression, and 20% post-traumatic stress among survivors. There was increasing evidence that acute stress, as well as early intrusive or delusional memories, were important risk factors for psychological morbidity.[7,20,21] Twenty-three studies were identified In our systematic review of non-pharmacological interventions to reduce critical-care related stress,[35] with 12 showing beneficial effect, although quality and quantity of evidence was limited. However music, relaxation, visualisation and psychotherapeutic approaches showed promise as elements of a complex psychological intervention.

Our primary research to further understanding of the determinants and proposed mechanisms of change involved consulting hundreds of patients, relatives and nurses using questionnaires, interviews or focus groups.[7,31-33] Patients expressed the need for understanding and support to deal with anxiety, panic, hallucinations, delusions and flashbacks, as well as the frightening environment in the critical care unit. They wanted to trust staff, feel safe, be listened to and get more information (e.g. how drugs might cause hallucinations). They said nurses' ability to communicate with frightened or delirious patients was variable, but understood that caring for stressed, delirious patients was difficult, and suggested that training could improve communication and empower nurses to deliver one-to-one psychological support. Nurses said they were motivated to improve patients' psychological well-being and make the environment less stressful, but many thought they lacked the necessary knowledge and skills, and were frightened of "making things worse." They agreed with patients that training would help to increase staff psychological awareness and skills, along with a protocol to help staff identify patients most in need and to deliver one-to-one psychological support. The conclusion of the consultation was that all staff should be trained to reduce general stress and improve communication in critical care, but the most stressed patients would benefit from focused one-to-one psychological support (electronic supplement one).

As well as adapting techniques from psychological interventions for psychosis and trauma for the needs of critical care patients[25-28], we drew on well-established work on stress, health and coping,[36] as well as ideas to inform training health staff in new behaviours,[37] including therapeutic communication and engagement with patients, especially those who were distressed by on-going hallucinations and / or delusions[.38,39] We took into account the need for clinical supervision of non-expert staff delivering psychological support.[40] Finally, we standardised the three inter-connected elements of the complex intervention (see table one) and, guided by the EPAG educationalist, created an education package with associated materials to deliver it. The resulting POPPI intervention is described below. For more details of each element, see electronic supplement two.

In each unit three POPPI nurses were charged with ensuring that the Intensive care Psychological Assessment Tool (IPAT) was used routinely, encouraging staff behaviour change and overseeing delivery of the whole intervention. The first element of the intervention was for critical care staff to create a more therapeutic environment in their unit by improving communication with distressed patients and reducing stressors such as

For peer review only - http://bmjopen.bmj.com/site/about/guidelines.xhtml

noise, unnatural light, insomnia, pain, and psychoactive drug effects. This was primarily delivered via online training (see below for course details). The POPPI nurse role included encouraging all staff to complete online training and reinforcing key learning objectives through ongoing education and bedside teaching.

The second element consisted of three 30-minute stress support sessions delivered by POPPI nurses to acutely stressed patients (scores>7 on the IPAT), ideally starting the sessions in the critical care unit within 48 hours of IPAT screening and completing the three within a week (in the unit or on other wards). The three stress support sessions were based on psychological techniques evaluated in other populations and adapted for critical care patients. Techniques from psychological interventions for psychosis included establishing a collaborative relationship focused on reducing distress; managing patient concerns based on hallucinations and delusions; psychological education to reduce distressing interpretations of unusual experiences, reduce stigma, and encourage open communication; and provision of active coping strategies. Interventions were adapted to take account of critical care patients' likely physical, emotional or cognitive fragility.

Stress support sessions also included psychological techniques from trauma-related interventions to help people to identify unhelpful thoughts, interpretations and coping styles, and to think about their experiences in a less upsetting way. However some psychological interventions for trauma are not suitable for critical care patients. For example during stress support sessions, patients were encouraged to express their thoughts and feelings about their critical care experiences if they wish, but not to undertake 'reliving' their critical care stay, particularly as their trauma may be continuing.

The main objectives of the stress support sessions were for nurses to develop a trusting relationship with patients, so patients could discuss concerns which they might feel embarrassed or worried about communicating, and to reduce emotional distress. Stress support session one involved discussing and normalising common psychological reactions in critical care, helping patients to open up about worries, and exploring coping strategies. In the second stress support session, patients were encouraged to open up more fully about their fears; to identify highly stressful thoughts, and to learn how to find out if their worst fears were realistic. The third session began with summarising key messages and reviewing remaining problems. Nurses then helped patients to make a personal plan to cope with challenges ahead, to build realistic optimism about recovery and plan where to seek help if problems arise in future. Patients were asked to rate their stress levels on a "stress thermometer" (a simple tool commonly used by psychotherapists to rate stress levels during therapy on a scale of 0-10) at the beginning and end of each stress support session, to help nurses monitor how patients were feeling as the sessions progressed.

The third element of the complex intervention was a relaxation and recovery programme designed to help people practise new coping strategies, to provide meaningful activity and distraction, to and to learn from other patients' experiences, between stress support sessions. The POPPI app with calming music, relaxation, meditation, nature sounds/videos and patient recovery stories was loaded onto tablet computers loaned to

For peer review only - http://bmjopen.bmj.com/site/about/guidelines.xhtml

Table one: The three elements of the POPPI intervention to reduce stress in critical care patients

Patient interventions	Who receives?	Where?	Proposed mechanisms of change	Training methods	Who is trained?
Element one: Creating a therapeutic environment in critical care	All critical care patients	In the critical care unit	Raised staff awareness of patients' stress and psychological morbidity.<br>Staff motivated to reduce stressors in the unit.<br>Communication skills training for staff.<br>Emphasis on positive recovery messages.	An online training course<br><br>Promotional materials and local education sessions to reinforce key messages	All clinical critical care staff
Element two: Three stress support sessions for patients screened as acutely stressed	Patients screened by Intensive care psychological assessment tool (IPAT)[22] as acutely stressed (scores >7, range 0-20)	In the critical care unit, on wards following discharge from critical care	Help patients to express fears or concerns, and process traumatic memories (S1/2).<br>Reduce catastrophic misinterpretations by a) normalising stressful thoughts and feelings, and b) testing the reality of fears or concerns (S1/2)<br>Promote hope and motivation for recovery (S3)	A three-day face-to-face training course for 'POPPI' nurses to learn to deliver stress support sessions and coach patients in using the relaxation and recovery programme	Three "POPPI" nurses per critical care unit, selected by units with reference to suitability criteria
Element three: Relaxation and recovery programme on app, DVD and booklet (music, relaxation, meditation, patient recovery videos and self-help information)	Patients screened as acutely stressed and receiving stress support sessions	In the critical care unit, on wards (via tablet computer) and at home (via DVD and self-help booklet)	Provide distraction and meaningful activity.<br>Reduce stress by teaching patients relaxation and coping strategies (in S1/2)<br>Normalise emotional reactions, and promote optimism by providing former patients' recovery videos (In S1/2)<br>Promote recovery by providing information and helping to create a personal plan for future well-being (S3, DVD, booklet)	Provision of educational materials (pre-course theory booklet, manual and training folder) for POPPI nurses, and ongoing debriefing and support (clinical supervision) from trainers	

For peer review only

For peer review only - http://bmjopen.bmj.com/site/about/guidelines.xhtml

patients receiving stress support sessions. DVDs containing similar materials to the app, and a self-help booklet "Getting well, staying well" were given to patients to keep and take home.

An education package was developed to train staff in each element of the intervention. We adopted a 'blended learning' approach, combining online training and face-to-face teaching. Interactive and engaging online training courses are recommended by educationalists for the provision of knowledge to a large number of staff, while face-to-face training is suitable for adults acquiring new skills by practising and receiving feedback.[41] We evaluated training by measuring nurses' reaction to the courses, learning, and self-confidence in psychological skills.[42] The online training course to deliver element one, had four aims: to increase knowledge and understanding of the psychological impact of critical care; to learn to reduce stressors in the environment; to improve staff communication skills; and promote realistic hope for patients' recovery. It was co-designed with education experts as an interactive course using videos, colourful graphics, and quizzes. On completing the course, staff took an online knowledge test and received certificates if they passed.

Additionally a central face-to-face course was devised to train POPPI nurses to deliver all elements of the complex intervention. During the three-day course, psychological principles were taught and all three elements of the intervention were covered. A significant amount of time was devoted to skills practice in delivering stress support sessions, with the training team (a psychologist, nurses and patient representatives) observing and offering feedback. An additional day of feedback and assessment was held after POPPI nurses returned to their units and delivered sessions to at least one patient each. The day included nurse focus groups on their experience of delivering stress support sessions, and a competence assessment, in which nurses delivered stress support session two to a simulated patient (actor), observed by trainers who completed a checklist. Thereafter nurses had clinical supervision with trainers, during regular "debriefing and support" phone calls.

Associated training materials were developed, including an intervention manual for POPPI nurse, a set of slides for the three-day training course; and a training folder consisting of course hand-outs, patient scenarios, stress thermometers, summaries and checklists of each stress support session, and reflective note templates.

Key psychological outcomes were identified as PTSD and depression, as the most serious common psychological outcomes of critical care. Research in identifying modifiable critical care risk factors is more extensive for PTSD than for depression and anxiety, therefore PTSD was chosen as the primary outcome of the full trial.

For peer review only - http://bmjopen.bmj.com/site/about/guidelines.xhtml

FEASIBILITY AND PILOTING (STAGE TWO)

Methods

Once development of the complex intervention was completed, two separate feasibility studies were conducted , following a favourable ethical opinion from the National Research Ethics Service Committee South Central - Oxford B on 23 April 2014 (14/SC/0149). An Intervention feasibility study was held in two English adult general critical care units (UCLH and Watford General Hospital) and a different trial procedures feasibility study was held in two other hospitals (Bristol Royal Infirmary and Medway Maritime Hospital).

Intervention feasibility study

The aims of this study were to test the feasibility and acceptability of content and delivery of the complex intervention, and to get feedback from patients and staff, to help refine the intervention prior to full evaluation. No follow-up questionnaires were sent to patients in this study. Each hospital was asked to send three "POPPI" nurses to attend the central three-day face-to-face course to learn about content and delivery of the intervention. Once they returned to their hospitals, all clinical staff in their units were asked to complete the online training course on creating a therapeutic environment, and patient recruitment began. POPPI nurses also attended an additional day of feedback and assessment once each had conducted stress support sessions with at least one patient.

Inclusion criteria for patients were: Age 18 or greater; receipt of Level 2 or 3 care for 48 hours or more; Richmond Agitation Sedation Scale (RASS)[43] score between +1 and -1; English-speaking and ability to communicate orally; no pre-existing cognitive impairment, psychotic illnesses or chronic PTSD; Glasgow Coma Scale (GCS) score of 15; not terminally ill or receiving end-of-life care. Patients were provided with written and verbal information as part of the informed consent process. Consenting patients were screened for acute stress (>7 on the IPAT). Stress support sessions and a relaxation and recovery programme were delivered to acutely stressed patients by POPPI nurses.

Feasibility and acceptability of content and delivery of the intervention were assessed using quantitative and qualitative data from nurses and patients (see table two). Quantitative data included delivery rates of each element of the intervention, as well as scores/ratings from patient satisfaction questionnaires and staff training evaluation questionnaires (on satisfaction, learning and self-efficacy). Staff satisfaction and learning questionnaires were completed at the end of the online training course and the three-day face-to-face course. POPPI nurses also completed self-efficacy (confidence in psychological skills) questionnaires pre- and post- the three-day course, and on the additional feedback and assessment day, when a competence assessment was also carried out with a trainer for each nurse. Qualitative nurse data were collated from focus groups on the additional training day and at the end of the study, nurse debriefing sessions with trainers and from case report forms (CRFs). Once the intervention was embedded in the intervention sites, patients who received the stress support sessions and relaxation and recovery programme completed satisfaction questionnaires. Qualitative

For peer review only - http://bmjopen.bmj.com/site/about/guidelines.xhtml

patient data came from free-text sections on the satisfaction questionnaires and nurse-reported patient comments in the CRF. The above data were used to refine the intervention, where relevant.

Trial procedures feasibility study

The aim of this study was to determine recruitment and retention rates for a full trial. Patient eligibility criteria were the same as for the intervention feasibility study, and consent was obtained. The intervention was not delivered in this study. At five months post-recruitment, patients were sent follow-up psychological and health questionnaires proposed for the full trial. These included the PTSD Symptom Severity Scale – Self Report (PSS-SR)[44] and the Centre for Epidemiologic Studies Depression Scale (short form) (CESD-10).[45]

Results

Intervention feasibility study

Feasibility of delivering the intervention

Ten POPPI nurses (100%) from the two hospitals completed a central three-day face-to-face training course (due to personnel issues, this included training extra nurses at one site). When they returned to their hospitals, online training became available for all staff, and patient recruitment was opened for 5.5 months. 283 (84%) staff in the two critical care units took the online training course, surpassing the target rate of 80%, with 277 (98%) passing the end of course assessment. 127 patients were recruited to the study, all were screened with the IPAT, and 51 (40%) were identified as acutely stressed (see figure two). 44 stressed patients (86%) received stress support sessions, with 39 (77%) receiving two or three sessions and five having one session only. Seven patients (14%) had no sessions because they declined, were discharged home early or deteriorated clinically. Median duration of sessions one to three was 35, 30 and 30 minutes respectively. 40 patients (90%) received a tablet computer, and 27 (61%) were given a DVD and patient booklet to keep.

Acceptability of content of the intervention

All nurse and patient feasibility questionnaires had scales of 0-5 per item, with 4 or 5 defined by us as "good" scores or ratings. Staff gave the online training course good ratings for: Stimulating (73%, 189/260); useful (86%, 223/260) and well-designed (84%, 216/257). All ten POPPI nurses rated the face-to-face course as good (stimulating, useful, well-conducted, motivating), with nine (90%) rating relevance to their new role as good. There was a large increase in nurse self-efficacy, from 30% good scores (21/70 across seven items) pre-course, to 73% good scores (51/70) post-course. The proportion of 73% good scores was maintained at follow-up (on the feedback/assessment day). 87% (69/79) of post-course learning scores on eight items were good.

Once the intervention was embedded, fifteen patients completed a satisfaction questionnaire. Satisfaction with stress support sessions was rated as good by 14 (93%) patients. Fifteen patients (100%) gave good ratings for nurses understanding them and normalising their fears. Slightly fewer patients thought their stressful thoughts (13, 87%) or feelings (12, 80%) were reduced by the sessions. Twelve (80%) rated the number and duration of

For peer review only - http://bmjopen.bmj.com/site/about/guidelines.xhtml

sessions as “just right”. Satisfaction with the relaxation and recovery programme was somewhat less than for the stress support sessions. Ten of 14 (71%) rated contents of the tablet app as good, while 10 of 15 (67%) thought that they learned good coping strategies from the programme. Stress, rated on the stress thermometer (0-10), was reduced by median 3 points (IQ range 1-5 points) from the start of session one to the end of session three among the 25 patients who received all three sessions.

Quantitative feasibility and acceptability data from the intervention study are presented fully in table two. Qualitative data on the three elements are also summarised in the table, but specifics, both positive and negative, are described in more detail as follows. Nurses reported that the online training course had raised awareness, changed staff thinking and led to a better environment in their units. However, they said design improvements, more practical advice and summaries of key messages were needed. Delivery of element one was challenging, as POPPI nurses were too busy to support other staff in creating a therapeutic environment. It was suggested this responsibility should be shared by the wider critical care team, and further educational/ promotional materials were needed.

Nurse feedback on content of the second element was that stress support sessions were welcomed by patients and rewarding for nurses. Challenges included ensuring patients understood the sessions and knowing how to handle very serious issues that might be raised. Delivery of stress support sessions could be difficult to fit into heavy clinical workloads, especially if patients postponed sessions due to fatigue or sickness.

Comments on the three-day course included positive ones such as “best course ever”; “feel privileged” to be part of it. However some nurses said they found it hard work and tiring. They suggested it would be less intense if psychological theory was delivered to be studied in advance, rather than presented during the course, and that skills practice should involve actors (instead of fellow trainees) to play patients. They found the POPPI nurse training manual invaluable, but suggested some re-structuring and clarification in the presentation of stress support sessions, with better linkage to appendices. All nurses found the competence assessment on the additional training day stressful. Most said they welcomed the regular debriefing and support calls with trainers, finding them useful for reflection and building greater confidence.

Patient feedback on the stress support sessions was mainly positive, and included “every hospital should have it”, “it seemed a life saver”, “proves that I am normal but under the influence of morphine” and “I hope that POPPI will eventually be used in all ICUs. Thank you for the support.” Criticisms included one patient saying that the critical care environment had not been improved in response to his concerns, and some patients wanting more than three stress support sessions. Patients also responded well to the relaxation and recovery programme. Some patients preferred patient stories, some relaxation exercises, some meditation and some calming music. However some found the tablet computers confusing and difficult to use, due to unfamiliarity with the technology, or reduced dexterity. Unfortunately the tablets used in the intervention feasibility study did not have good touch-sensitivity. Some patients were disappointed that the DVDs did not include the calming music (due to copyright issues).

For peer review only - http://bmjopen.bmj.com/site/about/guidelines.xhtml

Table two: Feasibility, acceptability and refining the three elements of the psychological intervention

Elements of intervention	Content/ delivery of element	Feasibility and acceptability indicators – quantitative and qualitative	Feasibility and acceptability results	Refinement of the intervention post-feasibility study
Element one: Creating a therapeutic environment in critical care	Content of online training course	Training course ratings by all staff (% with 4 or 5(0-5) or "good")<br><br>Favourite parts of course – all staff<br><br>Nurse qualitative feedback *	Stimulating 73%; Useful 86%; Well-designed 84%; Right length 80% (n=260, but missing data for some items)<br><br>Factual information 42%; Patient stories 35%; Communication videos 13%; Tests 10% (n=260)<br><br>Staff positive, suggested minor improvements	Online training course shortened and made more visually appealing, with more practical advice on reducing stressors in critical care units and clearer presentation of key messages
	Delivery of online training and creating a therapeutic environment	Staff taking course (target: 80%)<br><br>Staff passing final test (score >80%)<br><br>Staff learning scores (% with 4 or 5(0-5) or "good")<br><br>Nurse qualitative feedback	283 (84%)<br><br>277 (98%)<br><br>74% (n=259)<br><br>POPPI nurses lacked time, due to workload, to support staff in creating therapeutic environment	Provision of training, display materials and slide-sets for seminars/ workshops, for local education teams to support and motivate staff in creating a therapeutic environment
Element two: Three stress support sessions for patients screened as acutely stressed	Content of screening	Previously validated[34]		
	Delivery of screening	Consenting patients screened<br>Screened as acutely stressed	127 (100%)<br><br>51 (40%)	
	Content of stress support sessions	Median (IQR) difference in patient stress thermometer scores (0 - 10)<br><br>Patient satisfaction with stress support sessions (% with 4 or 5(0-5) or "good")<br><br>Patient qualitative feedback<br><br>Nurse qualitative feedback	Median difference from start session 1 to end session 3 was -3.0 (-5.0, -1.0) (n=25 patients who had all 3 sessions)<br><br>Overall 93%; Helped express fears 93%; Nurse understanding 100%; Nurse normalised fears 100%; Fewer stressful thoughts 87%; fewer stressful feelings 80%; number/duration of sessions 80% (n=15, missing data some items)<br><br>Stress support from nurses was very helpful<br><br>Rewarding, but challenging to explain to patients	Content of stress support sessions clarified for POPPI nurses and patients by reorganising sessions from five components each, into three common components in all sessions and three individual components per session. Manual became more tightly focused on stress support sessions (rather than the whole intervention) with clearer signposting to and between sections
	Delivery of stress support sessions	Number of stress support sessions patients had<br><br>Median duration of sessions (minutes = m)<br><br>Nurse qualitative feedback	25 (49%) had three sessions; 14 (28%) had two sessions; 5 (10%) one session; 7 (14%) had none<br><br>Session one: 35m, Session 2: 30m, Session 3: 30m<br><br>Hard to fit sessions in with ordinary duties, especially if patients postponed. Patients missed session three if they were discharged home early	Ensure buy-in and support for POPPI nurses from clinical, education and research staff from the start, making it a team effort.<br>If hospital discharge is near, sessions two and three can and should be delivered together

For peer review only

For peer review only - http://bmjopen.bmj.com/site/about/guidelines.xhtml

	Content of POPPI nurse face-to-face training course - three days and a feedback/ assessment day	Nurse feedback – post-course questionnaire (% with 4 or 5(0-5) or “good”)<br><br>Nurse self-efficacy (in delivering psychological support) – pre-/post-course and feedback day questionnaires. (% of 4 or 5(0-5) or “good” ratings of items<br><br>Nurse course learning – an 8-item post-course knowledge questionnaire - (% of 4 or 5(0-5) or “good” scores for items)<br><br>Trainer assessment of nurse competence using six-item checklist (scores 0-12; pass =6)<br><br>Nurse qualitative feedback	Stimulating 100%; Useful 100%; Relevant 90%<br>Well conducted 100%; Motivating 100% (n=10)<br><br>Big increase in self-efficacy from pre- to post-course; maintained at follow-up. 30% of scores (21/70) across items were good pre-course; 73% were good scores (51/70) post-course and on feedback day<br><br>87% of scores (69/79) were “good” for learning on:acute stress; screening; aims of stress support sessions, normalising, psycho-education, communication style, stressful thinking, checking out fears, coping<br><br>100% passed (9 on first attempt, 1 on second)<br>Median (IQR) score of passes 9 (9, 10)<br><br>Course highly valued but tiring. Skills practice stressful. Competence assessment on follow-up day stressful	To reduce burden and increase self-efficacy, the three-day course became more focused on stress support sessions, particularly session two, seen as the most difficult session to deliver. More emphasis on skills practice, with actors (not fellow trainees) playing patients. Wider spectrum of patient scenarios used.<br><br>Assessment to be re-framed as “skills development” and carried out one-to-one, with a trainer playing the patient
	Delivery of three-day training course	Number of required trainees attending	10 (100%)**	Some modules dropped from course; pre-course booklet on psychological principles provided
	Content: Debriefing support by trainers	Nurse qualitative feedback	Debriefing calls useful for reflection, confidence<br>Assessment should be part of ongoing support	Assessment to be one-to-one confirmation of skills (as above), as part of ongoing debriefing and support
	Delivery: Debriefing and support	Nurse qualitative feedback	Nurse debriefing and support should start earlier	First debriefing call after first POPPI patient
Element three: Relaxation and recovery programme on App, DVD and booklet (music, relaxation, meditation, patient recovery videos, self-help information)	Content of relaxation and recovery programme	Patient satisfaction with content of programme - (% with 4 or 5(0-5) or “good”)<br><br>Nurse-reported qualitative patient feedback	Content on tablet computer app 71%; Useful post-ICU coping ideas 67% (15 patients, some missing data)<br><br>Varied preferences; relaxation, meditation, nature sounds, patient stories or calming music. Some disappointed not to find calming classical music on DVD.	Content and design of the relaxation app were improved. Balance of contents of DVD improved, and calming classical music tracks added. Layout and readability of the patient booklet improved.
	Delivery of relaxation and recovery programme	Patients receiving tablet in session one<br><br>Patients receiving DVD or booklet<br><br>Nurse-reported qualitative patient feedback	40 (90%)<br><br>27 (61%)<br><br>Some liked tablet, others found it hard to use , some preferred DVD or patient booklet	Usability of the relaxation app was improved, to make it easier for the less dextrous. Higher- spec tablets, including better touch sensitivity, identified for use in future trial<br><br>DVD and booklet to be provided in session two, so more patients would receive them (many missed receiving session three due to being sick or discharged from hospital)

** All nurse and patient qualitative data described on p.11. **Four additional nurses were trained at one site due to personnel issues*

For peer review only - http://bmjopen.bmj.com/site/about/guidelines.xhtml

Refinement of the complex intervention

We refined the complex intervention based on key points emerging from the intervention feasibility study data (see table two, last column). To refine element one, we made the online training course shorter, more attractive and clearer, and included more practical advice and emphasis on key messages. We created new slide-sets and display materials for units to reinforce the key messages to their staff. We decided that the wider critical care team should be involved in creating the therapeutic environment, so that POPPI nurses could focus on delivering stress support sessions to patients.

To improve element two, the three-day face-to-face POPPI nurse course was more tightly focused on the stress support sessions, with more emphasis on skills practice, and actors playing patients, to reduce the burden on trainees. Some modules, such as a booklet on psychological principles, were provided to POPPI nurses in advance, to reduce the intensity of the course. The nurse competence assessment was reframed as a skills development assessment and carried out less formally as part of ongoing one-to-one debriefing and support sessions with a trainer. The manual was made simpler and clearer, to improve nurse and patient understanding of the components of the stress support sessions. As many patients in the feasibility study did not receive the third support session due to early hospital discharge, it was decided that sessions two and three could and should be delivered together in those circumstances, if patients agreed and were well enough.

To refine element three, the content, design and usability of the relaxation app was improved, and tablets with better touch sensitivity were identified for use in the future trial. Small improvements were made to the design and content of the DVD and patient booklet, and it was decided they would be given to patients in session two rather than session three, so that more patients would receive that element of the intervention, even if they were due to leave hospital after session two.

Trial procedures feasibility study

There were two-month recruitment and five-month follow-up periods for this study. Of 160 eligible patients in the two sites, 86 (53.8%) provided consent to take part (see figure three). Of these, nine (10.5%) had died by 5 months, but 62 (80.5%) completed the follow-up questionnaire. Overall completeness of the primary outcome measure (the PSS-SR[44]) was very good. For a total of 1054 fields, only 24 (2.3%) had missing data.

DISCUSSION

The objectives of the POPPI intervention development study were met: we developed and standardised a nurse-led psychological intervention to reduce patients' acute stress in critical care; we created a staff package to deliver it; we tested feasibility of the intervention and trial procedures, and refined the intervention. The intervention had three inter-connected elements: Creating a therapeutic environment; stress support sessions delivered by selected, trained nurses to acutely stressed patients, and a relaxation and recovery programme for acutely stressed patients. To support delivery of the intervention, we devised and created a relaxation and recovery App, DVD and booklet for patients; an online training course for all critical care staff; and a training

For peer review only - http://bmjopen.bmj.com/site/about/guidelines.xhtml

package for POPPI nurses, including a manual of the three stress support sessions, a three-day face-to-face course with training folder, and a debriefing and support programme.

An intervention feasibility study showed that delivery and content of the complex intervention were feasible and acceptable for patients and staff, and led to a refined version of the intervention, ready for evaluation in a cluster-randomised clinical trial (cRCT). A separate trial procedures feasibility study demonstrated that the necessary recruitment and retention rates for a cRCT could be achieved.

Strengths of this study were that it resulted in the development of a novel psychological intervention to improve psychological outcomes. Importantly, the intervention has three-interconnected elements: to reduce stress in the environment and improve staff-patient communication; to provide targeted support for the most stressed patients; and to provide stress relief and meaningful activity for patients. The intervention builds on prior work investigating the psychological impact of critical care and was developed using a rigorous framework[29] with input from patient representatives and supervision from an expert team. More than 200 patients and 280 staff were involved in the feasibility studies. The psychological intervention was confirmed to be feasible and acceptable for patients and staff. Data collection is underway to evaluate the intervention in a cRCT.

Limitations were that the intervention was piloted in only two sites, and limited patient feedback was gathered. No efficacy data were collected during the feasibility studies.

The POPPI intervention addresses evidence that critical care patients suffer acute and long-term stress.[7-10] Despite increasing recognition[13] that interventions to reduce critical care stress are needed, little is known about what would be most effective. Studies of early interventions such as music, relaxation techniques and psychological support in critical care showed promise.[23,24] POPPI is the first complex psychological intervention designed to be delivered early, during the critical care admission, to prevent PTSD and other long-term morbidity.

Conclusion

The POPPI psychological intervention to reduce stress and prevent long-term morbidity for critical care patients was feasible, acceptable and ready for evaluation in a cluster-randomised clinical trial.

Supplementary material: Click here to view

Contributors

Early conceptual and development work for the POPPI intervention was carried out by DW, JWei, DHo, MM, DS, JW, NA, MH, CW and CB at University College London Hospitals. Further development of the intervention and design of the feasibility phase of the POPPI study were carried out with KR, SH, DHa, PM, ARB, DD'A and VB. DW drafted the initial manuscript with input from KR. All authors revised the manuscript critically and gave their approval of the final version. DW is the guarantor.

For peer review only - http://bmjopen.bmj.com/site/about/guidelines.xhtml

Acknowledgements

Daniel Freeman (chair of the education and psychology advisory group), Dane Goodsman (clinical educationalist), Rob Beer (online training designer), Daniel Zanitchkhah (app designer), Derek Tutssel (video director), Lih-Mei Liao, Wendy Harris, Steve Saunders, Ruth Canter, Rahi Jahan, Blair McLennan, Emma Wood. David Aaronovitch, Sheila Richards, Peter Cross, Rajadurai Sunderalingham, Kate Baden-Fuller, Caroline Knight, and other members of the UCLH patients and family advisory group.

"POPPI" nurses and site PIs:

University College Hospital, London: Emma Davies, Emma Thompsett, Rosalind Edwards, David Brealey, Magda Rocha, Jung Ryu, Georgia Bercades. Watford General Hospital: XioBei Zhao; Jennie Haydock, Kalpana Giri Ghimire, Lillian Norris, James Cunningham, Sarah- Jane Turner, Tracey Temple

Medway Maritime Hospital: Catherine Plowright, Claire Pegg, James Cullinane

Bristol Royal Infirmary: Katie Sweet, Sanjoy Shah, Lisa Grimmer.

Funding

This project was funded by the National Institute for Health Research (NIHR) Health Services and Delivery Research (HS&DR) Programme (Project: 12/64/124). DW also received funding from the University College London/University College London Hospitals NIHR Biomedical Research Centre. The views and opinions expressed in this paper are those of the authors and do not necessarily reflect those of the HS&DR, NIHR, NHS or the Department of Health. The funding body had no role in the design of the study and collection, analysis, and interpretation of data and in writing the manuscript.

Ethics

The two feasibility studies were granted favourable ethical opinion by the NRES Committee South Central - Oxford B research ethics committee on 23 April 2014 (reference: 14/SC/0149), were managed by the ICNARC CTU and prospectively registered on the ISRCTN Registry (ISRCTN61088114).

Transparency Declaration

The lead author affirms that the manuscript is an honest, accurate transparent account of the study being reported, that no important aspects of the study have been omitted, and that any discrepancies from the study as planned, have been explained.

Copyright

The Corresponding Author has the right to grant on behalf of all authors and does grant on behalf of all authors, a worldwide licence to the Publishers and its licensees in perpetuity, in all forms, formats and media (whether known now or created in the future), to i) publish, reproduce, distribute, display and store the Contribution, ii) translate the Contribution into other languages, create adaptations, reprints, include within collections and create summaries, extracts and/or, abstracts of the Contribution, iii) create any other derivative

For peer review only - http://bmjopen.bmj.com/site/about/guidelines.xhtml

work(s) based on the Contribution, iv) to exploit all subsidiary rights in the Contribution, v) the inclusion of electronic links from the Contribution to third party material where-ever it may be located; and, vi) licence any third party to do any or all of the above. We attest that we have obtained appropriate permissions and paid any required fees for use of copyright protected materials.

Competing interests

All authors have completed the ICMJE uniform disclosure form at www.icmje.org/coi_disclosure.pdf and declare: no support from any organisation for the submitted work; no financial relationships with any organisations that might have an interest in the submitted work in the previous three years; no other relationships or activities that could appear to have influenced the submitted work."

No additional data available

References

1. Wade DM. Doctoral thesis: Prevalence and predictors of psychological morbidity and quality of life after discharge from Intensive Care. University College London, 2010.
2. Puntillo KA, Arai S, Cohen NH, et al. Symptoms experienced by intensive care unit patients at high risk of dying. Critical care medicine 2010;**38**(11):2155-60.
3. Novaes MA, Knobel E, Bork AM, et al. Stressors in ICU: perception of the patient, relatives and health care team. Intensive care medicine 1999;**25**(12):1421-6.
4. Alonso-Ovies A, Heras La Calle G. ICU: a branch of hell? Intensive care medicine 2016;**42**(4):591-2.
5. Nelson JE. The symptom burden of chronic critical illness. Critical care medicine 2004;**32**.
6. Jones C, Griffiths RD, Humphris G, et al. Memory, delusions, and the development of acute posttraumatic stress disorder-related symptoms after intensive care. Critical care medicine 2001;**29**(3):573-80.
7. Wade DM, Howell DC, Weinman JA, et al. Investigating risk factors for psychological morbidity three months after intensive care: a prospective cohort study. Critical care (London, England) 2012;**16**(5):R192.
8. Parker AM, Sricharoenchai T, Raparla S, et al. Posttraumatic stress disorder in critical illness survivors: a metaanalysis. Critical care medicine 2015;**43**(5):1121-9.
9. Nikayin S, Rabiee A, Hashem MD, et al. Anxiety symptoms in survivors of critical illness: a systematic review and meta-analysis. General hospital psychiatry 2016;**43**:23-29.
10. Rabiee A, Nikayin S, Hashem MD, et al. Depressive Symptoms After Critical Illness: A Systematic Review and Meta-Analysis. Critical care medicine 2016;**44**(9):1744-53.
11. Huang M, Parker AM, Bienvenu OJ, et al. Psychiatric Symptoms in Acute Respiratory Distress Syndrome Survivors: A 1-Year National Multicenter Study. Critical care medicine 2016.
12. Bienvenu OJ, Colantuoni E, Mendez-Tellez PA, et al. Cooccurrence of and remission from general anxiety, depression, and posttraumatic stress disorder symptoms after acute lung injury: a 2-year longitudinal study. Critical care medicine 2015;**43**(3):642-53.
13. National Institute of Health and Care Excellence (NICE) quality standard 158 - Rehabilitation after critical illness in adults: NICE, 2017
14. Cuthbertson BH, Rattray J, Campbell MK, et al. The PRaCTICaL study of nurse led, intensive care follow-up programmes for improving long term outcomes from critical illness: a pragmatic randomised controlled trial. BMJ (Clinical research ed) 2009;**339**:b3723.
15. Jensen JF, Thomsen T, Overgaard D, et al. Impact of follow-up consultations for ICU survivors on post-ICU syndrome: a systematic review and meta-analysis. Intensive care medicine 2015;**41**(5):763-75.
16. Mehlhorn J, Freytag A, Schmidt K, et al. Rehabilitation interventions for postintensive care syndrome: a systematic review. Critical care medicine 2014;**42**(5):1263-71.
17. Jensen JF, Egerod I, Bestle MH et al. (2016) A recovery program to improve quality of life, sense of coherence and psychological health in ICU survivors: a multicenter randomized controlled trial, the RAPIT study. Intensive Care Med, 42(11): 1733-43.
18. Jones C, Backman C, Capuzzo M, Egerod I, Flatten H, Granja C et al. Intensive care diaries reduce new-onset post traumatic stress disorder following critical illness: a randomised, controlled trial. Crit Care 2010; 14:R168

For peer review only - http://bmjopen.bmj.com/site/about/guidelines.xhtml

19. Cox CE, Hough CL, Carson SS, White DB, Kahn JM, Olsen MK, et al. Effects of a telephone- and web-based coping skills training program compared with an education program for survivors of critical illness and their family members: a randomized clinical trial. Am J Respir Crit Care Med 2018;197:66–78.
20. Davydow DS, Zatzick D, Hough CL, et al. In-hospital acute stress symptoms are associated with impairment in cognition 1 year after intensive care unit admission. Annals of the American Thoracic Society 2013;**10**(5):450-7.
21. Jones C, Backman C, Capuzzo M, et al. Precipitants of post-traumatic stress disorder following intensive care: a hypothesis generating study of diversity in care. Intensive care medicine 2007;**33**(6):978-85.
22. Peris A, Bonizzoli M, Iozzelli D, et al. Early intra-intensive care unit psychological intervention promotes recovery from post traumatic stress disorders, anxiety and depression symptoms in critically ill patients. Critical care (London, England) 2011;**15**(1):R41.
23. Papathanassoglou ED. Psychological support and outcomes for ICU patients. Nursing in critical care 2010;**15**(3):118-28.
24. Wade DF, Moon Z, Windgassen SS, et al. Non-pharmacological interventions to reduce ICU-related psychological distress: a systematic review. Minerva anestesiologica 2016;**82**(4):465-78.
25. Fowler D, Garety P, Kuipers E. *Cognitive behaviour therapy for psychosis: theory and practice.* Chichester: John Wiley and Sons, 1995.
26. van der Gaag M, Valmaggia LR, Smit F. The effects of individually tailored formulation-based cognitive behavioural therapy in auditory hallucinations and delusions: A meta-analysis. Schizophrenia Research 2014; **156** (1): 30-37
27. Hobfoll S, Watson P, Bell C, et al. Five Essential Elements of Immediate and Mid-Term Mass Trauma Intervention: Empirical Evidence. FOCUS 2009;**7**(2):221-42.
28. Brewin CR. Re-experiencing traumatic events in PTSD: new avenues in research on intrusive memories and flashbacks. Eur J Psychotraumatol 2015;**6**:27180.
29. Craig P, Dieppe P, Macintyre S, et al. Developing and evaluating complex interventions: the new Medical Research Council guidance. BMJ (Clinical research ed) 2008;**337**:a1655.
30. Wade D, Hardy R, Howell D, et al. Identifying clinical and acute psychological risk factors for PTSD after critical care: a systematic review. Minerva anestesiologica 2013;**79**(8):944-63.
31. Wade DM. MSc thesis: The psychology of weaning from mechanical ventilation: a mixed methods study of mood, control, support and communication in the intensive care unit. University of London, 2006.
32. Wade DM. Portfolio of competence, stage II health psychology: The British Psychological Society, 2011.
33. Wade DM, Brewin CR, Howell DC, et al. Intrusive memories of hallucinations and delusions in traumatized intensive care patients: An interview study. British journal of health psychology 2014.
34. Wade DM, Hankins M, Smyth DA, et al. Detecting acute distress and risk of future psychological morbidity in critically ill patients: validation of the intensive care psychological assessment tool. Critical care (London, England) 2014;**18**(5):519.
35. Wade D, Moon Z, Windgassen S, et al. Nonpharmacological interventions to reduce short-term or long-term psychological stress in ICU patients: a systematic review. Critical Care 2015;**19**(Suppl 1):P556-P56.
36. Lazarus RS, Folkman S. *Stress, appraisal, and coping.* New York, NY: Springer Publishing Company, 1984.
37. Silverman J, Kurtz S, Draper J. *Skills for Communicating with Patients*. Oxford: Radcliffe Publishing, 2013.
38. Bowers L, Brennan G, Winship G, et al. How expert nurses communicate with acutely psychotic patients. Mental Health Practice 2010;**13**(7):24-26.
39. Michie S, van Stralen MM, West R. The behaviour change wheel: a new method for characterising and designing behaviour change interventions. Implementation science 2011;**6**:42.
40. Stoltenberg CD, McNeill B, Delworth U. *IDM supervision: An integrated developmental model for supervising counselors and therapists.* San Francisco: Jossey-Bass, 1998.
41. Bullock I, Davis M, Lockey A, et al. *Pocket Guide to Teaching for Clinical Instructors*. 3 ed. West Sussex: John Wiley & Sons Ltd., 2016.
42. Kirkpatrick DL, Kirkpatrick JD. *Implementing the Four Levels*. San Francisco, CA: Berrett-Koehler Publishers, 2007.
Sessler CN, Gosnell MS, Grap MJ, et al.

43. The Richmond Agitation-Sedation Scale: validity and reliability in adult intensive care unit patients. American journal of respiratory and critical care medicine 2002;**166**(10):1338-44.
44. Foa EB, Cashman L, Jaycox L, et al. The validation of a self-report measure of posttraumatic stress disorder: the Posttraumatic Diagnostic Scale. . Psychological Assessment 1997;**9**:445-51.
45. Irwin M, Artin KH, Oxman MN. Screening for depression in the older adult: criterion validity of the 10-item Center for Epidemiological Studies Depression Scale (CES-D). Archives of internal medicine 1999;**159**(15):1701-4.

Figure legends

Figure one: Use of Medical Research Council framework for developing and evaluating complex interventions

Figure two: Patient flow in the intervention feasibility study

Figure three: Consort diagram for the trial procedures feasibility study

For peer review only - http://bmjopen.bmj.com/site/about/guidelines.xhtml

For peer review only

For peer review only - http://bmjopen.bmj.com/site/about/guidelines.xhtml

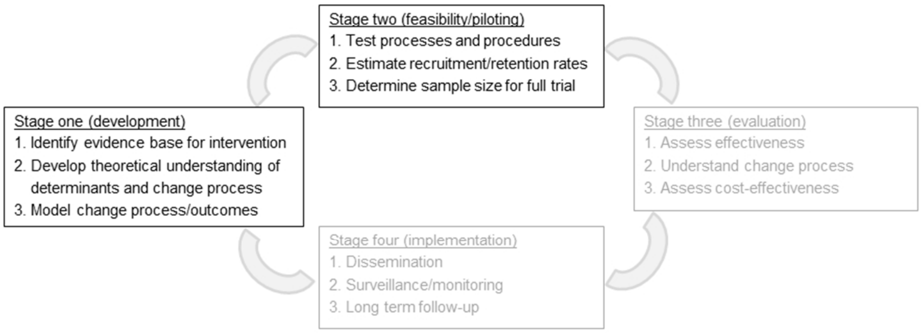

Use of Medical Research Council framework for developing and evaluating complex interventions

81x60mm (300 x 300 DPI)

For peer review only - http://bmjopen.bmj.com/site/about/guidelines.xhtml

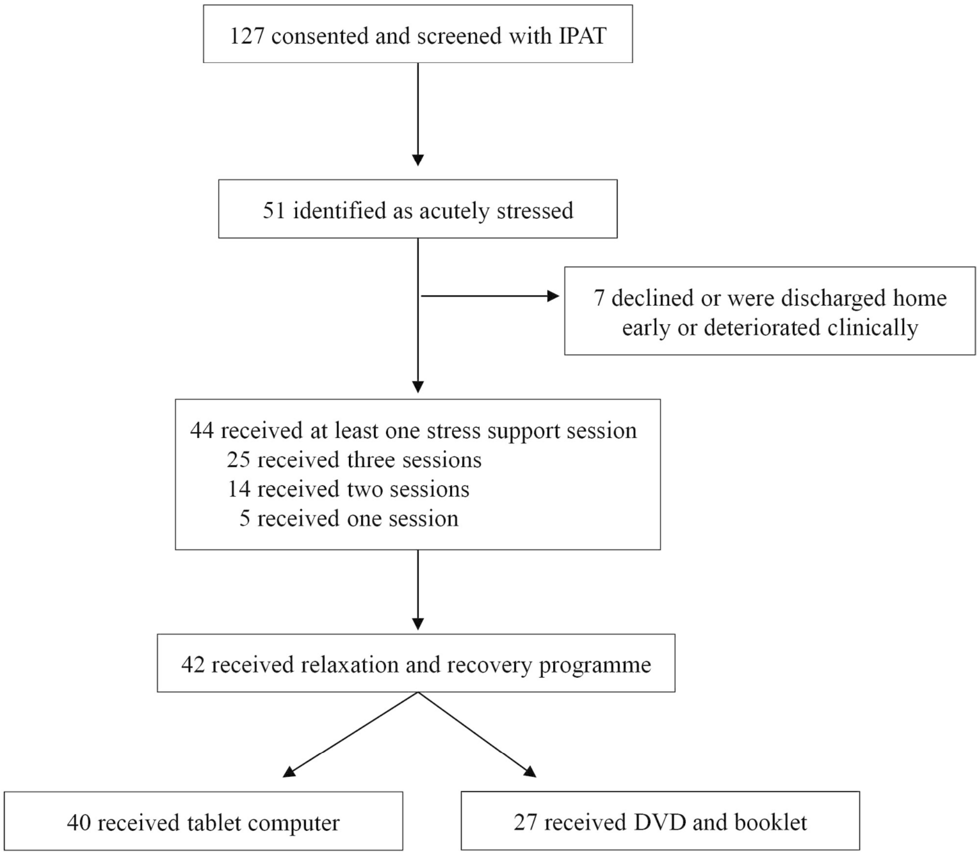

Patient flow in the intervention feasibility study

172x153mm (300 x 300 DPI)

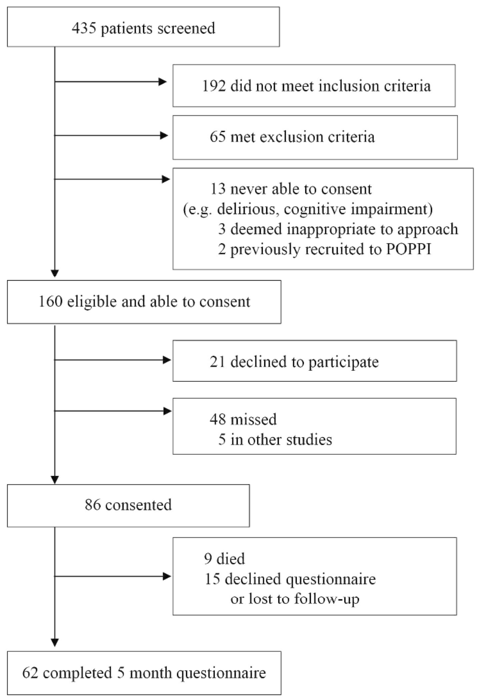

Consort diagram for the trial procedures feasibility study

254x190mm (300 x 300 DPI)

Electronic supplement one: Development phases of an intervention to reduce patient stress in critical care

MRC model – steps and stages	Preparatory phase 2006-11*	Early modelling 2011-13†	Feasibility and refinement phase 2013-2015‡
Stage one, step one<br>Identify the evidence base	Identified systematic reviews and studies of psychological outcomes, risk factors and conducted new systematic reviews of post-traumatic stress, depression, anxiety post-ICU[1]<br><br>Literature review of ICU stressors, reactions, memories, stress measurement and stress reduction interventions[1]	Updated evidence base - identified new systematic reviews and studies of ICU psychological outcomes, risk factors, stress, stress measurement, stress reduction interventions[7,20,22]<br><br>Began systematic review of psychosocial interventions (psychological, music, mind-body therapies) to reduce ICU-related stress[24,35]	Updated evidence base of studies and systematic reviews[8,12,33]<br><br>Completed systematic review of interventions to reduce critical care stress[24,35]
Stage one, step two<br>Develop a theoretical understanding of determinants and processes of change	Patient studies: acute stress in ICU and psychological outcomes:<br>1. Quantitative study (n=40)[31]<br>2. Quantitative study (n=157)[7]<br>3. Qualitative study (n=7) [31]<br>4. Qualitative study (n=17)[33]<br><br>Developed hypotheses about causes of critical stress[1,7,31,32]<br><br>Explored nurse perceptions of patients' psychological needs and nurses' ability to attend to them: Content analysis of 5 focus groups at GSTT NHS trust with 125 nurses[32]<br><br>Identified communication[37] and adult learning methods** to inform development of training to enhance critical care nurse communication and psychological skills	Patient and relatives advisory group set up at UCLH; regular meetings to brainstorm and generate research ideas about patients' psychological needs and how they could be met<br><br>Improved conceptualisation and measurement of acute ICU stress: Development and validation of the Intensive care psychological assessment tool[34]<br><br>Identified psychological approaches (to stress and coping[36], psychosis[25-26] and trauma[27-28]) that could be adapted for critical care patients<br><br>Identified appropriate relaxation and recovery methods	Identified behaviour change methods[39] relevant to changing staff behaviours to create a therapeutic environment<br><br>Identified clinical supervision methods for non-experts delivering psychological support[40]
Stage one, step three<br>Model process and outcomes	Developed, piloted and evaluated a training day at GSTT to train critical care nurses in communication/psychological skills (utilising actors)[32]	Consultation with experts and discussions between UCLH, ICNARC and patient representatives, to develop the basic structure of a psychological intervention consisting of three interconnected elements: Creating a therapeutic environment; three stress support sessions; and a relaxation and recovery programme. Each element to have associated training components (online, face-to-face and training materials)<br><br>Early development of stress support sessions based on psychological principles at	Formed Expert Psychological Advisory Group of medical educationalist, psychologists, patients and the research team, to inform the final phase of POPPI†† intervention development<br><br>Further development of stress support sessions by senior nurses and psychologists<br><br>Created materials to deliver a complex intervention - nurse manual, checklists, notes and summaries<br><br>Researched/created content for and design of a relaxation and recovery app, a DVD and a

For peer review only

For peer review only - http://bmjopen.bmj.com/site/about/guidelines.xhtml

		UCLH – iterative process with psychologist and patients, supervised by senior psychologists (CB, JW, LML and staff from PICUP)<br><br>Identification of suitable materials for the relaxation/recovery programme<br><br>Post-traumatic stress selected as primary outcome (based on evidence identified in step one – more evidence of risk factors than for other psychological outcomes)	psychological well-being booklet for patients<br><br>Created training courses and materials for staff to deliver the psychological intervention:<br>Online training course, all staff<br>3-day face to face course for POPPI nurses, feedback/assessment day , training folder<br>A programme of debriefing and support for POPPI nurses
Stage two Feasibility and Piloting		Designed two separate feasibility studies – intervention feasibility study and the trial procedures feasibility study	Conducted two feasibility studies – intervention feasibility study and trial procedure study<br><br>Refined the complex intervention based on quantitative and qualitative data from patients and staff in the intervention feasibility study

**Kings College London, Guys and St Thomas hospitals Trust, University College/University College Hospital, London*
†University College Hospital (UCH) and Intensive Care National Audit and Research Centre (ICNARC);
‡ ICNARC and UCH
*** 43. Kolb DA. Experiential learning: experience as the source of learning and development. 1 ed. Englewood Cliffs, NJ: Prentice Hall, 1984.*
†† POPPI – Provision Of Psychological support to People in Intensive care
CB Chris Brewin (author), JW John Weinman (author) LML Lih-Mei Liao, Consultant Clinical Psychologist, University College London Hospitals (UCLH) . PICUP -Psychological interventions clinic for outpatients with psychosis, Maudesley Psychology Centre, Maudesley Hospital, London

Electronic supplement two

This supplement presents further details of the final post-feasibility version of the three-element POPPI complex intervention. This version is being evaluated in the POPPI cRCT.

Element one: Creating a therapeutic environment

Online training course
A key part of element one is the POPPI online training course: Key skills in Psychological Care. Training is undertaken by all clinical critical care staff to aid the creation of a calm, less stressful environment by reducing stressors in the environment and using good communication with patients. The course takes about 30 minutes and comprises the following five sections:

1 Understanding the stresses of intensive care patients
2 Reducing stress and fear in patients
3 Communicating with distressed patients
4 Inspiring patients with confidence and hope
5 Summary and assessment

The course has a balance of concise, readable text with graphics and other visual or audio aids, and test-yourself questions at the end of each section (with informative feedback). Video clips include former patients talking about their experiences of critical care, and nurses modelling good communication strategies with patients. Staff who pass the assessment (a score of 80% or more) receive a certificate.

Element two: Three stress support sessions

Three, one-to-one, stress support sessions lasting approximately 30 minutes each, are delivered to a patient by the same trained POPPI nurse, starting when patients are awake and alert, either in the critical care unit or following discharge to the hospital ward. The three sessions should be delivered within a week (for the pragmatic reason that many patients may be discharged from hospital around that time).

All stress support sessions contain three common components – starting the session; building rapport and finishing the session - and three individual components per session. Starting a session includes introductions, outlining aims, measuring stress and explaining confidentiality, note-taking and risk-reporting. Building rapport involves focusing throughout all the sessions on creating a relationship with the patient based on openness, honesty, warmth and listening. Finishing a session involves summarising, getting patient feedback, agreeing practice, re-measuring stress, agreeing wording for notes and arranging the next session.

The three individual components nurses deliver in session one are: Normalise reactions (discuss common psychological reactions in critical care and possible reasons); Encourage communication (help patients open up about worries and concerns), and Teach coping strategies, e.g. patients gathering more information and practising relaxation exercises (acronym NET). The second session comprises: Stress reactions (encourage further expression of worries and notice stressful thoughts); Explain stressful thinking (show how unrealistic fears can create extra stress), and Teach 'check out my fear', a technique to understand how accurate fears are (SET). The third session includes: Summarise and review (key messages from sessions and persisting issues); Action plan (personal planning to build on sessions and cope with challenges ahead) and Future Expectations (reinforce progress made and encourage realistic optimism for psychological and physical recovery (acronym SAFE).

For peer review only

Education package to deliver element two

The education package to train POPPI nurses to deliver element two includes a central three-day face-to-face training course with associated materials; a local debriefing and support visit, including a skills development assessment, and ongoing debriefing and support phone calls from trainers.

The course involves pre-course study (a psychological principles booklet, a manual for the three stress support sessions and access to the POPPI online training course) and a three-day programme, as follows:

Day one

- Understanding critical care patients' stress
- Learning the skills needed to deliver stress support sessions
- Overview of the stress support sessions
- Observing and practising session one

Day two

- More about unusual experiences in critical care
- Learning further stress support skills
- Observing and practising stress support session two

Day three

- Revision of key points from stress support session two
- Observing and practising stress support session three
- Using the patient booklet to create personal action plans
- Debriefing and support arrangements

Course materials include training folder with day 1-3 course handouts, word and visual summaries of each session, checklists and reflective notes for each session, stress thermometer tool, copies of patient booklet and DVD.

Debriefing and support

The first debriefing and support call should occur soon after (or during, if nurse requests) the three stress support sessions nurses deliver to their first POPPI patient. Subsequent calls are every two months, or on nurses' request. The focus of these calls is on enhancing nurses' skills and discussing patient cases. Emails can be exchanged between nurses and trainers about issues arising from stress support sessions at any time. Monthly teleconferences with POPPI nurses are facilitated by a trainer to encourage peer support.

A debriefing and support visit is made by an allocated trainer to offer the three POPPI nurses at one site further support, both individually and in a group. Later in the visit a skills development assessment is carried out for each nurse. Using a scenario, the nurse delivers session 2 to the trainer who plays the role of the patient. The trainer rates the nurse's skills using a checklist. The pass mark is 8/16. Nurses are required to pass the assessment, to continue delivering stress support sessions to further patients.

Element Three: Relaxation and recovery programme

In addition to stress support sessions, patients receive a "relaxation and recovery" programme (using a tablet computer app between sessions, and a DVD and patient booklet to take home after hospital) to help them to sleep and cope better with stress. Former intensive care patients' recovery stories are included to help normalise critical care experiences and reactions, and encourage hope and optimism for recovery. Contents also include audio or video files of a safe place visualisation, muscle relaxation and breathing exercises; a body scan and other mindfulness meditations; relaxing classical music from Bach to Vivaldi, calming modern ambient music; and restful nature sounds and scenes.

For peer review only

The programme was first developed as an application for tablet computers. The design of the app includes a green nature-scene background and large coloured buttons for patients to use for easy navigation between different sections. The tablet computers should be checked for approval by hospital infection control teams. DVDs include a shorter selection of relaxation exercises and music from the app, due to restricted space. However they include longer versions of the patient recovery stories, assuming longer concentration spans as patients get closer to hospital discharge or go home.

The "Getting well, staying well" booklet provides patients with information on making a good psychological recovery after a critical care stay. It builds on the support patients received during the stress support sessions, focusing on well-being and coping strategies. Contents include information about what to expect in the early days (leaving critical care, returning home; emotions after critical care, relationships), tips for psychological wellbeing; advice on coping with difficulties (worries, panic, low mood, memories); information on sources of psychological support; further information for family and friends and a personal action plan to promote well-being and recovery.

For peer review only

CONSORT 2010 checklist of information to include when reporting a pilot or feasibility trial*

Section/Topic	Item No	Checklist item	Reported on page No
**Title and abstract**			
	1a	Identification as a pilot or feasibility randomised trial in the title	n/a
	1b	Structured summary of pilot trial design, methods, results, and conclusions (for specific guidance see CONSORT abstract extension for pilot trials)	2
**Introduction**			
Background and objectives	2a	Scientific background and explanation of rationale for future definitive trial, and reasons for randomised pilot trial	Scientific rationale p3 (but not a randomised trial)
	2b	Specific objectives or research questions for pilot trial	p4, para 1
**Methods**			
Trial design	3a	Description of pilot trial design (such as parallel, factorial) including allocation ratio	4, 9/10
	3b	Important changes to methods after pilot trial commencement (such as eligibility criteria), with reasons	n/a
Participants	4a	Eligibility criteria for participants	9
	4b	Settings and locations where the data were collected	9
	4c	How participants were identified and consented	9
Interventions	5	The interventions for each group with sufficient details to allow replication, including how and when they were actually administered	5-8
Outcomes	6a	Completely defined prespecified assessments or measurements to address each pilot trial objective specified in 2b, including how and when they were assessed	9-10
	6b	Any changes to pilot trial assessments or measurements after the pilot trial commenced, with reasons	n/a
	6c	If applicable, prespecified criteria used to judge whether, or how, to proceed with future definitive trial	n/a
Sample size	7a	Rationale for numbers in the pilot trial	n/a
	7b	When applicable, explanation of any interim analyses and stopping guidelines	n/a
Randomisation:			
Sequence generation	8a	Method used to generate the random allocation sequence	n/a
	8b	Type of randomisation(s); details of any restriction (such as blocking and block size)	n/a

For peer review only

Allocation concealment mechanism	9	Mechanism used to implement the random allocation sequence (such as sequentially numbered containers), describing any steps taken to conceal the sequence until interventions were assigned	n/a
Implementation	10	Who generated the random allocation sequence, who enrolled participants, and who assigned participants to interventions	n/a
Blinding	11a	If done, who was blinded after assignment to interventions (for example, participants, care providers, those assessing outcomes) and how	n/a
	11b	If relevant, description of the similarity of interventions	n/a
Statistical methods	12	Methods used to address each pilot trial objective whether qualitative or quantitative	9/10
**Results**			
Participant flow (a diagram is strongly recommended)	13a	For each group, the numbers of participants who were approached and/or assessed for eligibility, randomly assigned, received intended treatment, and were assessed for each objective	Figures 2/3
	13b	For each group, losses and exclusions after randomisation, together with reasons	“
Recruitment	14a	Dates defining the periods of recruitment and follow-up	10/14
	14b	Why the pilot trial ended or was stopped	n/a
Baseline data	15	A table showing baseline demographic and clinical characteristics for each group	n/a
Numbers analysed	16	For each objective, number of participants (denominator) included in each analysis. If relevant, these numbers should be by randomised group	Figures 2/3
Outcomes and estimation	17	For each objective, results including expressions of uncertainty (such as 95% confidence interval) for any estimates. If relevant, these results should be by randomised group	Table 2 (pp12/13)
Ancillary analyses	18	Results of any other analyses performed that could be used to inform the future definitive trial	P14
Harms	19	All important harms or unintended effects in each group (for specific guidance see CONSORT for harms)	n/a
	19a	If relevant, other important unintended consequences	n/a
**Discussion**			
Limitations	20	Pilot trial limitations, addressing sources of potential bias and remaining uncertainty about feasibility	15
Generalisability	21	Generalisability (applicability) of pilot trial methods and findings to future definitive trial and other studies	n/a
Interpretation	22	Interpretation consistent with pilot trial objectives and findings, balancing potential benefits and harms, and considering other relevant evidence	p15
	22a	Implications for progression from pilot to future definitive trial, including any proposed amendments	p14
**Other information**			
Registration	23	Registration number for pilot trial and name of trial registry	p6
Protocol	24	Where the pilot trial protocol can be accessed, if available	

For peer review only

Funding	25	Sources of funding and other support (such as supply of drugs), role of funders	16
	26	Ethical approval or approval by research review committee, confirmed with reference number	9/16

Citation: Eldridge SM, Chan CL, Campbell MJ, Bond CM, Hopewell S, Thabane L, et al. CONSORT 2010 statement: extension to randomised pilot and feasibility trials. BMJ. 2016;355.
*We strongly recommend reading this statement in conjunction with the CONSORT 2010, extension to randomised pilot and feasibility trials, Explanation and Elaboration for important clarifications on all the items. If relevant, we also recommend reading CONSORT extensions for cluster randomised trials, non-inferiority and equivalence trials, non-pharmacological treatments, herbal interventions, and pragmatic trials. Additional extensions are forthcoming: for those and for up to date references relevant to this checklist, see www.consort-statement.org.

For peer review only

BMJ Open

Providing psychological support to people in critical care: development and feasibility study of a nurse-led intervention to prevent acute stress and long-term morbidity

Journal:	*BMJ Open*
Manuscript ID	bmjopen-2017-021083.R2
Article Type:	Research
Date Submitted by the Author:	15-May-2018
Complete List of Authors:	Wade, Dorothy; UCLH NHS Foundation Trust, Critical Care<br>Als, Nicole; UCLH NHS Foundation Trust, Critical Care<br>Bell, Vaughan; Maudesley Hospital, Psychological interventions clinic for outpatients with psychosis, Maudesley psychology centre<br>Brewin, Chris; University College London, Subdepartment of Clinical Health Psychology<br>D'Antoni, Donatella; King's College London, Institute of Pharmaceutical Science<br>Harrison, David; ICNARC,<br>Harvey, Mags; UCLH NHS Foundation Trust, Critical Care<br>Harvey, Sheila; ICNARC<br>Howell, David; UCLH NHS Foundation Trust, Critical Care<br>Mouncey, Paul; ICNARC, Clinical Trials Unit<br>Mythen, Monty; Institute of Sport Exercise and Health, 170 Tottenham Court Rd, University College London/University College London Hospitals, NIHR Biomedical Research Centre<br>Richards-Belle, Alvin; Intensive Care National Audit & Research Centre, Clinical Trials Unit<br>Smyth, Deborah; UCLH NHS Foundation Trust, Critical Care<br>Weinman, John; Kings College London, Institute of Pharmaceutical Sciences & Institute of Psychiatry<br>Welch, John; University College London Hospitals NHS Foundation Trust, Critical Care Department<br>Whitman, Chris; UCLH NHS Foundation Trust, Critical Care<br>Rowan, Kathryn; Intensive Care National Audit and Research Centre (ICNARC),
**Primary Subject Heading**:	Intensive care
Secondary Subject Heading:	Mental health
Keywords:	Adult intensive & critical care < ANAESTHETICS, Adult psychiatry < PSYCHIATRY, Depression & mood disorders < PSYCHIATRY, EDUCATION & TRAINING (see Medical Education & Training), Delirium & cognitive disorders < PSYCHIATRY

For peer review only

Title

Providing psychological support to people in critical care: development and feasibility study of a nurse-led intervention to prevent acute stress and long-term morbidity

Authors

Dorothy Wade, Health psychologist[1]

Nicole Als, Patient representative[1]

Vaughan Bell, Clinical psychologist[2]

Chris Brewin, Professor of clinical psychology[3]

Donatella D'Antoni, Psychology research assistant[4]

David Harrison, Head statistician[5]

Mags Harvey, Patient representative[1]

Sheila Harvey, Senior research fellow[5]

David Howell, Divisional clinical director[1]

Paul Mouncey, Senior trial manager[5]

Monty Mythen, Professor of anaesthesia and critical care[6]

Alvin Richards-Belle, Trial manager[5]

Deborah Smyth, Senior research nurse[1]

John Weinman, Professor of health psychology as applied to medicine[4]

John Welch, Consultant nurse, critical care and critical care outreach[1]

Chris Whitman, Patient representative[1]

Kathy Rowan, Director of scientific and strategic development/clinical trials unit director[5]

1. Critical care department, University College London Hospitals NHS Foundation Trust, 235 Euston Rd, London NW1 2BU
2. Psychological interventions clinic for outpatients with psychosis P079, Maudesley Psychology Centre, Maudesley Hospital, Denmark Hill, London SE58AZ
3. Research department of clinical, educational & health psychology, University College London, Gower Street, London WC1E 6BT
4. Institute of Pharmaceutical Science, King's College London, Franklin-Wilkins Building, 150 Stamford Street London SE1 9NH
5. Intensive Care National Audit & Research Centre, 24 High Holborn, London WC1V 6AZ
6. University College London/ University College London Hospitals NIHR Biomedical Research Centre c/o Institute of Sport Exercise and Health (ISEH) 170 Tottenham Court Road London, W1T 7HA

Correspondence to: Dorothy Wade, Critical Care department, T03, University College Hospital, 235 Euston Rd, London NW1 2BU. Email: Dorothy.Wade@nhs.net.

For peer review only - http://bmjopen.bmj.com/site/about/guidelines.xhtml

ABSTRACT

Objectives Adverse psychological outcomes, following stressful experiences in critical care, affect up to 50% of patients. We aimed to develop and test the feasibility of a psychological intervention to reduce acute stress and prevent future morbidity.

Design A mixed-methods intervention development study, using two stages of the UK Medical Research Council framework for developing and testing complex interventions. Stage one (development) involved identifying an evidence base for the intervention, developing a theoretical understanding of likely processes of change, and modelling change processes and outcomes. Stage two comprised two linked feasibility studies.

Setting Four UK general adult critical care units.

Participants Stage one: Former and current patients; psychology, nursing, education experts. Stage two: Current patients, staff.

Outcomes Feasibility and acceptability to staff and patients of content and delivery of a psychological intervention, using quantitative and qualitative data. Estimated recruitment and retention rates for a clinical trial.

Results Building on prior work, we standardised an intervention for nurses to provide psychological support to people in intensive care (POPPI). We devised courses and materials to train staff to create a therapeutic environment, to identify patients with acute stress, and to deliver three stress support sessions and a relaxation and recovery programme to them. 127 awake, orientated patients took part in an intervention feasibility study in two hospitals. Patient and staff data indicated the complex intervention was feasible and acceptable. Feedback was used to refine the intervention. 86 (54%) different patients entered a separate trial procedures study in two other hospitals, of which 66 (80% of surviving patients) completed questionnaires on post-traumatic stress, depression and health five months after recruitment.

Conclusion The 'POPPI' psychological intervention to reduce acute patient stress in critical care and prevent future psychological morbidity was feasible and acceptable. It was refined for evaluation in a cluster-randomised clinical trial.

Trial registration number: ISRCTN61088114

Strengths and limitations of this study

- The complex intervention built on extensive preparatory work investigating psychological risk factors, outcomes and interventions in critical care
- The intervention was rigorously developed using a widely-accepted framework, under expert supervision
- The feasibility of trial procedures and delivery of the intervention were thoroughly tested in two separate studies
- There was limited patient feedback on the intervention
- No efficacy data were collected in the feasibility studies

For peer review only - http://bmjopen.bmj.com/site/about/guidelines.xhtml

INTRODUCTION

More than 170,000 patients are admitted to adult, general critical care units in the National Health Service (https://www.icnarc.org/DataServices/Attachments/Download/a30185e2-0e19-e711-80e6-1402ec3fcd79) in England, Wales and Northern Ireland each year. Although medical advances mean that increasing numbers of people survive, there is evidence that many critical care patients develop both acute stress and long-term psychological morbidity.[1] Critical care units are stressful places, where patients suffer symptoms such as pain, thirst, nausea, fatigue, and disorientation associated with critical illness, invasive medical procedures and side-effects of potent drugs.[2, 3] Being attached to machines, tubes, masks and other equipment in a busy, noisy ward, often without access to daylight, windows or clocks, leads to sensory disruption and sleep deprivation. Patients are often isolated, unable to communicate, but aware of other people's suffering or death.[4] Unsurprisingly, 45%-80% of critical care patients experience acute stress in the form of panic, fear, depressed mood, anger, as well as hallucinations or delusions. They may have flashbacks of frightening ICU experiences including hallucinations or delusions after leaving hospital.[5-7] Studies suggest that up to 50% of patients or more experience symptoms consistent with post-traumatic stress disorder (PTSD), anxiety or depression following critical care.[8-12]

The UK National Institute for Health and Care Excellence (NICE) guideline on rehabilitation from critical illness recommends that highly stressed patients should be identified and offered psychological support as part of a recovery plan. Quality standards have recently been implemented to strengthen these requirements.[13] Most interventions to reduce psychological morbidity have been implemented in the months following discharge from critical care and hospital. They include follow-up clinics, rehabilitation services, patient diaries and nurse-led psychological recovery sessions.[14-17] Diaries of patients' admissions, written mainly by staff and given to patients one to three months post-critical care discharge, reduced acute-onset PTSD at three months in one randomised clinical trial (RCT).[18] Training critical care survivors in mindfulness and coping skills training was found to be feasible in small studies, but in an RCT coping skills training after hospital did not reduce psychological distress at six months.[19] Overall there is a lack of clear evidence that critical care follow-up services reduce long-term distress.[15-16]

Given that patients who suffer acute stress, including hallucinations and delusions, in the critical care unit have a higher risk of future psychological morbidity,[20,21] psychological interventions commenced during critical care admissions could be effective. But due to a lack of evidence about what helps, little is currently done to alleviate stress in critical care. Sedatives or antipsychotics may be prescribed to reduce stress and agitation, but they have potentially harmful side-effects, both physical and psychological. Evidence exists that psychosocial interventions such as music therapy, massage, reflexology, relaxation and clinical psychology sessions delivered in critical care, can reduce patients' stress.[22-24] But more robust research is urgently needed.

We hypothesised that techniques from psychological interventions used in other populations, such as people with psychosis or trauma,[25-28] could be effective for patients still in the critical care unit. At an early development stage, a psychological approach used to reduce anxiety, distress from hallucinations and

For peer review only - http://bmjopen.bmj.com/site/about/guidelines.xhtml

delusions, and traumatic stress, was adapted for critical care, and used by a psychologist in one unit (electronic supplement one). As psychologists are a scarce resource in hospitals, we proposed that, given extra training, critical care nurses have the necessary experience and motivation to provide psychological support to patients. We developed the intervention using the UK's Medical Research Council framework to guide researchers in creating evidence-based, theoretically sound and robustly evaluated complex interventions.[29] This paper covers stage one (development) and stage two (feasibility/piloting) of the MRC framework (see figure one). Stage three (evaluation of clinical and cost-effectiveness in a cluster-randomised clinical trial, cRCT) will be reported separately.

The objectives of this mixed-methods intervention development study were to: standardise a nurse-led psychological intervention to support patients in critical care; to create an associated staff education package; to test the feasibility and acceptability of the intervention; to test trial procedures, and to refine the intervention for evaluation in a trial (https://www.journalslibrary.nihr.ac.uk/programmes/hsdr/1264124/#/).

DEVELOPMENT (STAGE ONE)

Methods

Stage one of the MRC framework includes three key tasks: Identifying the evidence base for an intervention; developing a theoretical understanding of the likely processes of change; and modelling process to progressively refine the intervention. We reviewed the evidence base on critical care-related psychological risk factors, outcomes and interventions.[1,30] Two authors conducted a new systematic review of psychological interventions in critical care.[25] We also conducted primary research studies[7,31-33] to develop an understanding of the determinants of psychological distress in critical care and the likely mechanisms of change involved in reducing stress (see electronic supplement one).

The modelling process involved testing and revising of the complex intervention, by psychologists, nurses, and patients, supervised by an expert psychological advisory group (EPAG) including clinicians, an educationalist and specialists in psychological interventions for psychosis and trauma. This resulted in the nurse-led POPPI (provision of psychological support to people in intensive care) intervention, comprising three inter-connected elements: Creating a therapeutic environment for all patients; delivering stress support sessions for patients with acute stress, and provision of a relaxation and recovery programme. Acute stress was defined by scores >7 (0-20) on the Intensive care Psychological Assessment Tool (IPAT) previously validated by our group.[34] An education package was created to train staff to deliver the intervention, with help from the EPAG, educational and web designers and a medical film-maker.

Patient and public involvement

A patients and family advisory group, diverse in age, gender, culture and critical care experiences, was set up at the start of the intervention development phase at University College Hospital, London. Twenty members of the patient group contributed to the early conception of the POPPI study and then to every stage of

For peer review only - http://bmjopen.bmj.com/site/about/guidelines.xhtml

intervention development. Three patients were members of the expert psychological advisory group. Five were filmed narrating their critical care experiences, for use in the nurse training courses and to be viewed by current patients as part of the relaxation and recovery programme. The whole group were sent POPPI patient materials as they were devised, for comment and revision.

Results

Our review of the evidence base [1,30] pointed to likely prevalence rates of 40% clinical anxiety symptoms, 30% depression, and 20% post-traumatic stress among survivors. There was increasing evidence that acute stress, as well as early intrusive or delusional memories, were important risk factors for psychological morbidity.[7, 20-21] Twenty-three studies were identified In our systematic review of non-pharmacological interventions to reduce critical-care related stress,[35] with 12 showing beneficial effect, although quality and quantity of evidence was limited. However music, relaxation, visualisation and psychotherapeutic approaches showed promise as elements of a complex psychological intervention.

Our primary research to further understanding of the determinants and proposed mechanisms of change involved consulting hundreds of patients, relatives and nurses using questionnaires, interviews or focus groups.[7,31-33] Patients expressed the need for understanding and support to deal with anxiety, panic, hallucinations, delusions and flashbacks, as well as the frightening environment in the critical care unit. They wanted to trust staff, feel safe, be listened to and get more information (e.g. how drugs might cause hallucinations). They said nurses' ability to communicate with frightened or delirious patients was variable, but understood that caring for stressed, delirious patients was difficult, and suggested that training could improve communication and empower nurses to deliver one-to-one psychological support. Nurses said they were motivated to improve patients' psychological well-being and make the environment less stressful, but many thought they lacked the necessary knowledge and skills, and were frightened of "making things worse." They agreed with patients that training would help to increase staff psychological awareness and skills, along with a protocol to help staff identify patients most in need and to deliver one-to-one psychological support. The conclusion of the consultation was that all staff should be trained to reduce general stress and improve communication in critical care, but the most stressed patients would benefit from focused one-to-one psychological support.

For peer review only

As well as adapting techniques from psychological interventions for psychosis and trauma for the needs of critical care patients[25-28], we drew on well-established work on stress, health and coping,[36] as well as ideas to inform training health staff in new behaviours,[37] including therapeutic communication and engagement with patients, especially those who were distressed by on-going hallucinations and / or delusions.[38,39] We took into account the need for clinical supervision of non-expert staff delivering psychological support.[40] Finally, we standardised the three inter-connected elements of the complex intervention (see table one) and, guided by the EPAG educationalist, created an education package with associated materials to deliver it. The resulting POPPI intervention is described below. For more details of each element, see electronic supplement two.

For peer review only - http://bmjopen.bmj.com/site/about/guidelines.xhtml

In each unit three POPPI nurses were charged with ensuring that the Intensive care Psychological Assessment Tool (IPAT) was used routinely, encouraging staff behaviour change and overseeing delivery of the whole intervention. The first element of the intervention was for critical care staff to create a more therapeutic environment in their unit by improving communication with distressed patients and reducing stressors such as noise, unnatural light, insomnia, pain, and psychoactive drug effects. This was primarily delivered via online training (see below for course details). The POPPI nurse role included encouraging all staff to complete online training and reinforcing key learning objectives through ongoing education and bedside teaching.

The second element consisted of three 30-minute stress support sessions delivered by POPPI nurses to acutely stressed patients (scores>7 on the IPAT), ideally starting the sessions in the critical care unit within 48 hours of IPAT screening and completing the three within a week (in the unit or on other wards). The three stress support sessions were based on psychological techniques evaluated in other populations and adapted for critical care patients. Techniques from psychological interventions for psychosis included establishing a collaborative relationship focused on reducing distress; managing patient concerns based on hallucinations and delusions; psychological education to reduce distressing interpretations of unusual experiences, reduce stigma, and encourage open communication; and provision of active coping strategies. Interventions were adapted to take account of critical care patients' likely physical, emotional or cognitive fragility.

Stress support sessions also included psychological techniques from trauma-related interventions to help people to identify unhelpful thoughts, interpretations and coping styles, and to think about their experiences in a less upsetting way. However some psychological interventions for trauma are not suitable for critical care patients. For example during stress support sessions, patients were encouraged to express their thoughts and feelings about their critical care experiences if they wish, but not to undertake 'reliving' their critical care stay, particularly as their trauma may be continuing.

The main objectives of the stress support sessions were for nurses to develop a trusting relationship with patients, so patients could discuss concerns which they might feel embarrassed or worried about communicating, and to reduce emotional distress. Stress support session one involved discussing and normalising common psychological reactions in critical care, helping patients to open up about worries, and exploring coping strategies. In the second stress support session, patients were encouraged to open up more fully about their fears; to identify highly stressful thoughts, and to learn how to find out if their worst fears were realistic. The third session began with summarising key messages and reviewing remaining problems. Nurses then helped patients to make a personal plan to cope with challenges ahead, to build realistic optimism about recovery and plan where to seek help if problems arise in future. Patients were asked to rate their stress levels on a "stress thermometer" (a simple tool commonly used by psychotherapists to rate stress levels during therapy on a scale of 0-10) at the beginning and end of each stress support session, to help nurses monitor how patients were feeling as the sessions progressed.

For peer review only - http://bmjopen.bmj.com/site/about/guidelines.xhtml

The third element of the complex intervention was a relaxation and recovery programme designed to help people practise new coping strategies, to provide meaningful activity and distraction, to and to learn from other patients' experiences, between stress support sessions. The POPPI app with calming music, relaxation, meditation, nature sounds/videos and patient recovery stories was loaded onto tablet computers loaned to patients receiving stress support sessions. DVDs containing similar materials to the app, and a self-help booklet "Getting well, staying well" were given to patients to keep and take home.

For peer review only

For peer review only - http://bmjopen.bmj.com/site/about/guidelines.xhtml

Table one: The three elements of the POPPI intervention to reduce stress in critical care patients

Patient interventions	Who receives?	Where?	Proposed mechanisms of change	Training methods	Who is trained?
Element one: Creating a therapeutic environment in critical care	All critical care patients	In the critical care unit	Raised staff awareness of patients' stress and psychological morbidity.<br>Staff motivated to reduce stressors in the unit.<br>Communication skills training for staff.<br>Emphasis on positive recovery messages.	An online training course<br><br>Promotional materials and local education sessions to reinforce key messages	All clinical critical care staff
Element two: Three stress support sessions for patients screened as acutely stressed	Patients screened by Intensive care psychological assessment tool (IPAT)[22] as acutely stressed (scores >7, range 0-20)	In the critical care unit, on wards following discharge from critical care	Help patients to express fears or concerns, and process traumatic memories (S1/2).<br>Reduce catastrophic misinterpretations by a) normalising stressful thoughts and feelings, and b) testing the reality of fears or concerns (S1/2)<br>Promote hope and motivation for recovery (S3)	A three-day face-to-face training course for 'POPPI' nurses to learn to deliver stress support sessions and coach patients in using the relaxation and recovery programme<br><br>Provision of educational materials (pre-course theory booklet, manual and training folder) for POPPI nurses, and ongoing debriefing and support (clinical supervision) from trainers	Three "POPPI" nurses per critical care unit, selected by units with reference to suitability criteria
Element three: Relaxation and recovery programme on app, DVD and booklet (music, relaxation, meditation, patient recovery videos and self-help information)	Patients screened as acutely stressed and receiving stress support sessions	In the critical care unit, on wards (via tablet computer) and at home (via DVD and self-help booklet)	Provide distraction and meaningful activity.<br>Reduce stress by teaching patients relaxation and coping strategies (in S1/2)<br>Normalise emotional reactions, and promote optimism by providing former patients' recovery videos (In S1/2)<br>Promote recovery by providing information and helping to create a personal plan for future well-being (S3, DVD, booklet)		

For peer review only - http://bmjopen.bmj.com/site/about/guidelines.xhtml

An education package was developed to train staff in each element of the intervention. We adopted a 'blended learning' approach, combining online training and face-to-face teaching. Interactive and engaging online training courses are recommended by educationalists for the provision of knowledge to a large number of staff, while face-to-face training is suitable for adults acquiring new skills by practising and receiving feedback.[41] We evaluated training by measuring nurses' reaction to the courses, learning, and self-confidence in psychological skills.[42] The online training course to deliver element one, had four aims: to increase knowledge and understanding of the psychological impact of critical care; to learn to reduce stressors in the environment; to improve staff communication skills; and promote realistic hope for patients' recovery. It was co-designed with education experts as an interactive course using videos, colourful graphics, and quizzes. On completing the course, staff took an online knowledge test and received certificates if they passed.

Additionally a central face-to-face course was devised to train POPPI nurses to deliver all elements of the complex intervention. During the three-day course, psychological principles were taught and all three elements of the intervention were covered. A significant amount of time was devoted to skills practice in delivering stress support sessions, with the training team (a psychologist, nurses and patient representatives) observing and offering feedback. An additional day of feedback and assessment was held after POPPI nurses returned to their units and delivered sessions to at least one patient each. The day included nurse focus groups on their experience of delivering stress support sessions, and a competence assessment, in which nurses delivered stress support session two to a simulated patient (actor), observed by trainers who completed a checklist. Thereafter nurses had clinical supervision with trainers, during regular "debriefing and support" phone calls.

Associated training materials were developed, including an intervention manual for POPPI nurse, a set of slides for the three-day training course; and a training folder consisting of course hand-outs, patient scenarios, stress thermometers, summaries and checklists of each stress support session, and reflective note templates.

Key psychological outcomes were identified as PTSD and depression, as the most serious common psychological outcomes of critical care. Research in identifying modifiable critical care risk factors is more extensive for PTSD than for depression and anxiety, therefore PTSD was chosen as the primary outcome of the full trial.

FEASIBILITY AND PILOTING (STAGE TWO)

Methods

Once development of the complex intervention was completed, two separate feasibility studies were conducted , following a favourable ethical opinion from the National Research Ethics Service Committee South Central - Oxford B on 23 April 2014 (14/SC/0149). An Intervention feasibility study was held in two English adult general critical care units (UCLH and Watford General Hospital) and a different trial procedures feasibility study was held in two other hospitals(Bristol Royal Infirmary and Medway Maritime Hospital).

For peer review only - http://bmjopen.bmj.com/site/about/guidelines.xhtml

Intervention feasibility study

The aims of this study were to test the feasibility and acceptability of content and delivery of the complex intervention, and to get feedback from patients and staff, to help refine the intervention prior to full evaluation. No follow-up questionnaires were sent to patients in this study. Each hospital was asked to send three "POPPI" nurses to attend the central three-day face-to-face course to learn about content and delivery of the intervention. Once they returned to their hospitals, all clinical staff in their units were asked to complete the online training course on creating a therapeutic environment, and patient recruitment began. POPPI nurses also attended an additional day of feedback and assessment once each had conducted stress support sessions with at least one patient.

Inclusion criteria for patients were: Age 18 or greater; receipt of Level 2 or 3 care for 48 hours or more; Richmond Agitation Sedation Scale (RASS)[43] score between +1 and -1; English-speaking and ability to communicate orally; no pre-existing cognitive impairment, psychotic illnesses or chronic PTSD; Glasgow Coma Scale (GCS) score of 15; not terminally ill or receiving end-of-life care. Patients were provided with written and verbal information as part of the informed consent process. Consenting patients were screened for acute stress (>7 on the IPAT). Stress support sessions and a relaxation and recovery programme were delivered to acutely stressed patients by POPPI nurses.

Feasibility and acceptability of content and delivery of the intervention were assessed using quantitative and qualitative data from nurses and patients(see table two). Quantitative data included delivery rates of each element of the intervention, as well as scores/ratings from patient satisfaction questionnaires and staff training evaluation questionnaires (on satisfaction, learning and self-efficacy). Staff satisfaction and learning questionnaires were completed at the end of the online training course and the three-day face-to-face course. POPPI nurses also completed self-efficacy (confidence in psychological skills) questionnaires pre- and post- the three-day course, and on the additional feedback and assessment day, when a competence assessment was also carried out with a trainer for each nurse. Qualitative nurse data were collated from focus groups on the additional training day and at the end of the study, nurse debriefing sessions with trainers and from case report forms (CRFs). Once the intervention was embedded in the intervention sites, patients who received the stress support sessions and relaxation and recovery programme completed satisfaction questionnaires. Qualitative patient data came from free-text sections on the satisfaction questionnaires and nurse-reported patient comments in the CRF. The above data were used to refine the intervention, where relevant.

Trial procedures feasibility study

The aim of this study was to determine recruitment and retention rates for a full trial. Patient eligibility criteria were the same as for the intervention feasibility study, and consent was obtained. The intervention was not delivered in this study. At five months post-recruitment, patients were sent follow-up psychological and health questionnaires proposed for the full trial. These included the PTSD Symptom Severity Scale – Self Report (PSS-SR)[44] and the Centre for Epidemiologic Studies Depression Scale (short form) (CESD-10).[45]

For peer review only - http://bmjopen.bmj.com/site/about/guidelines.xhtml

Patient and public involvement

The patient group continued their involvement with POPPI throughout the feasibility phase. Patients attended the nurse three-day training course, both to tell their own critical care stories and to observe and give feedback during nurses' skills practice to deliver the stress support sessions. They helped to interpret results of the feasibility study, and would go on to play a key role in the full POPPI trial. Additionally, patient participants in the studies were given a link to the POPPI website so they would be able to read results of the studies.

Results

Intervention feasibility study

Delivery of the intervention

Ten POPPI nurses (100%) from the two hospitals completed a central three-day face-to-face training course (due to personnel issues, this included training extra nurses at one site). When they returned to their hospitals, online training became available for all staff, and patient recruitment was opened for 5.5 months. 283 (84%) staff in the two critical care units took the online training course, surpassing the target rate of 80%, with 277 (98%) passing the end of course assessment. 127 patients were recruited to the study, all were screened with the IPAT, and 51 (40%) were identified as acutely stressed (see figure two). 44 stressed patients (86%) received stress support sessions, with 39 (77%) receiving two or three sessions and five having one session only. Seven patients (14%) had no sessions because they declined, were discharged home early or deteriorated clinically. Median duration of sessions one to three was 35, 30 and 30 minutes respectively. 40 patients (90%) received a tablet computer, and 27 (61%) were given a DVD and patient booklet to keep.

Content of the intervention

All nurse and patient feasibility questionnaires had scales of 0-5 per item, with 4 or 5 defined by us as "good" scores or ratings. Staff gave the online training course good ratings for: Stimulating (73%, 189/260); useful (86%, 223/260) and well-designed (84%, 216/257). All ten POPPI nurses rated the face-to-face course as good (stimulating, useful, well-conducted and motivating), with nine (90%) rating relevance to their new role as good. There was a large increase in nurse self-efficacy, from 30% good scores (21/70 across seven items) pre-course, to 73% good scores (51/70) post-course. The same proportion of 73% good scores was maintained at follow-up. For nurse learning, 87% (69/79) of post-course scores on eight items were good.

Once the intervention was embedded, fifteen patients completed a satisfaction questionnaire. Satisfaction with stress support sessions was rated as good by 14 (93%) patients. Fifteen patients (100%) gave good ratings for nurses understanding them and normalising their fears. Slightly fewer patients thought their stressful thoughts (13, 87%) or feelings (12, 80%) were reduced by the sessions. Twelve (80%) rated the number and duration of sessions as "just right". Satisfaction with the relaxation and recovery programme was somewhat less than for the stress support sessions. Ten of 14 (71%) rated contents of the tablet app as good, while 10 of 15 (67%) thought that they learned good coping strategies from the programme. Stress, rated on the stress

For peer review only - http://bmjopen.bmj.com/site/about/guidelines.xhtml

thermometer (0-10), was reduced by median 3 points (IQ range 1-5 points) from the start of session one to the end of session three among the 25 patients who received all three sessions.

Quantitative feasibility and acceptability data are presented fully in table two. Qualitative data on the three elements are also summarised in the table, but specifics, both positive and negative, are described in more detail as follows. Nurses reported that the online training course had raised awareness, changed staff thinking and led to a better environment in their units. However, they said design improvements, more practical advice and summaries of key messages were needed. Delivery of element one was challenging, as POPPI nurses were too busy to support other staff in creating a therapeutic environment. It was suggested this responsibility should be shared by the wider critical care team, and further educational/ promotional materials were needed.

Nurse feedback on content of the second element was that stress support sessions were welcomed by patients and rewarding for nurses. Challenges included ensuring patients understood the sessions and knowing how to handle very serious issues that might be raised. Delivery of stress support sessions could be difficult to fit into heavy clinical workloads, especially if patients postponed sessions due to fatigue or sickness.

Comments on the three-day course included positive ones such as “best course ever”; “feel privileged” to be part of it. However some nurses said they found it hard work and tiring. They suggested it would be less intense if psychological theory was delivered to be studied in advance, rather than presented during the course, and that skills practice should involve actors (instead of fellow trainees) to play patients. They found the POPPI nurse training manual invaluable, but suggested some re-structuring and clarification in the presentation of stress support sessions, with better linkage to appendices. All nurses found the competence assessment on the additional training day stressful. Most said they welcomed the regular debriefing and support calls with trainers, finding them useful for reflection and building greater confidence.

Patient feedback on the stress support sessions was mainly positive, and included “every hospital should have it”, “it seemed a life saver”, “proves that I am normal but under the influence of morphine” and “I hope that POPPI will eventually be used in all ICUs. Thank you for the support.” Criticisms included one patient saying that the critical care environment had not been improved in response to his concerns, and some patients wanted more than three stress support sessions. Patients also responded well to the relaxation and recovery programme. Some patients preferred patient stories, some relaxation exercises, some meditation and some calming music. However some found the tablet computers confusing and difficult to use, due to unfamiliarity with the technology, or reduced dexterity. Unfortunately the tablets used in the intervention feasibility study did not have good touch-sensitivity. Some patients were disappointed that the DVDs did not include the calming music (due to copyright issues).

For peer review only - http://bmjopen.bmj.com/site/about/guidelines.xhtml

Table two: Feasibility, acceptability and refining of the three elements of the psychological intervention

Elements of intervention	Content/ delivery of element	Feasibility and acceptability indicators – quantitative and qualitative	Feasibility and acceptability results	Refinement of the intervention post-feasibility study
Element one: Creating a therapeutic environment in critical care	Content of online training course	Training course ratings by all staff (% with 4 or 5(0-5) or "good")	Stimulating 73%; Useful 86%; Well-designed 84%; Right length 80% (n=260, but missing data for some items)	Online training course shortened and made more visually appealing, with more practical advice on reducing stressors in critical care units and clearer presentation of key messages
		Favourite parts of course – all staff	Factual information 42%; Patient stories 35%; Communication videos 13%; Tests 10% (n=260)	
		Nurse qualitative feedback *	Staff positive, suggested minor improvements	
	Delivery of online training and creating a therapeutic environment	Staff taking course (target: 80%)	283 (84%)	Provision of training, display materials and slide-sets for seminars/ workshops, for local education teams to support and motivate staff in creating a therapeutic environment
		Staff passing final test (score >80%)	277 (98%)	
		Staff learning scores (% with 4 or 5(0-5) or "good")	74% (n=259)	
		Nurse qualitative feedback	POPPI nurses lacked time, due to workload, to support staff in creating therapeutic environment	
Element two: Three stress support sessions for patients screened as acutely stressed	Content of screening	Previously validated[34]		
	Delivery of screening	Consenting patients screened	127 (100%)	
		Screened as acutely stressed	51 (40%)	
	Content of stress support sessions	Median (IQR) difference in patient stress thermometer scores (0 - 10)	Median difference from start session 1 to end session 3 was -3.0 (-5.0, -1.0) (n=25 patients who had all 3 sessions)	Content of stress support sessions clarified for POPPI nurses and patients by reorganising sessions from five components each, into three common components in all sessions and three individual components per session. Manual became more tightly focused on stress support sessions (rather than the whole intervention) with clearer signposting to and between sections
		Patient satisfaction with stress support sessions (% with 4 or 5(0-5) or "good")	Overall 93%; Helped express fears 93%; Nurse understanding 100%; Nurse normalised fears 100%; Fewer stressful thoughts 87%; fewer stressful feelings 80%; number/duration of sessions 80% (n=15, missing data some items)	
		Patient qualitative feedback	Stress support from nurses was very helpful	
		Nurse qualitative feedback	Rewarding, but challenging to explain to patients	
	Delivery of stress support sessions	Number of stress support sessions patients had	25 (49%) had three sessions; 14 (28%) had two sessions; 5 (10%) one session; 7 (14%) had none	Ensure buy-in and support for POPPI nurses from clinical, education and research staff from the start, making it a team effort. If hospital discharge is near, sessions two and three can and should be delivered together
		Median duration of sessions (minutes = m)	Session one: 35m, Session 2: 30m, Session 3: 30m	
		Nurse qualitative feedback	Hard to fit sessions in with ordinary duties, especially if patients postponed. Patients missed session three if they were discharged home early	

For peer review only - http://bmjopen.bmj.com/site/about/guidelines.xhtml

	Content of POPPI nurse face-to-face training course - three days and a feedback/ assessment day	Nurse feedback – post-course questionnaire (% with 4 or 5(0-5) or "good")<br><br>Nurse self-efficacy (in delivering psychological support) – pre-/post-course and feedback day questionnaires. (% of 4 or 5(0-5) or "good" ratings of items<br><br>Nurse course learning – an 8-item post-course knowledge questionnaire - (% of 4 or 5(0-5) or "good" scores for items)<br><br>Trainer assessment of nurse competence using six-item checklist (scores 0-12; pass =6)<br><br>Nurse qualitative feedback	Stimulating 100%; Useful 100%; Relevant 90%<br>Well conducted 100%; Motivating 100% (n=10)<br><br>Big increase in self-efficacy from pre- to post-course; maintained at follow-up. 30% of scores (21/70) across items were good pre-course; 73% were good scores (51/70) post-course and on feedback day<br><br>87% of scores (69/79) were "good" for learning on:acute stress; screening; aims of stress support sessions, normalising, psycho-education, communication style, stressful thinking, checking out fears, coping<br><br>100% passed (9 on first attempt, 1 on second)<br>Median (IQR) score of passes 9 (9, 10)<br><br>Course highly valued but tiring. Skills practice stressful. Competence assessment on follow-up day stressful	To reduce burden and increase self-efficacy, the three-day course became more focused on stress support sessions, particularly session two, seen as the most difficult session to deliver. More emphasis on skills practice, with actors (not fellow trainees) playing patients. Wider spectrum of patient scenarios used.<br><br>Assessment to be re-framed as "skills development" and carried out one-to-one, with a trainer playing the patient
	Delivery of three-day training course	Number of required trainees attending	10 (100%)**	Some modules dropped from course; pre-course booklet on psychological principles provided
	Content: Debriefing support by trainers	Nurse qualitative feedback	Debriefing calls useful for reflection, confidence<br>Assessment should be part of ongoing support	Assessment to be one-to-one confirmation of skills (as above), as part of ongoing debriefing and support
	Delivery: Debriefing and support	Nurse qualitative feedback	Nurse debriefing and support should start earlier	First debriefing call after first POPPI patient
Element three: Relaxation and recovery programme on App, DVD and booklet (music, relaxation, meditation, patient recovery videos, self-help information)	Content of relaxation and recovery programme	Patient satisfaction with content of programme - (% with 4 or 5(0-5) or "good")<br><br>Nurse-reported qualitative patient feedback	Content on tablet computer app 71%; Useful post-ICU coping ideas 67% (15 patients, some missing data)<br><br>Varied preferences; relaxation, meditation, nature sounds, patient stories or calming music. Some disappointed not to find calming classical music on DVD.	Content and design of the relaxation app were improved. Balance of contents of DVD improved, and calming classical music tracks added. Layout and readability of the patient booklet improved.
	Delivery of relaxation and recovery programme	Patients receiving tablet in session one<br><br>Patients receiving DVD or booklet<br><br>Nurse-reported qualitative patient feedback	40 (90%)<br><br>27 (61%)<br><br>Some liked tablet, others found it hard to use , some preferred DVD or patient booklet	Usability of the relaxation app was improved, to make it easier for the less dextrous. Higher- spec tablets, including better touch sensitivity, identified for use in future trial<br><br>DVD and booklet to be provided in session two, so more patients would receive them (many missed receiving session three due to being sick or discharged from hospital)

** All nurse and patient qualitative data described on p.11. **Four additional nurses were trained at one site due to personnel issues*

For peer review only - http://bmjopen.bmj.com/site/about/guidelines.xhtml

Refinement of the complex intervention

We refined the complex intervention based on key points emerging from the feasibility data (see table two, last column). To refine element one, we made the online training course shorter, more attractive and clearer, and included more practical advice and emphasis on key messages. We created new slide-sets and display materials for units to reinforce the key messages to their staff. We decided that the wider critical care team should be involved in creating the therapeutic environment, so that POPPI nurses could focus on delivering stress support sessions to patients.

To improve element two, the three-day face-to-face POPPI nurse course was more tightly focused on the stress support sessions, with more emphasis on skills practice, and actors playing patients, to reduce the burden on trainees. Some modules, such as a booklet on psychological principles, were provided to POPPI nurses in advance, to reduce the intensity of the course. The nurse competence assessment was reframed as a skills development assessment and carried out less formally as part of ongoing one-to-one debriefing and support sessions with a trainer. The manual was made simpler and clearer, to improve nurse and patient understanding of the components of the stress support sessions. As many patients in the feasibility study did not receive the third support session due to early hospital discharge, it was decided that sessions two and three could and should be delivered together in those circumstances, if patients agreed and were well enough.

To refine element three, the content, design and usability of the relaxation app was improved, and tablets with better touch sensitivity were identified for use in the future trial. Small improvements were made to the design and content of the DVD and patient booklet, and it was decided they would be given to patients in session two rather than session three, so that more patients would receive that element of the intervention, even if they were due to leave hospital after session two.

Trial procedures feasibility (study two)

There was a two-month recruitment period. Of 160 eligible patients in the two sites, 86 (53.8%) provided consent to take part (see figure three). Of these, nine (10.5%) had died by 5 months, but 62 (80.5%) completed the 5-month follow-up questionnaire. Overall completeness of the primary outcome measure (the PSS-SR[44]) was very good. For a total of 1054 fields, only 24 (2.3%) had missing data.

DISCUSSION

The objectives of the POPPI intervention development study were met: we developed and standardised a nurse-led psychological intervention to reduce patients' acute stress in critical care; we created a staff package to deliver it; we tested feasibility of the intervention and trial procedures, and refined the intervention. The intervention had three inter-connected elements: Creating a therapeutic environment; stress support sessions delivered by selected, trained nurses to acutely stressed patients, and a relaxation and recovery programme for acutely stressed patients. To support delivery of the intervention, we devised and created a relaxation and recovery App, DVD and booklet for patients; an online training course for all critical care staff; and a training

For peer review only - http://bmjopen.bmj.com/site/about/guidelines.xhtml

package for POPPI nurses, including a manual of the three stress support sessions, a three-day face-to-face course with training folder, and a debriefing and support programme.

An intervention feasibility study showed that delivery and content of the complex intervention were feasible and acceptable for patients and staff, and led to a refined version of the intervention, ready for evaluation in a cluster-randomised clinical trial (cRCT). A separate trial procedures feasibility study demonstrated that the necessary recruitment and retention rates for a cRCT could be achieved.

Strengths of this study: The intervention builds on extensive prior work investigating the psychological impact of critical care and it was developed using a rigorous framework[29] with input from a large patient advisory group and supervision from an expert team. The detailed development work resulted in a novel nurse-led preventative psychological intervention to improve psychological outcomes. Importantly, the intervention has three-interconnected elements designed to: reduce stress in the environment and improve staff-patient communication; to provide targeted support for the most stressed patients; and to provide stress relief and meaningful activity for patients. Feasibility of both delivery of the intervention and trial procedures were thoroughly tested in two studies, involving more than 200 patients and 280 staff. The psychological intervention was confirmed to be feasible and acceptable for patients and staff. Formal evaluation of the intervention in the POPPI cluster randomised clinical trial is underway.

Limitations were that the intervention was piloted in only two sites, and limited patient feedback was gathered. No efficacy data were collected during the feasibility studies.

The POPPI intervention addresses evidence that critical care patients suffer acute and long-term stress.[7-10] Despite increasing recognition[13] that interventions to reduce critical care stress are needed, little is known about what would be most effective. Studies of early interventions such as music, relaxation techniques and psychological support in critical care showed promise.[23,24] POPPI is the first complex psychological intervention designed to be delivered early, during the critical care admission, to prevent PTSD and other long-term morbidity.

Conclusion

The POPPI psychological intervention to reduce stress and prevent long-term morbidity for critical care patients was feasible, acceptable and ready for evaluation in a cluster-randomised clinical trial.

Supplementary material: Click here to view

Contributors

Early conceptual and development work for the POPPI intervention was carried out by DW, JWei, DHo, MM, DS, JW, NA, MH, CW and CB at University College London Hospitals. Further development of the intervention and design of the feasibility phase of the POPPI study were carried out with KR, SH, DHa, PM, ARB, DD'A and

For peer review only - http://bmjopen.bmj.com/site/about/guidelines.xhtml

VB. DW drafted the initial manuscript with input from KR. All authors revised the manuscript critically and gave their approval of the final version. DW is the guarantor.

Acknowledgements

Daniel Freeman (chair of the education and psychology advisory group), Dane Goodsman (clinical educationalist), Rob Beer (online training designer), Daniel Zanitchkhah (app designer), Derek Tutssel (video director), Lih-Mei Liao, Wendy Harris, Steve Saunders, Ruth Canter, Rahi Jahan, Blair McLennan, Emma Wood. David Aaronovitch, Sheila Richards, Peter Cross, Rajadurai Sunderalingham, Kate Baden-Fuller, Caroline Knight, and other members of the UCLH patients and family advisory group.

"POPPI" nurses and site PIs:

University College Hospital, London: Emma Davies, Emma Thompsett, Rosalind Edwards, David Brealey, Magda Rocha, Jung Ryu, Georgia Bercades. Watford General Hospital: XioBei Zhao; Jennie Haydock, Kalpana Giri Ghimire, Lillian Norris, James Cunningham, Sarah- Jane Turner, Tracey Temple

Medway Maritime Hospital: Catherine Plowright, Claire Pegg, James Cullinane

Bristol Royal Infirmary: Katie Sweet, Sanjoy Shah, Lisa Grimmer.

Funding

This project was funded by the National Institute for Health Research (NIHR) Health Services and Delivery Research (HS&DR) Programme (Project: 12/64/124). DW also received funding from the University College London/University College London Hospitals NIHR Biomedical Research Centre. The views and opinions expressed in this paper are those of the authors and do not necessarily reflect those of the HS&DR, NIHR, NHS or the Department of Health. The funding body had no role in the design of the study and collection, analysis, and interpretation of data and in writing the manuscript.

Ethics

The two feasibility studies were granted favourable ethical opinion by the NRES Committee South Central - Oxford B research ethics committee on 23 April 2014 (reference: 14/SC/0149), were managed by the ICNARC CTU and prospectively registered on the ISRCTN Registry (ISRCTN61088114).

Transparency Declaration

The lead author affirms that the manuscript is an honest, accurate transparent account of the study being reported, that no important aspects of the study have been omitted, and that any discrepancies from the study as planned, have been explained.

Copyright

The Corresponding Author has the right to grant on behalf of all authors and does grant on behalf of all authors, a worldwide licence to the Publishers and its licensees in perpetuity, in all forms, formats and media

For peer review only - http://bmjopen.bmj.com/site/about/guidelines.xhtml

For peer review only

(whether known now or created in the future), to i) publish, reproduce, distribute, display and store the Contribution, ii) translate the Contribution into other languages, create adaptations, reprints, include within collections and create summaries, extracts and/or, abstracts of the Contribution, iii) create any other derivative work(s) based on the Contribution, iv) to exploit all subsidiary rights in the Contribution, v) the inclusion of electronic links from the Contribution to third party material where-ever it may be located; and, vi) licence any third party to do any or all of the above. We attest that we have obtained appropriate permissions and paid any required fees for use of copyright protected materials.

Competing interests

All authors have completed the ICMJE uniform disclosure form at www.icmje.org/coi_disclosure.pdf and declare: no support from any organisation for the submitted work; no financial relationships with any organisations that might have an interest in the submitted work in the previous three years; no other relationships or activities that could appear to have influenced the submitted work."

Data sharing

No additional data available

References

1. Wade DM. Doctoral thesis: Prevalence and predictors of psychological morbidity and quality of life after discharge from Intensive Care. University College London, 2010.
2. Puntillo KA, Arai S, Cohen NH, et al. Symptoms experienced by intensive care unit patients at high risk of dying. Critical care medicine 2010;**38**(11):2155-60.
3. Novaes MA, Knobel E, Bork AM, et al. Stressors in ICU: perception of the patient, relatives and health care team. Intensive care medicine 1999;**25**(12):1421-6.
4. Alonso-Ovies A, Heras La Calle G. ICU: a branch of hell? Intensive care medicine 2016;**42**(4):591-2.
5. Nelson JE. The symptom burden of chronic critical illness. Critical care medicine 2004;**32**.
6. Jones C, Griffiths RD, Humphris G, et al. Memory, delusions, and the development of acute posttraumatic stress disorder-related symptoms after intensive care. Critical care medicine 2001;**29**(3):573-80.
7. Wade DM, Howell DC, Weinman JA, et al. Investigating risk factors for psychological morbidity three months after intensive care: a prospective cohort study. Critical care (London, England) 2012;**16**(5):R192.
8. Parker AM, Sricharoenchai T, Raparla S, et al. Posttraumatic stress disorder in critical illness survivors: a metaanalysis. Critical care medicine 2015;**43**(5):1121-9.
9. Nikayin S, Rabiee A, Hashem MD, et al. Anxiety symptoms in survivors of critical illness: a systematic review and meta-analysis. General hospital psychiatry 2016;**43**:23-29.
10. Rabiee A, Nikayin S, Hashem MD, et al. Depressive Symptoms After Critical Illness: A Systematic Review and Meta-Analysis. Critical care medicine 2016;**44**(9):1744-53.
11. Huang M, Parker AM, Bienvenu OJ, et al. Psychiatric Symptoms in Acute Respiratory Distress Syndrome Survivors: A 1-Year National Multicenter Study. Critical care medicine 2016.
12. Bienvenu OJ, Colantuoni E, Mendez-Tellez PA, et al. Cooccurrence of and remission from general anxiety, depression, and posttraumatic stress disorder symptoms after acute lung injury: a 2-year longitudinal study. Critical care medicine 2015;**43**(3):642-53.
13. National Institute of Health and Care Excellence (NICE) quality standard 158 - Rehabilitation after critical illness in adults: NICE, 2017
14. Cuthbertson BH, Rattray J, Campbell MK, et al. The PRaCTICaL study of nurse led, intensive care follow-up programmes for improving long term outcomes from critical illness: a pragmatic randomised controlled trial. BMJ (Clinical research ed) 2009;**339**:b3723.
15. Jensen JF, Thomsen T, Overgaard D, et al. Impact of follow-up consultations for ICU survivors on post-ICU syndrome: a systematic review and meta-analysis. Intensive care medicine 2015;**41**(5):763-75.
16. Mehlhorn J, Freytag A, Schmidt K, et al. Rehabilitation interventions for postintensive care syndrome: a systematic review. Critical care medicine 2014;**42**(5):1263-71.

For peer review only - http://bmjopen.bmj.com/site/about/guidelines.xhtml

17. Jensen JF, Egerod I, Bestle MH et al. (2016) A recovery program to improve quality of life, sense of coherence and psychological health in ICU survivors: a multicenter randomized controlled trial, the RAPIT study. Intensive Care Med, 42(11): 1733-43.
18. Jones C, Backman C, Capuzzo M, Egerod I, Flatten H, Granja C et al. Intensive care diaries reduce new-onset post traumatic stress disorder following critical illness: a randomised, controlled trial. Crit Care 2010; 14:R168
19. Cox CE, Hough CL, Carson SS, White DB, Kahn JM, Olsen MK, et al. Effects of a telephone- and web-based coping skills training program compared with an education program for survivors of critical illness and their family members: a randomized clinical trial. Am J Respir Crit Care Med 2018;197:66–78.
20. Davydow DS, Zatzick D, Hough CL, et al. In-hospital acute stress symptoms are associated with impairment in cognition 1 year after intensive care unit admission. Annals of the American Thoracic Society 2013;**10**(5):450-7.
21. Jones C, Backman C, Capuzzo M, et al. Precipitants of post-traumatic stress disorder following intensive care: a hypothesis generating study of diversity in care. Intensive care medicine 2007;**33**(6):978-85.
22. Peris A, Bonizzoli M, Iozzelli D, et al. Early intra-intensive care unit psychological intervention promotes recovery from post traumatic stress disorders, anxiety and depression symptoms in critically ill patients. Critical care (London, England) 2011;**15**(1):R41.
23. Papathanassoglou ED. Psychological support and outcomes for ICU patients. Nursing in critical care 2010;**15**(3):118-28.
24. Wade DF, Moon Z, Windgassen SS, et al. Non-pharmacological interventions to reduce ICU-related psychological distress: a systematic review. Minerva anestesiologica 2016;**82**(4):465-78.
25. Fowler D, Garety P, Kuipers E. *Cognitive behaviour therapy for psychosis: theory and practice.* Chichester: John Wiley and Sons, 1995.
26. van der Gaag M, Valmaggia LR, Smit F. The effects of individually tailored formulation-based cognitive behavioural therapy in auditory hallucinations and delusions: A meta-analysis. Schizophrenia Research 2014; **156** (1): 30-37
27. Hobfoll S, Watson P, Bell C, et al. Five Essential Elements of Immediate and Mid-Term Mass Trauma Intervention: Empirical Evidence. FOCUS 2009;**7**(2):221-42.
28. Brewin CR. Re-experiencing traumatic events in PTSD: new avenues in research on intrusive memories and flashbacks. Eur J Psychotraumatol 2015;**6**:27180.
29. Craig P, Dieppe P, Macintyre S, et al. Developing and evaluating complex interventions: the new Medical Research Council guidance. BMJ (Clinical research ed) 2008;**337**:a1655.
30. Wade D, Hardy R, Howell D, et al. Identifying clinical and acute psychological risk factors for PTSD after critical care: a systematic review. Minerva anestesiologica 2013;**79**(8):944-63.
31. Wade DM. MSc thesis: The psychology of weaning from mechanical ventilation: a mixed methods study of mood, control, support and communication in the intensive care unit. University of London, 2006.
32. Wade DM. Portfolio of competence, stage II health psychology: The British Psychological Society, 2011.
33. Wade DM, Brewin CR, Howell DC, et al. Intrusive memories of hallucinations and delusions in traumatized intensive care patients: An interview study. British journal of health psychology 2014.
34. Wade DM, Hankins M, Smyth DA, et al. Detecting acute distress and risk of future psychological morbidity in critically ill patients: validation of the intensive care psychological assessment tool. Critical care (London, England) 2014;**18**(5):519.
35. Wade D, Moon Z, Windgassen S, et al. Nonpharmacological interventions to reduce short-term or long-term psychological stress in ICU patients: a systematic review. Critical Care 2015;**19**(Suppl 1):P556-P56.
36. Lazarus RS, Folkman S. *Stress, appraisal, and coping.* New York, NY: Springer Publishing Company, 1984.
37. Silverman J, Kurtz S, Draper J. *Skills for Communicating with Patients*. Oxford: Radcliffe Publishing, 2013.
38. Bowers L, Brennan G, Winship G, et al. How expert nurses communicate with acutely psychotic patients. Mental Health Practice 2010;**13**(7):24-26.
39. Michie S, van Stralen MM, West R. The behaviour change wheel: a new method for characterising and designing behaviour change interventions. Implementation science 2011;**6**:42.
40. Stoltenberg CD, McNeill B, Delworth U. *IDM supervision: An integrated developmental model for supervising counselors and therapists.* San Francisco: Jossey-Bass, 1998.
41. Bullock I, Davis M, Lockey A, et al. *Pocket Guide to Teaching for Clinical Instructors*. 3 ed. West Sussex: John Wiley & Sons Ltd., 2016.
42. Kirkpatrick DL, Kirkpatrick JD. *Implementing the Four Levels*. San Francisco, CA: Berrett-Koehler Publishers, 2007.
Sessler CN, Gosnell MS, Grap MJ, et al.

43. The Richmond Agitation-Sedation Scale: validity and reliability in adult intensive care unit patients. American journal of respiratory and critical care medicine 2002;**166**(10):1338-44.
44. Foa EB, Cashman L, Jaycox L, et al. The validation of a self-report measure of posttraumatic stress disorder: the Posttraumatic Diagnostic Scale. . Psychological Assessment 1997;**9**:445-51.
45. Irwin M, Artin KH, Oxman MN. Screening for depression in the older adult: criterion validity of the 10-item Center for Epidemiological Studies Depression Scale (CES-D). Archives of internal medicine 1999;**159**(15):1701-4.

For peer review only - http://bmjopen.bmj.com/site/about/guidelines.xhtml

Figure legends

Figure one: Use of Medical Research Council framework for developing and evaluating complex interventions

Figure two: Patient flow in the intervention feasibility study

Figure three: CONSORT diagram for the trial procedures feasibility study

For peer review only

For peer review only - http://bmjopen.bmj.com/site/about/guidelines.xhtml

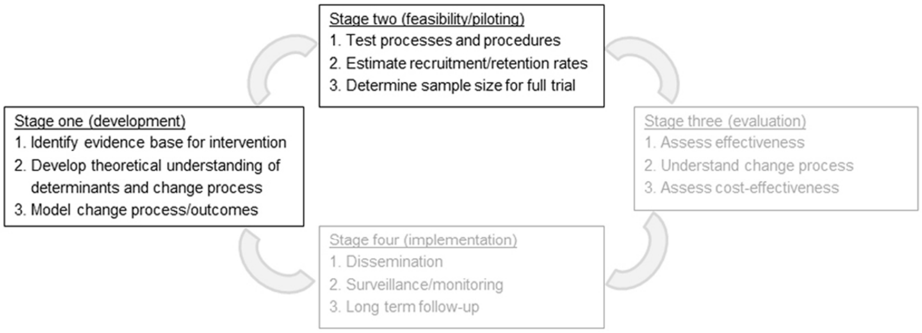

Figure one: Use of Medical Research Council framework for developing and evaluating complex interventions

124x93mm (300 x 300 DPI)

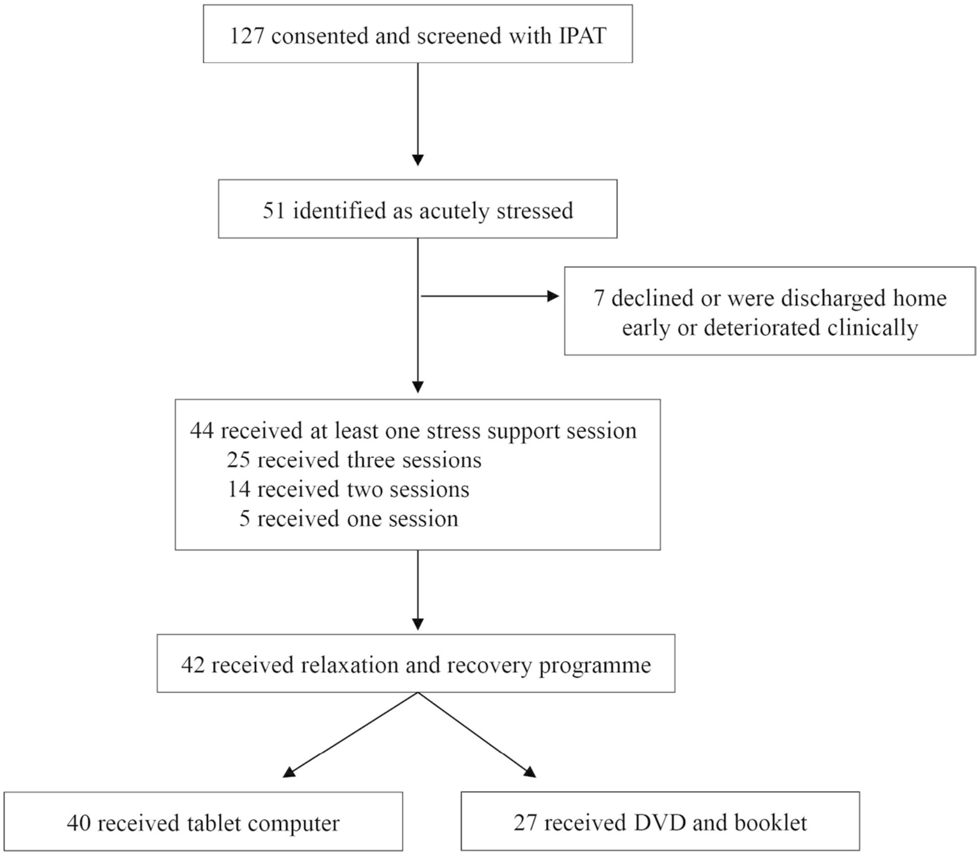

Figure two: Patient flow in the intervention feasibility study

124x110mm (300 x 300 DPI)

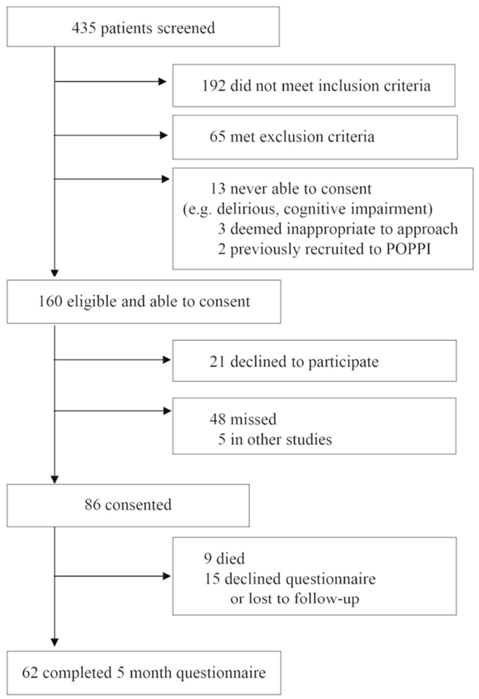

Figure three: CONSORT diagram for the trial procedures feasibility study

124x93mm (300 x 300 DPI)

For peer review only

Electronic supplement one: Development phases of an intervention to reduce patient stress in critical care

**MRC model – steps and stages**	**Preparatory phase 2006-11***	**Early modelling 2011-13†**	**Feasibility and refinement phase 2013-2015‡**
Stage one, step one<br>Identify the evidence base	Identified systematic reviews and studies of psychological outcomes, risk factors and conducted new systematic reviews of post-traumatic stress, depression, anxiety post-ICU[1]<br><br>Literature review of ICU stressors, reactions, memories, stress measurement and stress reduction interventions[1]	Updated evidence base - identified new systematic reviews and studies of ICU psychological outcomes, risk factors, stress, stress measurement, stress reduction interventions[7,20,22]<br><br>Began systematic review of psychosocial interventions (psychological, music, mind-body therapies) to reduce ICU-related stress[24,35]	Updated evidence base of studies and systematic reviews[8,12,33]<br><br>Completed systematic review of interventions to reduce critical care stress[24,35]
Stage one, step two<br>Develop a theoretical understanding of determinants and processes of change	Patient studies: acute stress in ICU and psychological outcomes:<br>1. Quantitative study (n=40)[31]<br>2. Quantitative study (n=157)[7]<br>3. Qualitative study (n=7) [31]<br>4. Qualitative study (n=17)[33]<br><br>Developed hypotheses about causes of critical stress[1,7,31,32]<br><br>Explored nurse perceptions of patients' psychological needs and nurses' ability to attend to them: Content analysis of 5 focus groups at GSTT NHS trust with 125 nurses[32]<br><br>Identified communication[37] and adult learning methods** to inform development of training to enhance critical care nurse communication and psychological skills	Patient and relatives advisory group set up at UCLH; regular meetings to brainstorm and generate research ideas about patients' psychological needs and how they could be met<br><br>Improved conceptualisation and measurement of acute ICU stress: Development and validation of the Intensive care psychological assessment tool[34]<br><br>Identified psychological approaches (to stress and coping[36], psychosis[25-26] and trauma[27-28]) that could be adapted for critical care patients<br><br>Identified appropriate relaxation and recovery methods	Identified behaviour change methods[39] relevant to changing staff behaviours to create a therapeutic environment<br><br>Identified clinical supervision methods for non-experts delivering psychological support[40]
Stage one, step three<br>Model process and outcomes	Developed, piloted and evaluated a training day at GSTT to train critical care nurses in communication/psychological skills (utilising actors)[32]	Consultation with experts and discussions between UCLH, ICNARC and patient representatives, to develop the basic structure of a psychological intervention consisting of three interconnected elements: Creating a therapeutic environment; three stress support sessions; and a relaxation and recovery programme. Each element to have associated training components (online, face-to-face and training materials)<br><br>Early development of stress support sessions based on psychological principles at	Formed Expert Psychological Advisory Group of medical educationalist, psychologists, patients and the research team, to inform the final phase of POPPI†† intervention development<br><br>Further development of stress support sessions by senior nurses and psychologists<br><br>Created materials to deliver a complex intervention - nurse manual, checklists, notes and summaries<br><br>Researched/created content for and design of a relaxation and recovery app, a DVD and a

For peer review only

		UCLH – iterative process with psychologist and patients, supervised by senior psychologists (CB, JW, LML and staff from PICUP)<br><br>Identification of suitable materials for the relaxation/recovery programme<br><br>Post-traumatic stress selected as primary outcome (based on evidence identified in step one – more evidence of risk factors than for other psychological outcomes)	psychological well-being booklet for patients<br><br>Created training courses and materials for staff to deliver the psychological intervention:<br>Online training course, all staff<br>3-day face to face course for POPPI nurses, feedback/assessment day , training folder<br>A programme of debriefing and support for POPPI nurses
Stage two Feasibility and Piloting		Designed two separate feasibility studies – intervention feasibility study and the trial procedures feasibility study	Conducted two feasibility studies – intervention feasibility study and trial procedure study<br><br>Refined the complex intervention based on quantitative and qualitative data from patients and staff in the intervention feasibility study

**Kings College London, Guys and St Thomas hospitals Trust, University College/University College Hospital, London*
†University College Hospital (UCH) and Intensive Care National Audit and Research Centre (ICNARC);
‡ ICNARC and UCH
*** 43. Kolb DA. Experiential learning: experience as the source of learning and development. 1 ed. Englewood Cliffs, NJ: Prentice Hall, 1984.*
†† POPPI – Provision Of Psychological support to People in Intensive care
CB Chris Brewin (author), JW John Weinman (author) LML Lih-Mei Liao, Consultant Clinical Psychologist, University College London Hospitals (UCLH) . PICUP -Psychological interventions clinic for outpatients with psychosis, Maudesley Psychology Centre, Maudesley Hospital, London

For peer review only

Electronic supplement two

This supplement presents further details of the final post-feasibility version of the three-element POPPI complex intervention. This version is being evaluated in the POPPI cRCT.

Element one: Creating a therapeutic environment

Online training course
A key part of element one is the POPPI online training course: Key skills in Psychological Care. Training is undertaken by all clinical critical care staff to aid the creation of a calm, less stressful environment by reducing stressors in the environment and using good communication with patients. The course takes about 30 minutes and comprises the following five sections:

1 Understanding the stresses of intensive care patients
2 Reducing stress and fear in patients
3 Communicating with distressed patients
4 Inspiring patients with confidence and hope
5 Summary and assessment

The course has a balance of concise, readable text with graphics and other visual or audio aids, and test-yourself questions at the end of each section (with informative feedback). Video clips include former patients talking about their experiences of critical care, and nurses modelling good communication strategies with patients. Staff who pass the assessment (a score of 80% or more) receive a certificate.

Element two: Three stress support sessions

Three, one-to-one, stress support sessions lasting approximately 30 minutes each, are delivered to a patient by the same trained POPPI nurse, starting when patients are awake and alert, either in the critical care unit or following discharge to the hospital ward. The three sessions should be delivered within a week (for the pragmatic reason that many patients may be discharged from hospital around that time).

All stress support sessions contain three common components – starting the session; building rapport and finishing the session - and three individual components per session. Starting a session includes introductions, outlining aims, measuring stress and explaining confidentiality, note-taking and risk-reporting. Building rapport involves focusing throughout all the sessions on creating a relationship with the patient based on openness, honesty, warmth and listening. Finishing a session involves summarising, getting patient feedback, agreeing practice, re-measuring stress, agreeing wording for notes and arranging the next session.

The three individual components nurses deliver in session one are: Normalise reactions (discuss common psychological reactions in critical care and possible reasons); Encourage communication (help patients open up about worries and concerns), and Teach coping strategies, e.g. patients gathering more information and practising relaxation exercises (acronym NET). The second session comprises: Stress reactions (encourage further expression of worries and notice stressful thoughts); Explain stressful thinking (show how unrealistic fears can create extra stress), and Teach 'check out my fear', a technique to understand how accurate fears are (SET). The third session includes: Summarise and review (key messages from sessions and persisting issues); Action plan (personal planning to build on sessions and cope with challenges ahead) and Future Expectations (reinforce progress made and encourage realistic optimism for psychological and physical recovery (acronym SAFE).

For peer review only

Education package to deliver element two

The education package to train POPPI nurses to deliver element two includes a central three-day face-to-face training course with associated materials; a local debriefing and support visit, including a skills development assessment, and ongoing debriefing and support phone calls from trainers.

The course involves pre-course study (a psychological principles booklet, a manual for the three stress support sessions and access to the POPPI online training course) and a three-day programme, as follows:

Day one
- Understanding critical care patients' stress
- Learning the skills needed to deliver stress support sessions
- Overview of the stress support sessions
- Observing and practising session one

Day two
- More about unusual experiences in critical care
- Learning further stress support skills
- Observing and practising stress support session two

Day three
- Revision of key points from stress support session two
- Observing and practising stress support session three
- Using the patient booklet to create personal action plans
- Debriefing and support arrangements

Course materials include training folder with day 1-3 course handouts, word and visual summaries of each session, checklists and reflective notes for each session, stress thermometer tool, copies of patient booklet and DVD.

Debriefing and support

The first debriefing and support call should occur soon after (or during, if nurse requests) the three stress support sessions nurses deliver to their first POPPI patient. Subsequent calls are every two months, or on nurses' request. The focus of these calls is on enhancing nurses' skills and discussing patient cases. Emails can be exchanged between nurses and trainers about issues arising from stress support sessions at any time. Monthly teleconferences with POPPI nurses are facilitated by a trainer to encourage peer support.

A debriefing and support visit is made by an allocated trainer to offer the three POPPI nurses at one site further support, both individually and in a group. Later in the visit a skills development assessment is carried out for each nurse. Using a scenario, the nurse delivers session 2 to the trainer who plays the role of the patient. The trainer rates the nurse's skills using a checklist. The pass mark is 8/16. Nurses are required to pass the assessment, to continue delivering stress support sessions to further patients.

Element Three: Relaxation and recovery programme

In addition to stress support sessions, patients receive a "relaxation and recovery" programme (using a tablet computer app between sessions, and a DVD and patient booklet to take home after hospital) to help them to sleep and cope better with stress. Former intensive care patients' recovery stories are included to help normalise critical care experiences and reactions, and encourage hope and optimism for recovery. Contents also include audio or video files of a safe place visualisation, muscle relaxation and breathing exercises; a body scan and other mindfulness meditations; relaxing classical music from Bach to Vivaldi, calming modern ambient music; and restful nature sounds and scenes.

For peer review only

The programme was first developed as an application for tablet computers. The design of the app includes a green nature-scene background and large coloured buttons for patients to use for easy navigation between different sections. The tablet computers should be checked for approval by hospital infection control teams. DVDs include a shorter selection of relaxation exercises and music from the app, due to restricted space. However they include longer versions of the patient recovery stories, assuming longer concentration spans as patients get closer to hospital discharge or go home.

The "Getting well, staying well" booklet provides patients with information on making a good psychological recovery after a critical care stay. It builds on the support patients received during the stress support sessions, focusing on well-being and coping strategies. Contents include information about what to expect in the early days (leaving critical care, returning home; emotions after critical care, relationships), tips for psychological wellbeing; advice on coping with difficulties (worries, panic, low mood, memories); information on sources of psychological support; further information for family and friends and a personal action plan to promote well-being and recovery.

For peer review only

CONSORT 2010 checklist of information to include when reporting a pilot or feasibility trial*

Section/Topic	Item No	Checklist item	Reported on page No
**Title and abstract**			
	1a	Identification as a pilot or feasibility randomised trial in the title	n/a
	1b	Structured summary of pilot trial design, methods, results, and conclusions (for specific guidance see CONSORT abstract extension for pilot trials)	2
**Introduction**			
Background and objectives	2a	Scientific background and explanation of rationale for future definitive trial, and reasons for randomised pilot trial	Scientific rationale p3 (but not a randomised trial)
	2b	Specific objectives or research questions for pilot trial	p4, para 1
**Methods**			
Trial design	3a	Description of pilot trial design (such as parallel, factorial) including allocation ratio	4, 9/10
	3b	Important changes to methods after pilot trial commencement (such as eligibility criteria), with reasons	n/a
Participants	4a	Eligibility criteria for participants	9
	4b	Settings and locations where the data were collected	9
	4c	How participants were identified and consented	9
Interventions	5	The interventions for each group with sufficient details to allow replication, including how and when they were actually administered	5-8
Outcomes	6a	Completely defined prespecified assessments or measurements to address each pilot trial objective specified in 2b, including how and when they were assessed	9-10
	6b	Any changes to pilot trial assessments or measurements after the pilot trial commenced, with reasons	n/a
	6c	If applicable, prespecified criteria used to judge whether, or how, to proceed with future definitive trial	n/a
Sample size	7a	Rationale for numbers in the pilot trial	n/a
	7b	When applicable, explanation of any interim analyses and stopping guidelines	n/a
Randomisation:			
Sequence generation	8a	Method used to generate the random allocation sequence	n/a
	8b	Type of randomisation(s); details of any restriction (such as blocking and block size)	n/a

For peer review only

Allocation concealment mechanism	9	Mechanism used to implement the random allocation sequence (such as sequentially numbered containers), describing any steps taken to conceal the sequence until interventions were assigned	n/a
Implementation	10	Who generated the random allocation sequence, who enrolled participants, and who assigned participants to interventions	n/a
Blinding	11a	If done, who was blinded after assignment to interventions (for example, participants, care providers, those assessing outcomes) and how	n/a
	11b	If relevant, description of the similarity of interventions	n/a
Statistical methods	12	Methods used to address each pilot trial objective whether qualitative or quantitative	9/10
**Results**			
Participant flow (a diagram is strongly recommended)	13a	For each group, the numbers of participants who were approached and/or assessed for eligibility, randomly assigned, received intended treatment, and were assessed for each objective	Figures 2/3
	13b	For each group, losses and exclusions after randomisation, together with reasons	"
Recruitment	14a	Dates defining the periods of recruitment and follow-up	10/14
	14b	Why the pilot trial ended or was stopped	n/a
Baseline data	15	A table showing baseline demographic and clinical characteristics for each group	n/a
Numbers analysed	16	For each objective, number of participants (denominator) included in each analysis. If relevant, these numbers should be by randomised group	Figures 2/3
Outcomes and estimation	17	For each objective, results including expressions of uncertainty (such as 95% confidence interval) for any estimates. If relevant, these results should be by randomised group	Table 2 (pp12/13)
Ancillary analyses	18	Results of any other analyses performed that could be used to inform the future definitive trial	P14
Harms	19	All important harms or unintended effects in each group (for specific guidance see CONSORT for harms)	n/a
	19a	If relevant, other important unintended consequences	n/a
**Discussion**			
Limitations	20	Pilot trial limitations, addressing sources of potential bias and remaining uncertainty about feasibility	15
Generalisability	21	Generalisability (applicability) of pilot trial methods and findings to future definitive trial and other studies	n/a
Interpretation	22	Interpretation consistent with pilot trial objectives and findings, balancing potential benefits and harms, and considering other relevant evidence	p15
	22a	Implications for progression from pilot to future definitive trial, including any proposed amendments	p14
**Other information**			
Registration	23	Registration number for pilot trial and name of trial registry	p6
Protocol	24	Where the pilot trial protocol can be accessed, if available	

For peer review only

Funding	25	Sources of funding and other support (such as supply of drugs), role of funders	16
	26	Ethical approval or approval by research review committee, confirmed with reference number	9/16

Citation: Eldridge SM, Chan CL, Campbell MJ, Bond CM, Hopewell S, Thabane L, et al. CONSORT 2010 statement: extension to randomised pilot and feasibility trials. BMJ. 2016;355.

*We strongly recommend reading this statement in conjunction with the CONSORT 2010, extension to randomised pilot and feasibility trials, Explanation and Elaboration for important clarifications on all the items. If relevant, we also recommend reading CONSORT extensions for cluster randomised trials, non-inferiority and equivalence trials, non-pharmacological treatments, herbal interventions, and pragmatic trials. Additional extensions are forthcoming: for those and for up to date references relevant to this checklist, see www.consort-statement.org.

For peer review only
